# Copper-catalysed perarylation of cyclopentadiene: synthesis of hexaarylcyclopentadienes†

**DOI:** 10.1039/d4sc02458c

**Published:** 2024-05-16

**Authors:** Yohan Gisbert, Pablo Simón Marqués, Caterina Baccini, Seifallah Abid, Nathalie Saffon-Merceron, Gwénaël Rapenne, Claire Kammerer

**Affiliations:** a CEMES, Université de Toulouse, CNRS 29 Rue Marvig 31055 Toulouse France claire.kammerer@cemes.fr; b Université de Toulouse, UPS, Institut de Chimie de Toulouse ICT UAR 2599, 118 Route de Narbonne 31062 Toulouse France; c Division of Materials Science, Nara Institute of Science and Technology 8916-5 Takayama, Ikoma Nara Japan

## Abstract

While hexaphenylsilacyclopentadiene (hexaphenylsilole) is viewed as an archetypal Aggregation-Induced Emission (AIE) luminogen, its isostructural hydrocarbon surrogate hexaphenylcyclopentadiene has strikingly never been investigated in this context, most probably due to a lack of synthetic availability. Herein, we report a straightforward synthesis of hexaphenylcyclopentadiene, *via* the direct perarylation of cyclopentadiene upon copper(i) catalysis under microwave activation, with the formation of six new C–C bonds in a single synthetic operation. Using zirconocene dichloride as a convenient source of cyclopentadiene and a variety of aryl iodides as coupling partners, this copper-catalysed cross-coupling reaction gave rise to a series of unprecedented hexaarylcyclopentadienes. The latter are direct precursors of extended π-conjugated polycyclic compounds, and their cyclodehydrogenation under Scholl reaction conditions yielded helicenic 17,17-diarylcyclopenta[*l*,*l*′]diphenanthrenes. These structurally complex polyannelated fluorene derivatives can now be prepared in only two synthetic steps from cyclopentadiene.

## Abstract 2

The development of a copper-catalysed direct hexaarylation of cyclopentadiene under microwave activation allowed the synthesis of a family of unprecedented hexaarylcyclopentadienes.

## Graphical abstract

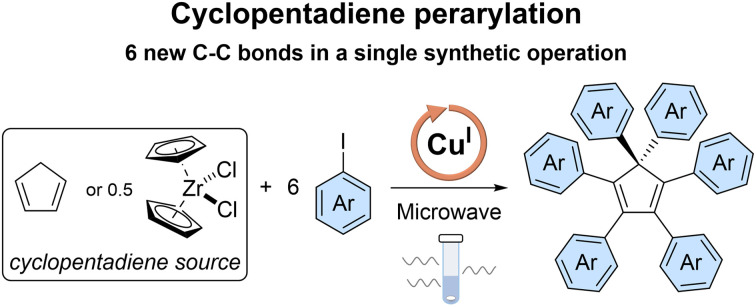

## Introduction

Group-14 metallacyclopentadienes have attracted the attention of the scientific community for decades, with their first syntheses dating back to the early 1960s.^1,2^ Among them, the silicon derivatives, namely siloles, display remarkable electronic properties such as high electron affinity and electron mobility, coupled to excellent photo- and electroluminescence. As a consequence, these building blocks have been widely used in electron-transporting and light-emitting materials for the fabrication of organic optoelectronic devices.^3,4^ Whereas most organic dyes suffer from aggregation-caused quenching of their emission, thus inherently limiting their efficiency as organic light-emitting diode (OLED) materials, B. Z. Tang *et al.* disclosed in 2001 the Aggregation-Induced Emission (AIE) behaviour of propeller-shaped silacyclopentadienes such as penta- and hexaphenylsilole.^2,5–7^ These compounds exhibit negligible emission in dilute solutions as opposed to an intense photoluminescence in the aggregate or solid state, which originates from restricted intramolecular motions in combination with limited intermolecular π–π stacking, thus favouring radiative relaxation from excited states.^8^ Hexaphenylsilole is thus viewed as an archetypal AIE luminogen, and structural variations in polyarylsilacyclopentadienes progressively allowed modulation of their properties,^9^ leading to various applications as materials in high-performance OLEDs, chemosensors for analyte detection, biological probes and smart materials.^10^

**Scheme 1:**
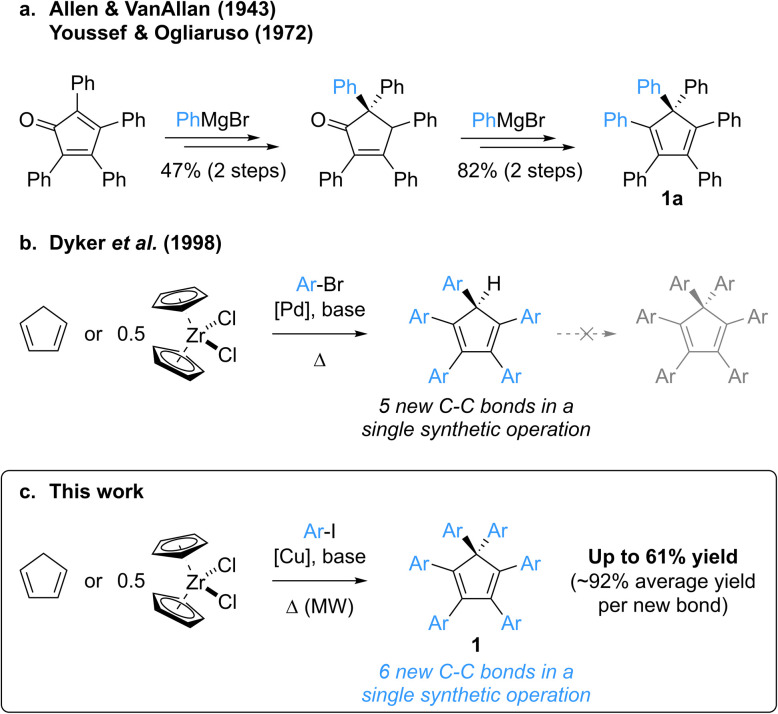
Perarylated cyclopentadienes: reported and envisioned synthetic strategies. (a) Multistep synthesis of hexaphenylcyclopentadiene 1a.^12,13^ (b) Palladium-catalysed direct arylation of cyclopentadiene yielding pentaarylcyclopentadienes in a single step.^20^ (c) Copper-catalysed microwave-activated perarylation of cyclopentadiene yielding hexaarylcyclopentadienes in a single step.

Over the years, a variety of architectures have been reported to be AIE active,^8,10*a*,10*b*^ including polyarylcyclopentadienes Ar_*n*_H_(6−*n*)_Cp (*n* = 3–5) as pure hydrocarbon propeller-shaped systems.^11^ Strikingly, despite being an isostructural hydrocarbon surrogate of hexaphenylsilole hexaphenylcyclopentadiene Ph_6_Cp (1a, Scheme 1) has never been investigated in this context, most probably due to the lack of synthetic availability of this compound and of its aryl-substituted derivatives. To the best of our knowledge, the synthesis of hexaphenylcyclopentadiene has only been reported once, by Allen and VanAllan in 1943,^12^ with a revision of the mechanism and synthetic intermediate structures published in 1972 by Youssef and Ogliaruso.^13,14^ Hexaphenylcyclopentadiene 1a was prepared in four steps and 39% overall yield starting from tetraphenylcyclopentadienone, with two 1,2-additions of phenylmagnesium bromide as key steps and a thermally-induced [1,5] sigmatropic phenyl shift accounting for the generation of the *gem*-diphenyl-substituted sp^3^ carbon (Scheme 1a). According to the reaction conditions, this synthetic route appears tedious, with limited functional group compatibility and a low modularity related to the presence of four, out of six, aryl substituents in the initial cyclopentadienone.

In contrast with hexaarylcyclopentadienes, the pentaarylated counterparts have been tremendously studied, especially as neutral precursors of (super-)bulky cyclopentadienyl ligands in the field of coordination chemistry,^15^ but also for the elaboration of new molecular machines incorporating propeller-shaped rotating subunits, such as motors,^16^ gears^17^ or winches.^18^ In this context, various strategies for the multistep synthesis of polysubstituted cyclopentadienes have been devised,^19^ the prominent one being the “tetracyclone route”, which involves an aryl addition onto a cyclopentadienone key intermediate and the subsequent reduction to the corresponding cyclopentadiene. In 1998, Dyker, Miura *et al.* reported a conceptually-novel approach for the synthesis of symmetrical pentaarylcyclopentadienes, involving the five-fold direct arylation of cyclopentadiene under palladium catalysis (Scheme 1b).^20^ This powerful method, leading to the formation of five new C–C bonds in a single synthetic operation, tolerates a large variety of aryl bromides as coupling partners (including highly sterically demanding ones^15*b*,15*i*,21^) and zirconocene dichloride can be employed as a convenient source of cyclopentadiene.

Attracted by the efficiency and modularity of this approach for the synthesis of our molecular machine prototypes,^16–18^ we envisioned to develop a greener alternative involving copper(i) as a cheap, abundant and environmentally-benign catalyst for the direct arylation of cyclopentadiene. Indeed, the intrinsic stability of the cyclopentadienide anion acting as nucleophile in such C–C coupling was reminiscent of the well-established Hurtley reaction, allowing the copper-mediated arylation of stabilised carbon nucleophiles such as active methylenes.^22,23^ Much to our surprise, our initial attempts of cyclopentadiene arylation in the presence of copper(i), using excess iodobenzene as coupling partner, resulted in the formation of a new product, aside from the expected pentaphenylcyclopentadiene. It turned out that the latter can undergo a copper-mediated direct arylation under basic conditions to yield hexaphenylcyclopentadiene 1a. Given its structural proximity with hexaphenylsilole and, more generally, the promising potential of hexaarylcyclopentadienes as AIE luminogens for the fabrication of high-performance optoelectronic devices, we initiated a project aiming at the synthesis of such target compounds *via* a direct six-fold arylation of cyclopentadiene (Scheme 1c).

Herein we report the copper(i)-catalysed direct perarylation of cyclopentadiene, giving rise to a series of unprecedented hexaarylcyclopentadienes as the result of the formation of six new C–C bonds in a single synthetic operation. The structural and optical properties of such perarylated cyclopentadienes are unveiled, with an emphasis on their behaviour as AIE luminogens, and their direct conversion into π-extended helical scaffolds such as tetrabenzofluorenes is addressed.

## Results and discussion

**Scheme 2:**
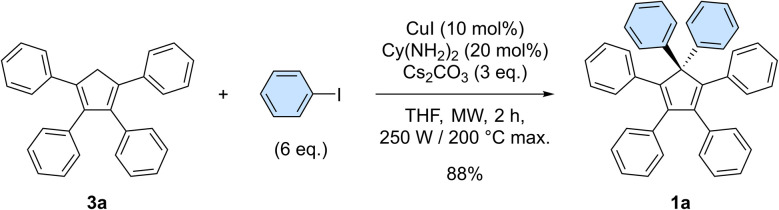
Optimised conditions for the model reaction, involving the copper-catalysed diarylation of tetraphenylcyclopentadiene 3a to yield hexaphenylcyclopentadiene 1a. Reagents and conditions: 3a (0.5 mmol), iodobenzene (3 mmol, 6 eq.), Cs_2_CO_3_ (1.5 mmol, 3 eq.), CuI (0.05 mmol, 10 mol%), (±)-*trans*-1,2-cyclohexanediamine Cy(NH_2_)_2_ (0.10 mmol, 20 mol%), THF (2 mL), microwave irradiation (250 W available), 200 °C max., 2 h.

In our preliminary attempts to achieve copper-catalysed direct arylation of cyclopentadiene, penta- and hexaarylcyclopentadienes were produced in very low yields after long reaction times under conventional thermal activation, along with numerous side-products. The latter most probably result from incomplete 1- to 4-fold arylation processes, but also from competitive Diels–Alder [4 + 2] cycloadditions of cyclopentadiene and its partially arylated derivatives. To hinder this unproductive pathway, arylation reactions were next carried out at high temperature under microwave irradiation, so as to favour *in situ* retro Diels–Alder reactions. In addition, considering that the most difficult steps in the direct perarylation of cyclopentadiene are the fifth and sixth C–C couplings due to major steric hindrance, the copper-catalysed arylation of tetraphenylcyclopentadiene (3a) using iodobenzene as coupling partner was investigated as a model reaction (Scheme 2). A single arylation thus affords pentaphenylcyclopentadiene (2a), which can subsequently undergo a second C–C coupling to yield the desired hexaphenylcyclopentadiene 1a. This system considerably simplifies the optimisation process, since only two different arylation products may be formed and any competitive Diels–Alder reaction is avoided due to the bulkiness of the involved polyphenylcyclopentadiene species.

**Fig. 1:**
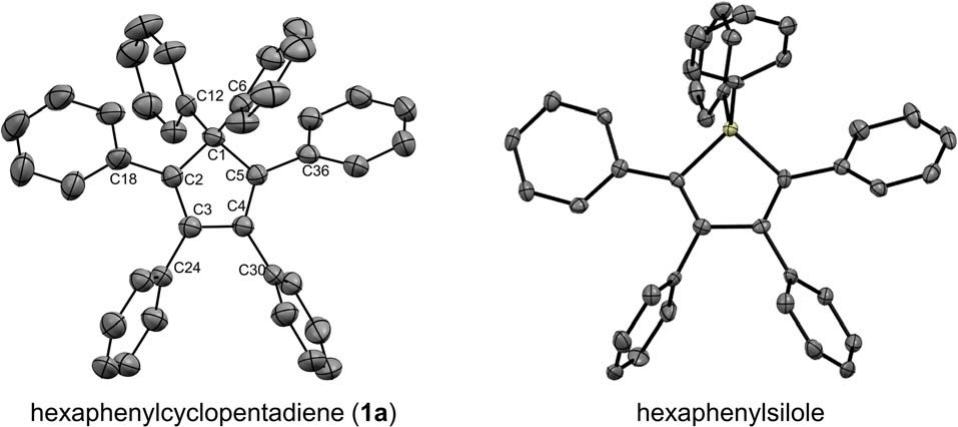
ORTEP top view of the molecular structure of hexaphenylcyclopentadiene 1a (left) and hexaphenylsilole^24^ (right). Thermal ellipsoids are drawn at 50% probability level. Hydrogen atoms and solvent molecules (heptane, in the case of 1a) are omitted for clarity.

Under optimised conditions, tetraphenylcyclopentadiene 3a was reacted with iodobenzene (6 equiv.) in THF (0.25 M) in the presence of cesium carbonate (3 equiv.) as base and copper(i) iodide (10 mol%, *i.e.* 5 mol% per new C–C bond in 1a) associated to (±)-*trans*-1,2-cyclohexanediamine (20 mol%) as catalytic system. After 2 hours under microwave irradiation (200 °C max., 250 W available power), full conversion of starting material 3a was reached and hexaphenylcyclopentadiene 1a, resulting from two consecutive arylations, was obtained in 88% isolated yield (Scheme 2). An X-ray crystal structure of compound 1a was obtained (Fig. 1, left, and S15†), thus unambiguously confirming the structure of the perarylated cyclopentadiene.

During the extensive optimisation of this reaction, influence of the catalytic system, base, coupling partner, solvent, temperature and thermal activation mode were thoroughly screened. Detailed results can be found in the ESI† section (part II.1, Tables S1–S5†). Importantly, the nature of the aryl halide appeared to be crucial, as the reaction was totally inhibited when bromobenzene was employed instead of iodobenzene, in contrast to palladium-catalysed cyclopentadiene arylations reported by Dyker, Miura *et al.*^20^

The optimal conditions of the model reaction were next transposed to the perarylation of bare cyclopentadiene, expecting the formation of six new C–C bonds in a single synthetic operation instead of two previously. To that end, the amounts of catalyst, base and coupling partner were adjusted. Using freshly distilled cyclopentadiene in the presence of 12 equivalents of iodobenzene, the desired hexaphenylcyclopentadiene 1a was obtained in 46% yield, along with 22% of pentaphenylcyclopentadiene 2a (Table 1, entry 1).

**Table 1:** Variation of the cyclopentadiene source in the copper-catalysed direct perarylation

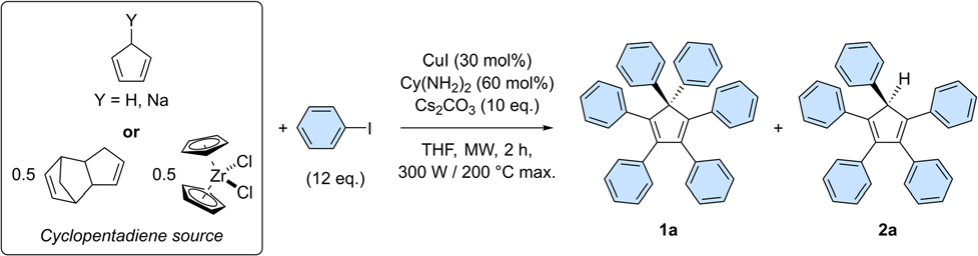				
Entry	Cyclopentadiene source	1a^a^ (%)	2a^a^ (%)	1a + 2a^a^ (%)
1^b^	Cyclopentadiene^c^^,^^d^	46 (*88*)^e^	22	68
2^b^	[Cyclopentadiene^d^ + NaH]^c^	61 (*92*)^e^	19	80
3^f^	CpNa^c^	54 (*90*)^e^	0	54
4^b^	Dicyclopentadiene^g^	23 (*78*)^e^	25	48
5	ZrCp_2_Cl_2_^g^	54 (*90*)^e^	0	54
6^h^	ZrCp_2_Cl_2_^g^	61 (*92*)^e^	0	61
7^i^	ZrCp_2_Cl_2_^g^	27 (*80*)^e^	26	53

a Compounds 1a and 2a were isolated as a mixture by column chromatography and the ratio of the two species was estimated by ^1^H NMR spectroscopy. In cases when 2a was not detected, pure product 1a was directly isolated by column chromatography.

b Reaction time was increased to 2.5 h.

c 1 equivalent.

d Freshly distilled cyclopentadiene was used.

e The bracketed number in italics represents the average yield per newly-formed C–C bond in product 1a.

f 10 equivalents of iodobenzene were used.

g 0.5 equivalent.

h The loading of CuI was reduced to 3 mol%.

i The loading of CuI was reduced to 0.3 mol%.

The efficiency of the perarylation reaction could be substantially improved up to 61% by adding sodium hydride (1 equivalent) to the reaction mixture, in order to initially generate *in situ* the cyclopentadienide anion, unreactive towards Diels–Alder cycloaddition reactions (entry 2). The same trend was observed when employing preformed solid sodium cyclopentadienide, which in addition proved selective towards perarylated product 1a. At this point, it is important to underline that yield variations are significantly amplified by the sequence of six successive cross-couplings, since the 46–61% amplitude in these perarylation reactions corresponds to a narrow range of 88–92% for the average yield per newly-formed C–C bond.

**Scheme 3:**
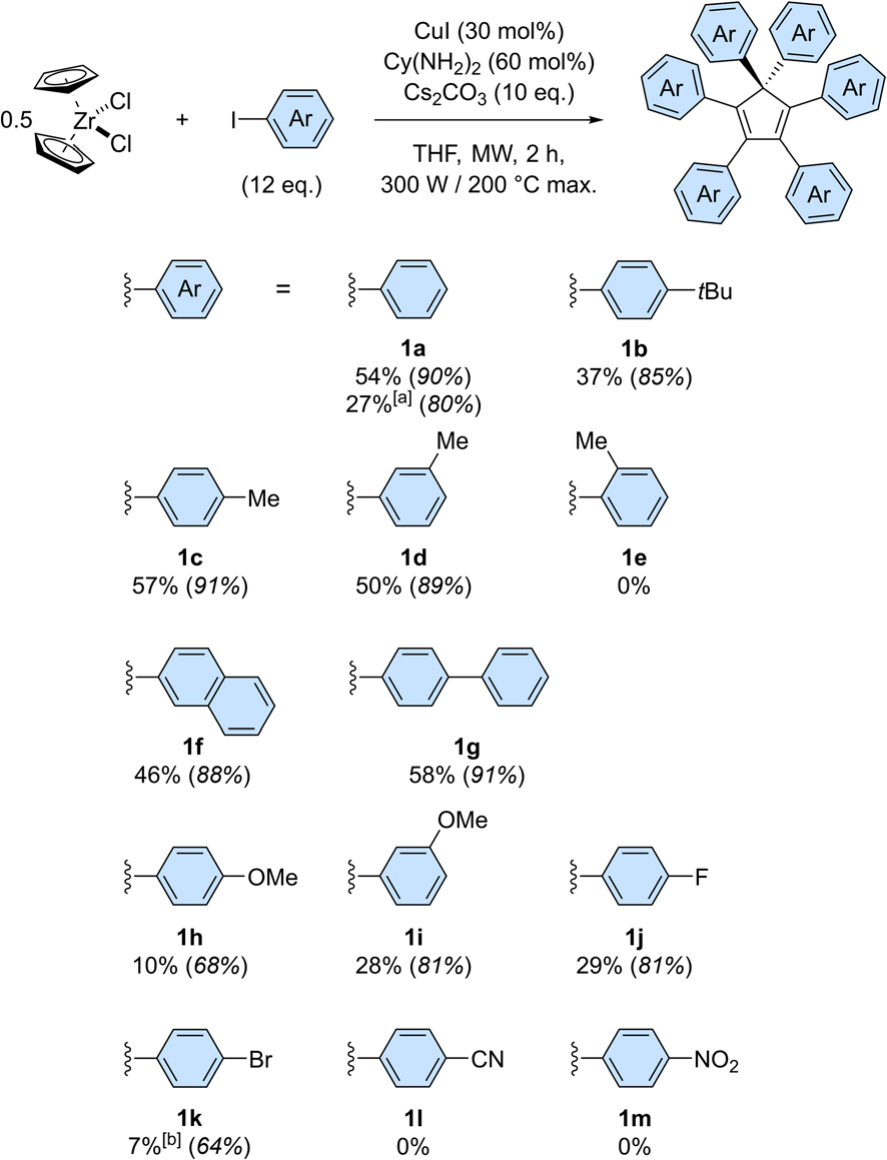
Scope of the copper-catalysed direct perarylation of cyclopentadiene, using zirconocene dichloride as a cyclopentadienide source. Isolated yields are given as an average value of at least two experiments and the bracketed numbers in italics represent the corresponding average yield per newly-formed C–C bond. Reactions were performed on a 0.048 mmol scale, *i.e.* 0.096 mmol of cyclopentadienide, unless otherwise stated. ^[a]^ The scale was increased to 0.96 mmol of ZrCp_2_Cl_2_ (*i.e.* 1.92 mmol of cyclopentadienide). ^[b]^ Yield includes partially debrominated compounds.

For practical reasons and given the high temperature conditions of the reaction, it was next envisioned to exploit dicyclopentadiene as a source of two cyclopentadiene units, generated *in situ via* retro Diels–Alder reaction (entry 4). This concept was validated by the obtention of hexa- and pentaarylated products, albeit in moderate yields that limit the synthetic use of the process. Finally, as initially suggested by Dyker, Miura *et al.*,^20^ zirconocene dichloride was employed as a source of two cyclopentadiene units. This stable and easy to handle substrate led selectively to the desired perarylated product 1a in 54% yield (entry 5). The catalyst loading was then reduced by a factor 10 to reach 3 mol% (*i.e*. 0.5 mol% per single cross-coupling), leading to hexaphenylcyclopentadiene 1a in 61% yield, equivalent to 92% average yield per newly formed C–C bond (entry 6). A further reduction of the catalyst loading to 0.3 mol% (*i.e.* 500 ppm per single cross-coupling) remains productive but the yield of the sixth arylation is noticeably decreased, leading after 2 h to 27% of the perarylated product 1a, along with 26% of the pentaphenylcyclopentadiene intermediate 2a (entry 7 and Table S6†). Finally, the reaction was scaled up by a factor 20 and was run on 0.96 mmol of zirconocene dichloride, *i.e.* 1.92 mmol of cyclopentadiene equivalent (Scheme 3 and Table S8†). Some reaction parameters were adjusted, such as the medium concentration and the available microwave power to afford, after 2 h, the desired hexaphenylcyclopentadiene 1a in 27% isolated yield (267 mg).

The scope of the copper-catalysed perarylation reaction was next examined, using the very convenient zirconocene dichloride as a source of two cyclopentadiene units. A variety of aryl iodides were screened (Scheme 3), revealing that the 6-fold arylation process is sensitive to electronic effects and has a moderate tolerance towards steric hindrance of the coupling partner. Indeed, in the presence of electron donating substituents, hexaarylation of cyclopentadiene took place with modest to good efficiency, with average yields per new C–C bond in the 85–91% range for alkyl substituents (1b–1d) and in the 68–81% range for the stronger methoxy donor (1h, 1i). In contrast, strong electron withdrawing groups such as nitrile and nitro moieties failed to deliver any hexaarylated product (1l, 1m). It is important to note here that the absence of hexaarylcyclopentadiene under such conditions does not imply that the copper-catalysed direct arylation completely fails with electron-poor coupling partners: the initial 1- to 5-fold C–C couplings may still occur, while the sixth arylation is prevented.

In this reaction, halogen substituents are tolerated, with 1-fluoro-4-iodobenzene leading to hexa(4-fluorophenyl)cyclopentadiene 1j in 29% overall yield. Importantly, competitive debromination was encountered when 1-bromo-4-iodobenzene was used, thus showing that aryl bromides are reactive under such conditions even though they are not efficient as coupling partners (Table S1,† entry 4). The copper-catalysed perarylation of cyclopentadiene also proved compatible with more complex aryl iodides such as 4-iodobiphenyl and 2-iodonaphthalene, which gave in 46–58% overall yield the corresponding hexaarylcyclopentadienes 1g and 1f, respectively. The latter, incorporating six naphth-2-yl substituents, is particularly sterically crowded but still undergoes free rotation at room temperature, as concluded from the ^1^H NMR spectrum. Further increase of steric hindrance was tested using 1-iodonaphthalene, which resulted in a severe drop of efficiency. A similar study was carried out with iodotoluene positional isomers: *para* and *meta* substitution resulted in comparable efficiency (57% yield for 1c and 50% for 1d) whereas further increase of steric hindrance with *ortho* substitution fully prevented the sixth arylation, as shown by the selective formation of penta(2-methylphenyl)cyclopentadiene in 32% yield.

Based on reported experimental and computational mechanistic studies related to the Hurtley reaction^22*c*,25^ and more generally to Ullmann-type couplings,^26,27^ we hypothesise that Cu(i) is the active catalytic species in the copper-mediated arylation of cyclopentadiene and that the catalytic cycle may proceed as follows (Scheme S2†). In the first step, the active LCu(i)I catalytic species may undergo a displacement of the iodide anion by the cyclopentadienide R_5_Cp^−^ (R=H or Ar) present in the basic reaction medium to yield the corresponding LCu(CpR_5_) complex (L = ligand). Next, oxidative addition of the iodoarene coupling partner may take place to afford a Cu(iii) intermediate, and the subsequent reductive elimination would lead to C–C bond formation with the release of the arylated cyclopentadiene product and of the catalytically-active copper(i) species. Nevertheless, according to literature reports, mechanistic pathways in Hurtley and Ullmann-type reactions appear to be closely related to the substrates, ligands and reaction conditions.^22*c*,25,26^ Therefore, after the widely-accepted initial formation of the LCu(CpR_5_) species by nucleophilic displacement, alternative mechanistic evolutions cannot be ruled out, such as a single-electron transfer (SET), an iodine atom transfer (IAT) or a four-centre σ-bond metathesis (Scheme S3†).

Next, the structural properties of hexaarylcyclopentadienes were investigated and X-ray crystal structures of target compounds 1a (Fig. 1, left, and S15†), 1b–d and 1h (see the ESI† section, part VII) were obtained. They all share the same characteristics and the structure of the cyclopentadiene ring in such hexaarylated compounds is essentially the same as in pentaphenylcyclopentadiene 2a.^28^ As expected, the cyclopentadienyl ring is planar and in 1a the length of the endocyclic bonds involving the sp^3^ carbon C1 is 1.53 Å (C1–C2 and C1–C5), thus longer than the bonds involved in the buta-1,3-diene pattern (C2–C3 and C4–C5: 1.35 Å; C3–C4: 1.50 Å). The endocyclic bond angle at the sp^3^ carbon C1 (C2–C1–C5) is 102° whereas all other angles within the cycle are in the 109°–110° range. As anticipated, hexaphenylcyclopentadiene adopts a propeller-shaped geometry with the four phenyl rings located on positions C2–C5 twisted with respect to the cyclopentadiene plane (44°–60° torsion angles). Finally, the steric hindrance caused by the remaining geminal phenyl groups on the sp^3^ carbon C1 is accommodated by a distortion of the tetrahedral geometry with a widening of the exocyclic C6–C1–C12 angle up to 116°.

Structural comparison between hexaphenylcyclopentadiene 1a and hexaphenylsilole^24^ (Fig. 1, right) highlights a distortion of the geometry caused by the silicon atom, in particular for the planar silacyclopentadiene ring, due to expectedly longer Si–C bonds (1.86–1.87 Å) compared to the corresponding C–C bonds in 1a.

**Fig. 2:**
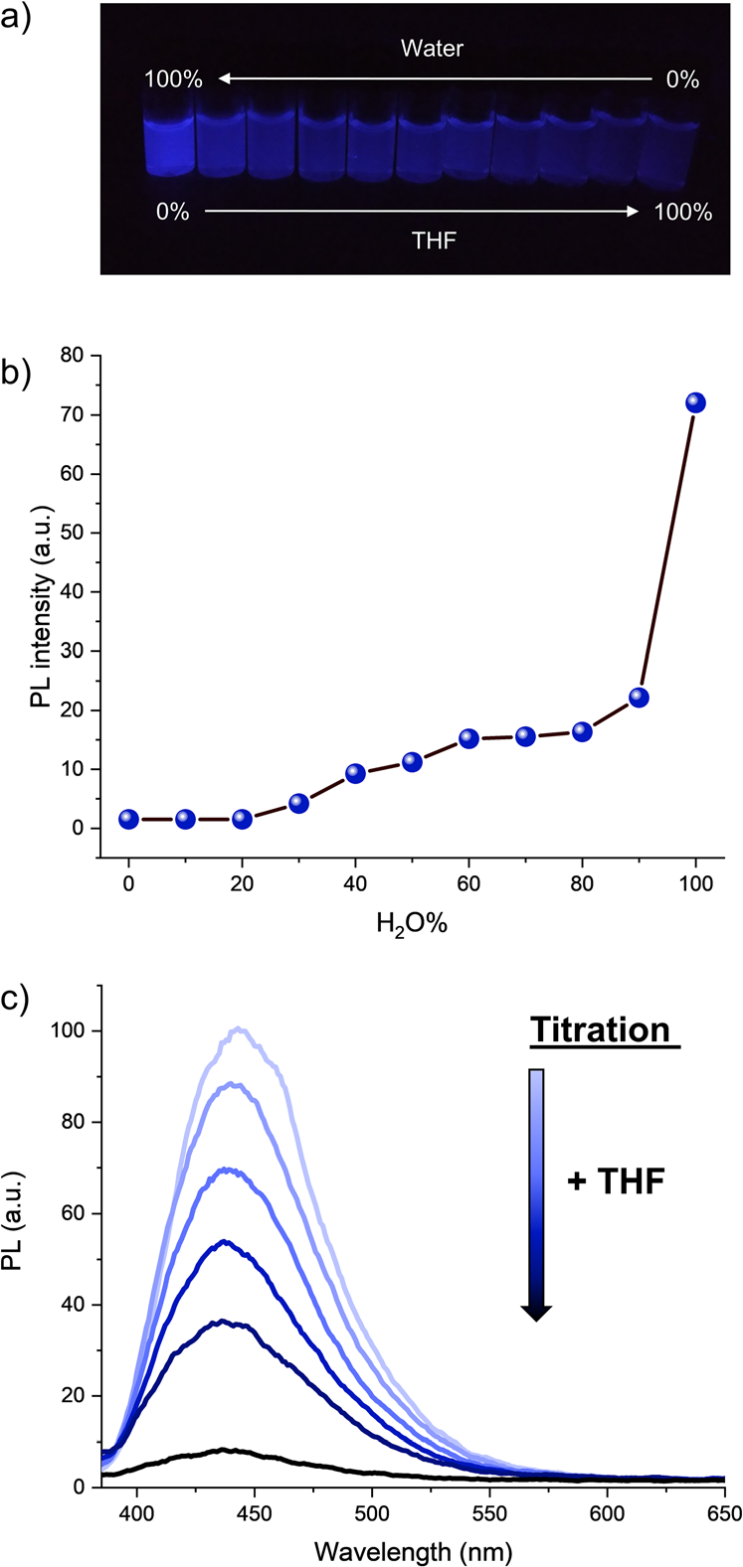
Aggregation-induced emission properties of hexaphenylcyclopentadiene 1a. (a) Picture of 1a solutions with varying H_2_O/THF ratios. (b) Data plot representation of photoluminescence intensity of 1a at *λ*_max_ = 443 nm (*λ*_Ex_ = 350 nm) *vs.* H_2_O fraction in the THF/H_2_O mixture. (c) Photoluminescence spectrum of 1a in pure H_2_O (light blue curve), 10^−7^ M, *λ*_Ex_ = 350 nm, and photoluminescence spectra resulting from the successive additions of small aliquots (20 μL) of THF (darker curves).

The optical properties of hexaphenylcyclopentadiene 1a were subsequently studied. Its absorption spectrum in THF solution displays two maxima at 247 and 340 nm (Fig. S2†), which is similar to the absorption spectrum of pentaphenylcyclopentadiene 2a.^11*e*^ It is however blue-shifted in comparison with hexaphenylsilole (*λ*_max_ = 250, 365 nm).^29^ As expected from the numerous degrees of freedom resulting in free rotation of aryl groups at room temperature in solution (as observed by NMR spectroscopy), hexaphenylcyclopentadiene 1a is virtually nonemissive in dilute THF solution (*ca.* 10^−7^ M). Upon addition of increasing fractions of a miscible non-solvent such as water (in which compound 1a is not soluble), the photoluminescence intensity increases gradually and reaches its maximum in pure water, with the maximum emission wavelength located at 443 nm (Fig. 2a, b and S3†). From Fig. 2b, it can be clearly seen that a large fraction of water (>90%) is required to trigger intense photoluminescence. Conversely, from the aggregate state in pure water, the rotation of phenyl substituents can be activated again by simple titration of the sample with THF. As depicted in Fig. 2c, the addition of small aliquots of THF resulted in the exponential drop of the photoluminescence intensity owing to molecular solvation and subsequent disaggregation. In agreement with the behaviour of propeller-shaped polyarylcyclopentadienes Ar_*n*_H_(6−*n*)_Cp (*n* = 3–5),^11^ hexaphenylcyclopentadiene 1a thus displays AIE and appears as a promising candidate as blue-emitter for optoelectronic applications.

The electron-enriched hexa(4-methoxyphenyl)cyclopentadiene 1h also proved to be AIE active (Fig. S4–S6†) and most importantly, its emission peak is red-shifted by 10 nm compared to 1a. This underlines the possibility to tailor the optoelectronic properties of hexaarylcyclopentadiene AIE luminogens, and the synthetic method developed above thus appears as a powerful tool to access a large variety of unprecedented hexaarylcyclopentadienes with fine-tuned properties.

**Scheme 4:**
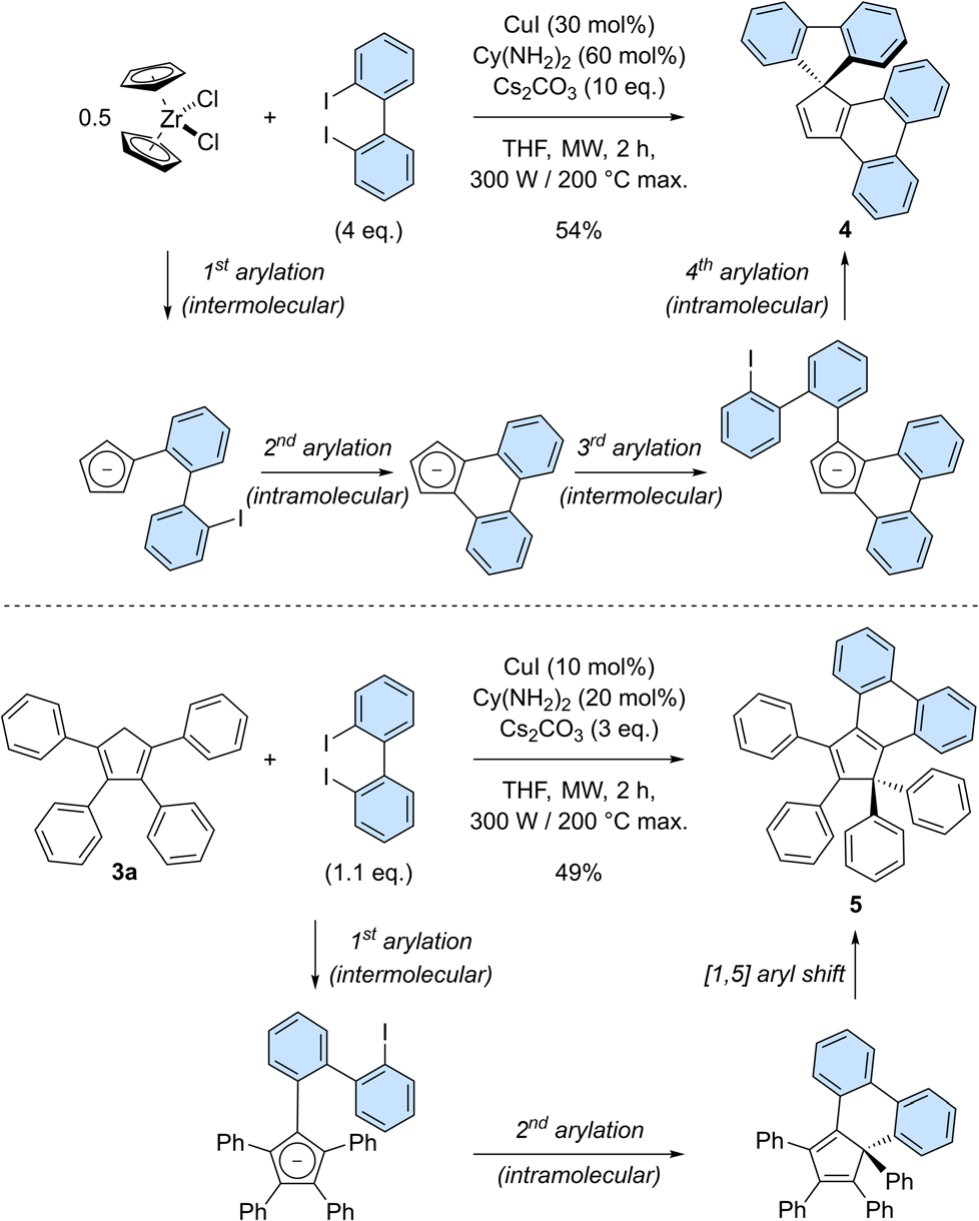
Copper-catalysed direct arylation of zirconocene dichloride (top) and tetraphenylcyclopentadiene 3a (bottom) with 2,2′-diiodobiphenyl as coupling partner, yielding cyclopenta[*l*]phenanthrenes. The reaction intermediates resulting from the successive C–C bond-forming steps are depicted.

Having established the robustness of the copper-catalysed perarylation reaction and the promising optical properties of hexaarylcyclopentadienes, we next aimed at increasing the structural complexity of the products obtained in this process. It was thus envisioned to exploit 2,2′-diiodobiphenyl as bifunctional coupling partner allowing for the formation of polycyclic cyclopentadiene derivatives (Scheme 4). Reaction of zirconocene dichloride with 2,2′-diiodobiphenyl (4 equivalents) under copper-catalysed conditions gave rise to spirofluorene 4 in 54% yield (Scheme 4, top). This product formally results from the coupling of cyclopentadiene with two 2,2′-biphenylenes, with the formation of four new C–C bonds in a single synthetic operation. Successive inter- and intramolecular arylation reactions occur first on adjacent positions of the cyclopentadiene to generate an intermediate cyclopenta[*l*]phenanthrene (as an anion under basic reaction conditions), which subsequently undergoes a *gem*-diarylation on the most favoured position^20*b*^ thus yielding spirofluorene 4. Importantly, the corresponding cyclopenta[*l*,*l*′]diphenanthrene resulting from the coupling of three 2,2′-biphenyl moieties was not observed.

**Fig. 3:**
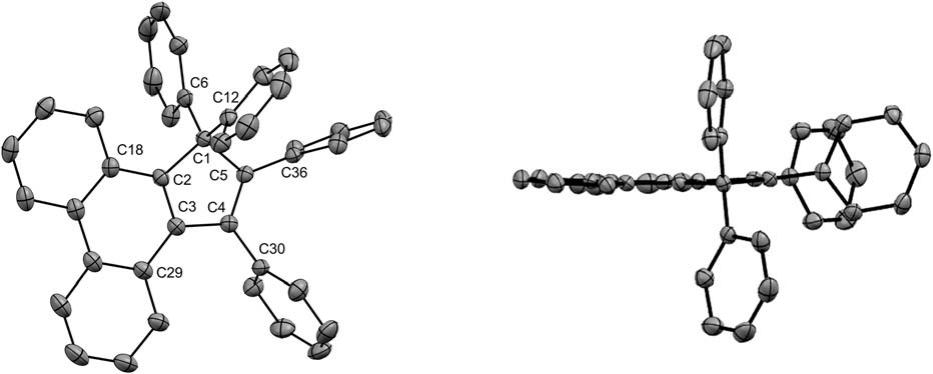
ORTEP top view (left) and side view (right) of the molecular structure of 1,1,2,3-tetraphenyl-1*H*-cyclopenta[*l*]phenanthrene 5. Thermal ellipsoids are drawn at 50% probability level and hydrogen atoms are omitted for clarity.

Given the highly promising properties of spirofluorenes for optoelectronic applications,^30^ the *gem*-diarylation of tetraphenylcyclopentadiene 3a using 1.1 equivalent of 2,2′-diiodobiphenyl was next attempted (Scheme 4, bottom). Much to our surprise, the expected spirofluorene was not formed upon spiroannulation in these conditions and 1,1,2,3-tetraphenylcyclopenta[*l*]phenanthrene 5 was isolated instead. Its structure was unambiguously confirmed by X-ray diffraction analysis of a crystal obtained upon train sublimation (Fig. 3), thus highlighting the fully planar character of the cyclopenta[*l*]phenanthrene moiety decorated with twisted phenyl groups. As such, compound 5 can be viewed as an analogue of propeller-shaped hexaphenylcyclopentadiene 1a displaying an extended π-conjugated core for enhanced emission properties in the aggregate state^31^ and potential application in OLEDs.

The *gem*-diphenyl pattern found in cyclopenta[*l*]phenanthrene 5 implies that migration of a phenyl group occurred during the reaction, which is consistent with reports on similar systems^32^ and which was in particular exploited as key step in the only synthesis of hexaphenylcyclopentadiene reported to date.^13^ In this reaction, we thus hypothesise that the copper-catalysed diarylation takes place on adjacent positions of the cyclopentadiene, leading to a 6-membered ring instead of the more strained 5-membered ring required in the desired spirofluorene. Next, a thermally-activated [1,5] sigmatropic shift of the phenyl group located on the sp^3^ carbon takes place, yielding a planarised cyclopenta[*l*]phenanthrene fragment.

**Scheme 5:**
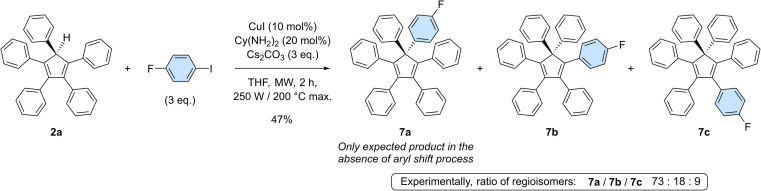
Copper-catalysed monoarylation of pentaphenylcyclopentadiene 2a with 1-fluoro-4-iodobenzene, leading to the corresponding hexaarylated product 7 as a 73 : 18 : 9 mixture of regioisomers.

To get deeper insight into the occurrence of such aryl migrations during copper-catalysed arylation of cyclopentadiene with monoiodoarenes as coupling partners, pentaphenylcyclopentadiene 2a was reacted with 1-fluoro-4-iodobenzene under optimised coupling conditions (Scheme 5). In the absence of rearrangement, only 7a should be obtained, carrying both a phenyl and a 4-fluorophenyl moiety on the sp^3^ carbon of the cyclopentadiene core. Experimentally, a mixture of perarylated regioisomers was evidenced by ^19^F NMR in a 73 : 18 : 9 ratio and their structure was further confirmed by the X-ray diffraction analysis of a single co-crystal of the three isomers (see the ESI section, page S96†). Due to the occurrence of [1,5] sigmatropic shifts in cyclopentadiene derivatives, and in particular in hexaarylcyclopentadienes, synthetic methods relying on transition-metal catalysed arylation thus appear to be restricted to the preparation of identically-substituted polyarylated cyclopentadienes. Even with this limitation, the one-step synthesis of AIE-active hexaarylcyclopentadienes upon copper catalysis remains a powerful tool with promising applications in optoelectronics.

**Scheme 6:**
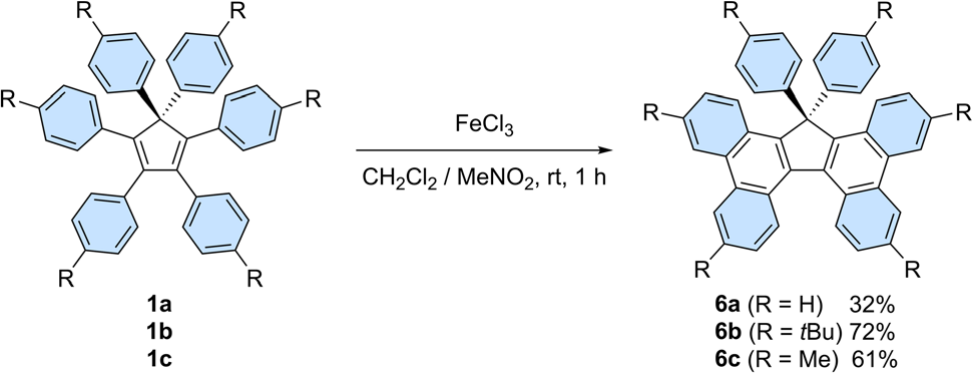
Synthesis of 17,17-diarylcyclopenta[*l*,*l*′]diphenanthrenes 6a–c from hexaarylcyclopentadienes 1a–c*via* a Scholl reaction.

In addition, on top of their intrinsic properties, propeller-shaped compounds 1 can also be viewed as direct precursors of highly attractive π-extended scaffolds for organic electronics, such as 17,17-diarylcyclopenta[*l*,*l*′]diphenanthrenes (also referred to as 17,17-diaryltetrabenzo[*a*,*c*,*g*,*i*]fluorenes, Scheme 6) and the corresponding spirobifluorenes.^30*b*,33^ In this context, Stuparu *et al.* very recently disclosed the successful conversion of pentaphenylcyclopentadiene 2a into 17-phenyl-tetrabenzo[*a*,*c*,*g*,*i*]fluorene upon mechanochemical Scholl reaction,^34^ thus improving initial attempts by Dyker *et al.*^35^

Hexaphenylcyclopentadiene 1a and its *tert*-butyl and methyl *para*-substituted counterparts 1b and 1c, respectively, were next submitted to a Scholl reaction,^36^ with the aim to trigger the formation of π-extended scaffolds. In spite of the wide set of conditions tested, spirobifluorenes were never detected, which can be accounted for by the high strain associated with the formation of the second fluorene pattern upon 5-membered ring closure. Conversely, the planarised 17,17-diarylcyclopenta[*l*,*l*′]diphenanthrenes 6b and 6c, resulting from the formation of two new C–C bonds, were obtained in 72 and 61% yield, respectively, upon treatment with iron(iii) chloride (Scheme 6). The non-substituted counterpart 6a was isolated in lower yield (32%) due to partial chlorination of the phenyl *para*-positions, as observed by mass spectrometry analysis of the crude mixture.

**Fig. 4:**
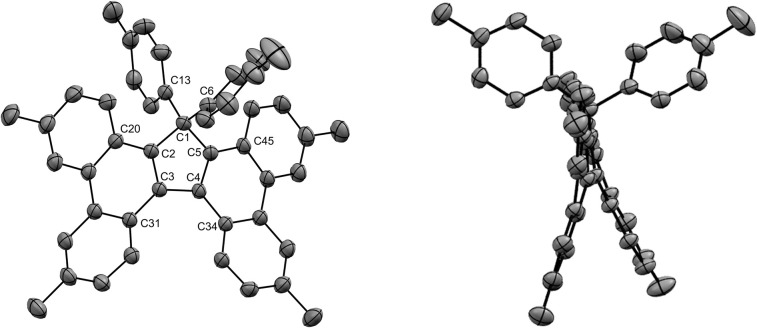
ORTEP top view (left) and side view (right) of the molecular structure of cyclopenta[*l*,*l*′]diphenanthrene 6c. Thermal ellipsoids are drawn at 50% probability level and hydrogen atoms are omitted for clarity.

The structures of tetrabenzofluorenes 6a–c were assigned according to ^1^H and ^13^C NMR spectra, with the characteristic resonance of the cyclopentadiene sp^3^ carbon at *δ* = 68–69 ppm. As expected, the protons located in the bay and fjord regions are particularly deshielded (8.53 < *δ* < 8.80 ppm) and the 17,17-diaryl substitution pattern is evidenced by the characteristic AA’BB’ systems corresponding to the two *para*-substituted phenyl groups in compounds 6b and 6c. For the latter, crystals suitable for X-ray diffraction analysis were obtained upon slow evaporation of dichloromethane/methanol solutions, thus unambiguously confirming the obtained structures (Fig. 4, S26 and S28†). Similarly to hexaarylcyclopentadienes 1b-c, the steric hindrance caused by the geminal aryl groups on sp^3^ carbon C1 is accommodated by a distortion of the tetrahedral geometry, with a widening of the exocyclic C6–C1–C16 angle up to 117° in compound 6b (Fig. S26†). As expected from the *ortho*-annulation of the five rings in the fjord region,^37^ the tetrabenzofluorene scaffolds display a distorted structure with an helical geometry. In compound 6c, the torsion angle C31–C3–C4–C34 is worth 28°, and this angle is reduced to 23° in fluoreno[5]helicene 6b incorporating *tert*-butyl instead of methyl *para*-substituents. These values are in line with those reported in literature for 17-monoaryl-^34^ and 17,17-dialkyltetrabenzofluorenes,^38^ and the variation of amplitude may be ascribed to the packing force in the crystal. In any case, the racemisation barrier for such helicenic species is low and both enantiomers cannot be separated at room temperature.

The obtained 17,17-diarylcyclopenta[*l*,*l*′]diphenanthrenes 6a–c can be viewed as extended 9,9-diarylfluorenes, one of the most significant classes of organic semiconductors and a key building unit for the design of fluorophores.^39^ As expected, upon cyclodehydrogenation of precursors 1a–c and concomitant planarisation of the structures, the absorption spectra undergo significant changes (Fig. S7–S9†). For tetrabenzofluorenes 6a–c, a bathochromic shift related to the extension of their π-conjugated scaffold is observed, with two new bands appearing at 371–378 nm and 389–397 nm, respectively, in comparison with the spectra of hexaarylcyclopentadienes 1a–c. Moreover, the increased structural rigidity in cyclopenta[*l*,*l*′]diphenanthrenes 6a–c is revealed by the structuration of their absorption spectra, and by the restoration of photoluminescence in solution. The emission spectra in dichloromethane solutions exhibit a mirror symmetry to the lowest energy absorption bands, with two distinct emission bands located at 410–416 nm and 425–436 nm, respectively (Schemes S10–S12†).

## Conclusions

In this work, the direct perarylation of cyclopentadiene was achieved upon copper(i) catalysis and microwave activation to give rise to hexaphenylcyclopentadiene, an isostructural hydrocarbon surrogate of the archetypal AIE luminogen hexaphenylsilole. Using zirconocene dichloride as a convenient source of cyclopentadiene and a variety of aryl iodides as coupling partners, a series of unprecedented hexaarylcyclopentadienes was synthesised according to this straightforward method allowing the formation of six new C–C bonds in a single synthetic operation.

The structural and optical properties of hexaarylcyclopentadienes were investigated, with a focus on their AIE behaviour. As expected from their propeller-shaped structure, these compounds are virtually nonemissive in solution but intense photoluminescence is observed upon aggregation, with an emission spectrum depending on the nature of the aryl substituents. Hexaarylcyclopentadienes thus appear as promising AIE luminogens for the fabrication of high performance optoelectronic devices, and the Cu-catalysed perarylation reaction offers a direct access to a large variety of derivatives displaying fine-tuned structural and electronic properties.

Hexaarylcyclopentadienes were next exploited as direct precursors of extended π-conjugated polycyclic compounds to be used as organic semiconductors or fluorophores. Upon Scholl reaction conditions, the propeller-shaped scaffolds underwent planarisation with the formation of two new C–C bonds, thus yielding 17,17-diarylcyclopenta[*l*,*l*′]diphenanthrenes. These structurally complex polyannelated fluorene derivatives displaying an helicenic structure can now be prepared in only two synthetic steps from cyclopentadiene.

## Experimental section

### Procedure for the Cu-catalysed perarylation of cyclopentadiene, using zirconocene dichloride as substrate

In a glovebox, zirconocene dichloride (14 mg, 0.048 mmol, 0.5 eq.), cesium carbonate (312 mg, 0.96 mmol, 10 eq.), (±)-*trans*-1,2-cyclohexanediamine (7 μL, 0.06 mmol, 60 mol%), copper(i) iodide (5.5 mg, 0.03 mmol, 30 mol%), anhydrous degassed THF (1 mL) and a magnetic stir bar were placed in a 10 mL tube designed for microwave irradiation. The appropriate aryl iodide (1.15 mmol, 12.0 eq.) was added and the suspension was briefly shaken before sealing the vial. The mixture was then heated using microwave irradiation at 200 °C for two hours, setting up the microwave with an available power of 300 W and a maximal pressure of 20 bars (typically, the pressure stabilised between 10 and 15 bars depending on the conditions). After cooling down the reaction medium and carefully releasing the pressure, the reaction mixture was diluted with CH_2_Cl_2_ (10 mL) and filtered over a short Celite plug (eluted with CH_2_Cl_2_). The solvents were removed and the crude product was dissolved in CH_2_Cl_2_, adsorbed onto silica and purified by column chromatography on silica gel to isolate the desired hexaarylcyclopentadiene product.

## Data availability

The data that support the findings of this study are available in the ESI† section. Crystallographic data for compounds 1a–1d, 1h, 5, 6b, 6c and 7 have been deposited at the The Cambridge Crystallographic Data Centre under CCDC-2214598 to CCDC-2214606. These data can be obtained from the CCDC *via*https://www.ccdc.cam.ac.uk/structures.

## Author contributions

Y. G. conceived the project, optimised the reaction, assessed the scope and performed mechanistic investigations. P. S. M. achieved the AIE studies. C. B. synthesised the tetrabenzofluorene derivatives *via* Scholl reaction and performed subsequent characterisation. S. A. contributed to the substrate scope investigation and to supervision. N. S.-M. performed the single-crystal X-ray analyses. G. R. and C. K. supervised the study. All authors discussed the results and contributed to the final manuscript.

## Conflicts of interest

There are no conflicts to declare.

## Supplementary Material

SC-015-D4SC02458C-s001

SC-015-D4SC02458C-s002
