# Supplementary material for: Copper-catalysed perarylation of cyclopentadiene: synthesis of hexaarylcyclopentadienes
Source: Chem Sci. 2024 May 16;15(24):9127–37. doi: 10.1039/d4sc02458c (PMC11186316; doi:10.1039/d4sc02458c)
Supplement: SC-015-D4SC02458C-s001 [file SC-015-D4SC02458C-s001.pdf]

Electronic Supplementary Material (ESI) for Chemical Science.
This journal is © The Royal Society of Chemistry 2024

Supporting Information

Copper-Catalysed Perarylation of Cyclopentadiene: Synthesis of Hexaarylcyclopentadienes

Yohan Gisbert,[a] Pablo Simón Marqués,[a] Caterina Baccini,[a] Seifallah Abid,[a] Nathalie Saffon-Merceron,[b] Gwénaël Rapenne [a,c] and Claire Kammerer *[a]

[a] *CEMES, Université de Toulouse, CNRS, 29 rue Marvig, 31055 Toulouse, France*
[b] *Université de Toulouse, UPS, Institut de Chimie de Toulouse, ICT UAR 2599, 118 route de Narbonne, 31062 Toulouse, France*
[c] *Division of Materials Science, Nara Institute of Science and Technology, 8916-5 Takayama, Ikoma, Nara, Japan*

Table of contents

I. Materials and methods……… S2

II. Detailed results of the optimisation of the copper-catalysed hexaarylation of cyclopentadiene……… S4

II.1. Optimisation of the copper-catalysed arylation of tetraphenylcyclopentadiene……… S4

II.2. Optimisation of the copper-catalysed perarylation of cyclopentadiene……… S10

II.3. Mechanistic considerations……… S13

III. Experimental procedures and characterisation……… S14

IV. NMR spectra of new compounds……… S26

V. Absorption and emission properties……… S74

V.1. Absorption spectra of compounds **1a** and **1h**……… S74

V.2. Aggregation-induced emission of compounds **1a** and **1h**……… S75

V.3. Absorption and emission spectra of compounds **6a-c**……… S77

VI. Cyclic voltammetry……… S79

VII. Crystallographic data……… S80

VIII. References……… S98

I. Materials and methods

Commercial reagents and solvents: All chemicals and solvents were purchased from commercial suppliers unless otherwise stated and were purified using standard laboratory techniques if needed, such as distillation, drying with 4 Å molecular sieves or filtration over a relevant stationary phase.
1,10-Phenanthroline and zirconocene dichloride were purchased from Acros Organics. 1-Iodo-4-nitrobenzene, 2-iodonaphthalene, 1-bromo-4-iodobenzene, cesium carbonate, copper(I) iodide, copper(I) thiophene-2-carboxylate (CuTC), dicyclopentadiene, diisopropylamine, lithium *tert*-butoxide, palladium(II) acetate, potassium carbonate, (±)-proline, sodium carbonate, sodium cyclopentadienide (2.4 M in THF), sodium hydride, sodium *tert*-butoxide, (±)-*trans*-1,2-cyclohexanediamine and (±)-*trans-N,N'*-dimethyl-1,2-cyclohexanediamine were purchased from Aldrich. 2-Iodotoluene, 3-iodotoluene, 4-iodobenzonitrile and iodobenzene were purchased from Fluorochem. 1-Fluoro-4-iodobenzene, 4-iodoanisole and 4-iodotoluene were purchased from TCI chemicals. 4-Iodobiphenyl was purchased from ABCR. 2,2'-Bipyridine and copper(I) bromide were purchased from Alfa Aesar. 1-*tert*-Butyl-4-iodobenzene was purchased from Apollo Chemicals.
Anhydrous solvents were all purchased from Aldrich. Deuterated solvents used for analysis were purchased from Eurisotop.
For reactions involving cyclopentadiene, dicyclopentadiene was freshly cracked and quickly degassed by three successive freeze-pump-thaw cycles and used immediately.
For reactions involving sodium cyclopentadienide, it was purchased from Aldrich as a 2.4 M solution in THF and was dried on a Schlenk line before being transferred to a glovebox where it was stored and used as a solid to prevent inaccuracy related to concentration variations. This solid is highly reactive and should be handled with care (see "Safety notes" below).

Synthesised reagents: 1,2,3,4-Tetraphenylcyclopenta-1,3-diene (**3a**)[1] and 2,2'-diiodobiphenyl[2] were synthesised according to reported literature procedures and characterised using routine characterisation techniques.

Synthesis and purification: Reactions were carried out using standard Schlenk techniques under argon atmosphere or using a glovebox under argon ([H_2O] and [O_2] < 2 ppm.). Microwave reactions were carried out using CEM Discover LabMate with 10 mL tubes, equipped with an ovoid 1 cm long PTFE-coated magnetic stir bar, sealed with clippable PTFE caps. Scaled up microwave reactions were performed using a CEM Mars 6 microwave and 100 mL PTFE EasyPrep Plus reactors with a fiber optic temperature sensor. Column chromatography was carried out on 230–400 mesh silica gel (Aldrich). Celite 545 was purchased from Merck. Thin layer chromatography (TLC) was performed on pre-coated aluminum-backed silica gel 60 UV254 plates (Macherey–Nagel or Merck) with visualisation effected using ultraviolet irradiation (λ = 254, 366 nm).

Analysis: NMR spectroscopy, mass spectrometry, X-ray diffraction and elemental analysis were performed by the appropriate services of the Toulouse Institute of Chemistry (ICT – UAR 2599).

^{1}H, ^{13}C and ^{19}F-NMR spectra were recorded on Bruker Avance III HD 500 MHz (cryoprobe Prodigy 5mm BBO, 1H ATMA) and Avance 500 MHz (cryoprobe 5mm 1H, 13C) spectrometers. $^{19}F\{1H\}$ were recorded on a Bruker Avance II 400MHz (TXO probe C, H, F). Residual solvent signals were used as internal reference for ^{1}H and ^{13}C NMR. ^{19}F NMR spectra were referenced according to the solvent. Chemical shifts (δ) are reported in ppm. Coupling constants (J) are given in Hz and the following abbreviations have been used to describe the signals: singlet (s); broad singlet (br. s); doublet (d); triplet (t); virtual triplet (virt. t.); quadruplet (q); quintuplet (quint); multiplet (m). Full assignments of ^{1}H and ^{13}C NMR spectra were made with the assistance of COSY, HMBC, HSQC and NOESY spectra.
High-resolution mass spectra (HR-MS) were performed with a Waters GCT Premier spectrometer for desorption chemical ionisation (DCI-CH_4) and with a Waters Xevo G2 QTof spectrometer for electrospray ionisation (ESI). Low-resolution mass spectra (LR-MS) were performed using a Thermofisher DSQ II spectrometer for desorption chemical ionisation (DCI-NH_3).
UV–Vis spectra were recorded on a Varian Cary 5000 spectrometer (sh = shoulder, ε [mol^{-1} dm^3 cm^{-1}] is reported in parentheses) and fluorescence spectra on a Hitachi F-4500 fluorescence spectrophotometer.
Melting points were measured with a Krüss M5000 melting-point apparatus or with a Kofler hot bench and are uncorrected.
Cyclic voltammograms were obtained with an Autolab system (PGSTAT204) in anhydrous dichloromethane with 0.1 M tetrabutylammonium hexafluorophosphate $n$$Bu_4NPF_6$ as the supporting electrolyte. The measurements were carried out at 25 °C at a scan rate of 0.1 V s^{-1}. A three electrode cell was used comprising a 1 mm Pt-disk working electrode, a Pt wire auxiliary electrode, and an aqueous saturated calomel (SCE) reference electrode.

Safety notes: Sodium cyclopentadienide is a fire hazard, particularly in its dry form, and it has to be handled with care. The copper-catalysed perarylation reaction works equally well with commercial solutions, but in our case, it was dried to perform the small-scale optimisation with higher accuracy regarding the amount of reagent employed.
The copper-catalysed perarylation reaction involves the use of pressurised microwave vessels, which have to be handled with care and appropriate safety measures.

II. Detailed results of the optimisation of the copper-catalysed hexaarylation of cyclopentadiene

II.1. Optimisation of the copper-catalysed arylation of tetraphenylcyclopentadiene

Considering that the most difficult steps in the perarylation of cyclopentadiene are the fifth and sixth C-C couplings due to major steric hindrance, the copper-catalysed arylation of tetraphenylcyclopentadiene (**3a**) using iodobenzene as coupling partner was investigated as a model reaction (Scheme S1). In this case, a single arylation affords pentaphenylcyclopentadiene **2a**, which can subsequently undergo a second C-C coupling to yield the desired hexaphenylcyclopentadiene **1a**. This system thus considerably simplifies the optimisation process, since only two different arylation products may be formed, as compared with the large number of products possibly resulting from the 1- to 6-fold arylation of cyclopentadiene. Moreover, due to the bulkiness of tetra- (**3a**), penta- (**2a**) and hexaphenylcyclopentadiene (**1a**), this model reaction allows to focus on the optimisation of the copper-catalysed cross-coupling by avoiding competitive Diels-Alder [4+2] cycloaddition reactions which take place in the presence of pristine and slightly hindered cyclopentadienes.

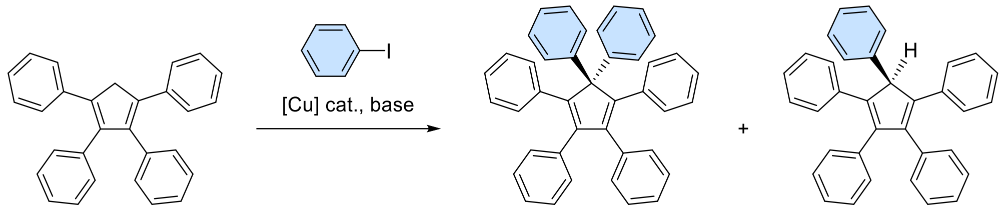

3a **1a** **2a**

Scheme S1. Model reaction for the optimisation of the copper-catalysed perarylation of cyclopentadiene.

a. Identification of optimal reaction conditions

Extensive optimisation work was carried out to establish the optimal conditions for the double arylation of tetraphenylcyclopentadiene **3a**, *i.e.* for the formation of hexaphenylcyclopentadiene **1a**. Influence of the catalytic system, base, coupling partner (amount and nature), solvent, temperature and thermal activation mode were thoroughly studied. The optimal reaction conditions are reported in Table S1 (entry 1), along with the results obtained for certain deviations from these conditions (entries 2-12).
Since arylation products **1a** and **2a** exhibit similar polarities on silica gel, they were isolated by column chromatography as a mixture, which was precisely weighed. The ratio of **1a** and **2a** was then estimated by ^{1}H NMR spectroscopy, thus allowing to report a yield for each species.

Under optimised conditions, tetraphenylcyclopentadiene **3a** was reacted with iodobenzene (6 equiv.) in THF (0.25 M) in the presence of cesium carbonate (3 equiv.) as base and copper(I) iodide (10 mol%) associated to (±)-*trans*-1,2-cyclohexanediamine (20 mol%) as catalytic system (Table S1, entry 1). After 2 hours under microwave irradiation (200 °C max., 250 W available power), full conversion of starting

material **3a** was reached and hexaphenylcyclopentadiene **1a**, resulting from two consecutive arylations, was obtained in 88% isolated yield.

Further experiments showed that the presence of oxygen is tolerated in this copper-catalysed reaction with a yield of 62% for the desired product **1a**, even though it impacts the arylation kinetics as seen from the residual amount (21%) of pentaphenylcyclopentadiene **2a** after an identical reaction time of 2 h (entry 2). A similar result was obtained when decreasing the amount of the iodobenzene coupling partner from 6 equiv. to 3 equiv. (entry 3). Importantly, the nature of the aryl halide appears crucial as the reaction was totally inhibited when bromobenzene was employed instead of iodobenzene, even upon addition of sodium iodide to mimic Buchwald's copper-mediated halogen exchange conditions.[3] This is a striking difference with the palladium-catalysed arylation of cyclopentadienes reported by Dyker *et al.*, which employs a variety of aryl bromides as coupling partners to yield the corresponding pentaarylcyclopentadienes.[4] To assess the possibility for palladium catalysts to promote the sixth arylation of cyclopentadiene, a palladium-based catalytic system similar to the one employed by Dyker *et al.*[4] was tested, and palladium(II) diacetate (10 mol%) in combination with tri(*tert*-butyl)phosphine (20 mol%) were employed instead of the copper catalyst. Under otherwise unchanged conditions, no arylation product was obtained: partial decomposition of tetraphenylcyclopentadiene **3a** was observed, leading to many undesired side products, and the starting material **3a** was partly recovered (entry 12).

We next demonstrated that thermal activation is required for this reaction to proceed, since no conversion of the starting material **3a** is observed at room temperature (entry 5). Moreover, lowering the temperature from 200 °C to 160 °C under microwave irradiation leads after 2 h to a limited isolated yield of 20% for the desired hexaphenylcyclopentadiene **1a**, along with 41% of partially arylated product **2a** (entry 6). Finally, a series of blank experiments revealed that the presence of copper iodide and base is crucial to perform the arylation of tetraphenylcyclopentadiene **3a** (entry 7). Strikingly, the monoarylation of **3a** occurs even in the absence of copper iodide, as long as a base is present (entry 9). This totally unexpected behaviour was verified multiple times by using new and thoroughly washed glassware and stir bars, with different batches of reagents. It is important to note that the second arylation leading to hexaphenylcyclopentadiene **1a** is never observed in such case, which could suggest a difference in the mechanisms involved in the arylation of tetraphenylcyclopentadiene and of pentaphenylcyclopentadiene. Omission of the ligand (entries 10 and 11) decreases the efficiency of the arylation process but the reactivity is maintained.

Detailed results about the influence of ligand, copper source, base and solvent are provided in the next sections.

Table S1. Optimal reaction conditions for the copper-catalysed arylation of tetraphenylcyclopentadiene (**3a**) and results obtained for certain deviations from these conditions.

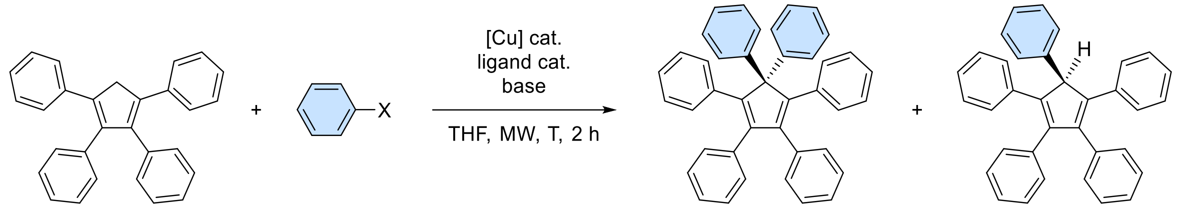

3a **1a** **2a**

Entry	Reaction conditions, deviation from the optimal	1a (%)[a]	2a (%)[a]	1a + 2a (%)[a]
1	Optimal conditions:<br>**3a** (0.5 mmol), iodobenzene (6 eq.), CuI (10 mol%), $Cy(NH_2)_2$[b] (20 mol%), Cs_2CO_3 (3 eq.), THF (2 mL), microwave heating, 250 W (max.), 200 °C (max.), 2 h, Ar	88	0	88
2	Under air, no degassing	62	21	83
3	Iodobenzene (3 eq.)[c]	67	11	78
4	Bromobenzene (3 eq.)	0	0	0
5	At RT, no microwave irradiation, iodobenzene (3 eq.)	0	0	0
6	Microwave heating at 160 °C, iodobenzene (3 eq.)	20	41	61
7	No CuI, no base	0	0	0
8	No base	0	Traces[d]	Traces[d]
9	No CuI	0	52	52
10	No CuI, no ligand	0	31	31
11	No ligand	16	49	65
12	$Pd(OAc)_2/P(tBu)_3$ as catalytic system[e]	0	0	0

[a] Compounds **1a** and **2a** were isolated as a mixture by column chromatography and the ratio of the two species was estimated by ^1H NMR spectroscopy. In cases when either **1a** or **2a** was not formed during the reaction, the pure product was then directly isolated by column chromatography. [b] $Cy(NH_2)_2$ = (±)-*trans*-1,2-cyclohexanediamine. [c] These reaction conditions were used as standard conditions in further optimisation studies. [d] Traces amounts of **2a** were observed by TLC and mass spectrometry. [e] 10 mol% $Pd(OAc)_2$ was used in combination with 20 mol% $P(tBu)_3$.

b. Influence of the ligand

As mentioned above, the arylation reaction is still operative in the absence of ligand, but the efficiency of the second arylation step is drastically reduced when the copper(I) species is not stabilised by a ligand.

In the following optimisation process, the influence of the ligand nature in the copper-catalysed arylation of tetraphenylcyclopentadiene **3a** was investigated, using the standard reaction conditions (involving 3 equivalents of iodobenzene, Table S1 entry 3) as a starting point.

Among all the ligands tested, (±)-*trans*-1,2-cyclohexanediamine gave the best results, affording hexaphenylcyclopentadiene **1a** in 67% yield along with 11% of pentaphenylcyclopentadiene **2a** (Table S2, entry 1). The use of the *N,N'*-dimethyl derivative of this ligand, (±)-*trans-N,N'*-dimethyl-1,2-cyclohexanediamine, resulted in a slightly lower efficiency (entry 2). Further bidentate ligands widely used in copper-catalysed reactions[5] such as 1,10-phenanthroline, 2,2'-bipyridine and (±)-proline were also tested, but gave modest yields in the desired product **1a** (14-32%, entries 3-5). Finally, diisopropylamine

was employed as an example of monodentate ligand but it failed to improve efficiency of the arylation process (entry 6).

Table S2. Variation of the ligand in the copper-catalysed arylation of tetraphenylcyclopentadiene.

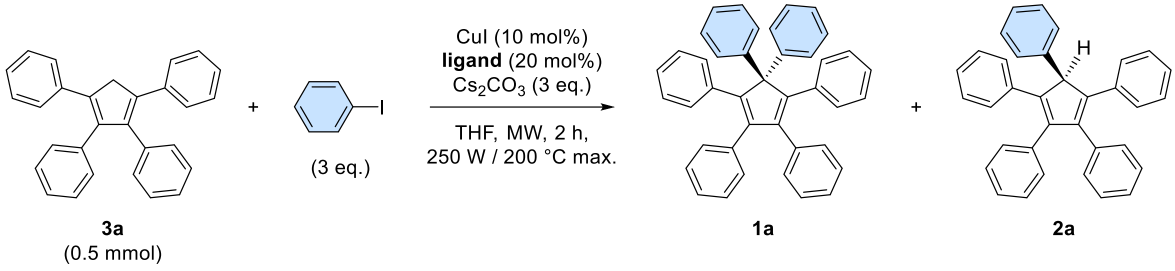

Entry	Ligand	1a (%)[a]	2a (%)[a]	1a + 2a (%)[a]
1	$Cy(NH_2)_2$ [b]	67	11	78
2	$Cy(NHMe)_2$ [c]	54	13	67
3	1,10-phenanthroline	19	23	42
4	2,2'-bipyridine	14	46	60
5	(±)-proline	32	39	71
6	diisopropylamine	7	42	49

[a] Compounds **1a** and **2a** were isolated as a mixture by column chromatography and the ratio of the two species was estimated by ^{1}H NMR spectroscopy. [b] $Cy(NH_2)_2$ = (±)-*trans*-1,2-cyclohexanediamine. [c] $Cy(NHMe)_2$ = (±)-*trans-N,N'*-dimethyl-1,2-cyclohexanediamine.

c. Influence of the copper source

In the following optimisation process, the influence of the copper source in the copper-catalysed arylation of tetraphenylcyclopentadiene **3a** was investigated, using the standard reaction conditions (involving 3 equivalents of iodobenzene, Table S1 entry 3) as a starting point.

Copper(I) iodide was first replaced with copper(I) bromide, as the latter demonstrated higher efficiency in examples of Hurtley reactions.[5b] This resulted in an important decrease of the overall yield of arylation products **1a** and **2a**, dropping from 78% to 39% after 2 h at 200 °C (Table S3, entries 1,2), thus showing the superiority of copper(I) iodide in this process. In addition, a reaction was set up using copper(I) thiophene-2-carboxylate, as this complex has already been successfully employed in Ullman-type couplings.[6] This did not lead to any arylation product, but a mixture of by-products was obtained instead (entry 3).

Table S3. Variation of the copper source in the copper-catalysed arylation of tetraphenylcyclopentadiene.

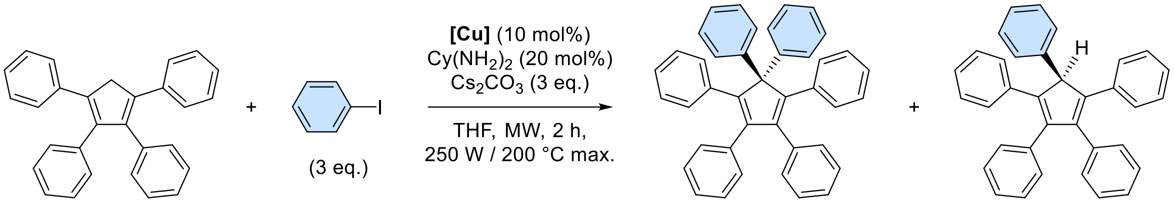

3a (0.5 mmol) | 1a | 2a

Entry	Copper source	1a (%)[a]	2a (%)[a]	1a + 2a (%)[a]
1	CuI	67	11	78
2	CuBr	29	10	39
3	CuTC[b,c]	0	0	0

[a] Compounds **1a** and **2a** were isolated as a mixture by column chromatography and the ratio of the two species was estimated by ^{1}H NMR spectroscopy. [b] CuTC (copper(I) thiophene-2-carboxylate) was used without additional ligand. [c] An over-pressurisation of the vial occurred after ca. 1.5 hour for all attempts, resulting in an early interruption of the reaction.

d. Influence of the solvent

In the following optimisation process, the influence of the solvent in the copper-catalysed arylation of tetraphenylcyclopentadiene **3a** was investigated, using the standard reaction conditions (involving 3 equivalents of iodobenzene, Table S1 entry 3) as a starting point.

When THF was replaced with its greener analogue 2-methyltetrahydrofuran[7] (Table S4, entry 2) or with 1,4-dioxane (entry 3), the overall yield in arylation products **1a** and **2a** remained similar, but the efficiency of the second arylation step dropped as witnessed by the yields obtained for hexaphenylcyclopentadiene **1a** (48% and 52% in MeTHF and 1,4-dioxane, respectively, compared to 67% in THF). With acetonitrile, the desired product **1a** was formed in amounts comparable to THF (64%), but full conversion of the starting material **3a** was reached after 2 h, thus indicating the involvement of competitive unproductive pathways (entry 4). DMF, the solvent of choice in the palladium-catalysed arylation reported by Dyker *et al.*,[4] was tested but failed to improve the efficiency of the reaction performed under microwave irradiation (entry 5). The arylation reaction proved to be compatible with low-polarity solvents such as toluene, even though hexaphenylcyclopentadiene **1a** was obtained in a low yield after 2 h (entry 6).

Finally, in spite of the insolubility of the starting material and arylation products, water was tested as a solvent for this reaction (entry 7). While only a very small amount of perarylated cyclopentadiene **1a** was formed (~1%), pentaphenylcyclopentadiene **2a** was obtained in 38% yield after 2 h, along with unreacted starting material. In this medium, the first arylation occurs while the second arylation is almost totally inhibited.

Table S4. Variation of the solvent in the copper-catalysed arylation of tetraphenylcyclopentadiene.

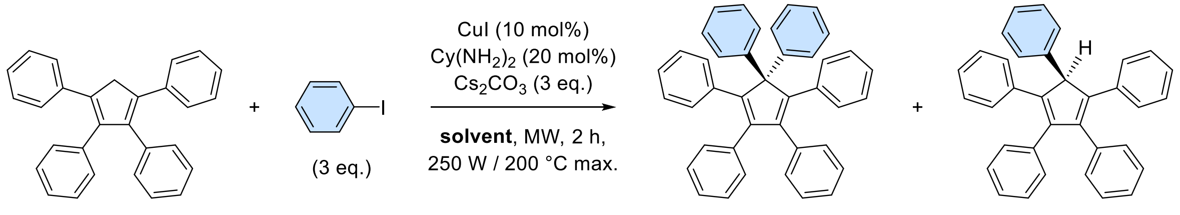

Entry	Solvent	1a (%)[a]	2a (%)[a]	1a + 2a (%)[a]
1	THF	67	11	78
2	Me-THF	48	26	74
3	1,4-dioxane	52	31	83
4	acetonitrile	64	1	65
5	DMF	30	15	45
6	toluene	16	53	67
7	water	1	38	39

[a] Compounds **1a** and **2a** were isolated as a mixture by column chromatography and the ratio of the two species was estimated by ^{1}H NMR spectroscopy.

e. Influence of the base

In the following optimisation process, the influence of the nature of the base in the copper-catalysed arylation of tetraphenylcyclopentadiene **3a** was investigated, using the standard reaction conditions (involving 3 equivalents of iodobenzene, Table S1 entry 3) as a starting point.

A screening of different carbonate species highlighted the major impact of the nature of the counter cation, with the yield of hexaphenylcyclopentadiene **1a** gradually dropping from 67% to 1% when moving from cesium to sodium ion (Table S5, entries 1-3). Further experiments showed that strong bases such as hydrides and *tert*-butoxides are tolerated in this reaction (entries 4-6), although yields are lower than those with cesium carbonate. Interestingly, sodium and lithium *tert*-butoxide afforded exclusively the perarylated product **1a** in 47 and 57% yield, respectively, thus underlining the high efficiency of the second arylation step in these conditions.

Table S5. Variation of the base in the copper-catalysed arylation of tetraphenylcyclopentadiene.

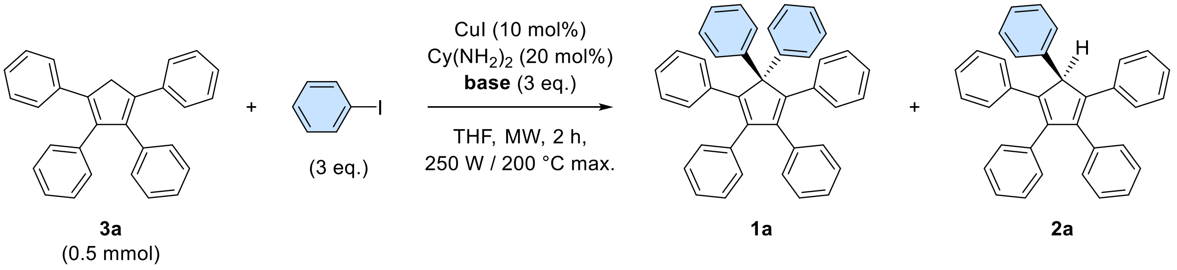

Entry	Base	1a (%)[a]	2a (%)[a]	1a + 2a (%)[a]
1	Cs_2CO_3	67	11	78
2	K_2CO_3	24	43	67
3	Na_2CO_3	1	14	15
4	NaH	55	10	65
5	NaO*t*Bu	47	0	47
6	LiO*t*Bu	57	0	57

[a] Compounds **1a** and **2a** were isolated as a mixture by column chromatography and the ratio of the two species was estimated by ^{1}H NMR spectroscopy. In cases when **2a** was not formed during the reaction, the pure product **1a** was then directly isolated by column chromatography.

II.2. Optimisation of the copper-catalysed perarylation of cyclopentadiene

a. Influence of the catalyst loading

The catalytic loading in the optimised conditions for the direct perarylation of cyclopentadiene, employing zirconocene dichloride as cyclopentadiene source, is 30 mol% of copper(I) iodide, which corresponds to 5 mol% of catalyst per new C-C bond formed during the reaction. To investigate the influence of catalyst loading on the perarylation efficiency (Table S6), stock solutions of copper(I) iodide in acetonitrile were prepared and it was verified in a preliminary experiment that addition of acetonitrile in the reaction medium has no impact on the reactivity (entry 2).

The catalyst loading was then reduced to 3 mol% (*i.e.* 0.5 mol% per new C-C bond), which resulted in a slightly higher yield of perarylated product **1a** (61%), as compared to 54% using 30 mol% of copper(I) (entry 3). No pentaphenylcyclopentadiene was observed in these conditions.

A further 10-fold reduction of the catalytic loading (*i.e.* 0.05 mol% per new C-C bond) remains productive but the yield of the sixth arylation is noticeably decreased, as shown by the equivalent amounts of hexa- and pentaphenylcyclopentadienes **1a** and **2a** obtained after 2 h (entry 4). It is important to mention that the low yield of 27% obtained here for the perarylated product **1a** still corresponds to an average yield of 80% per new bond, which remains acceptable for a cross-coupling reaction involving a catalyst loading of 500 ppm.

Finally, a blank experiment carried out in the absence of copper(I) iodide confirmed that the catalyst is crucial for the perarylation of cyclopentadiene (entry 5).

Table S6. Variation of the catalyst loading in the copper-catalysed perarylation of cyclopentadiene, using zirconocene dichloride as substrate.

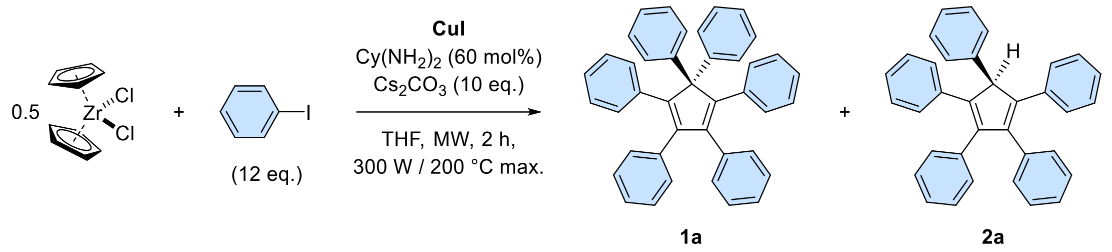

Entry	Catalyst loading (mol%) overall	per new C-C bond in **1a**	1a (%)[a]	2a (%)[a]	Average yield (%) per new C-C bond in 1a
1	30	5	54	0	90
2	30[b]	5	51	0	89
3	3[c]	0.5	61	0	92
4	0.3[d]	0.05	27	26	80
5	0	0	0	0	0

[a] Compounds **1a** and **2a** were isolated as a mixture by column chromatography and the ratio of the two species was estimated by ^{1}H NMR spectroscopy. In cases when **2a** was not formed during the reaction, pure product **1a** was then directly isolated by column chromatography. [b] Acetonitrile (20 µL) was added. [c] A stock solution of CuI in acetonitrile was used ([CuI] = 0.14 mol.L^{-1}, 20 µL). [d] A stock solution of CuI in acetonitrile was used ([CuI] = 0.014 mol.L^{-1}, 20 µL).

b. Influence of the thermal activation mode

In order to investigate the importance of microwave activation in this copper-catalysed process, the perarylation reaction was performed using classical thermal heating with a preheated oil bath at 200 °C instead of microwave irradiation, under otherwise unchanged conditions (Table S7). For conventional heating, the reaction was carried out in a screw-cap sealed Schlenk tube and the scale of the reaction was adjusted to control a similar pressure build-up as in microwave-activated reactions. After 2 h, only 5% of hexaphenylcyclopentadiene **1a** was obtained (entry 2), compared to 54 % under microwave irradiation (entry 1). Even after a prolonged reaction time of 24 h at 200 °C (entry 3), a modest yield of 20% was obtained, thus confirming the prime importance of microwave activation for the efficiency of the copper-catalysed perarylation reaction. It is important to note that the reaction medium is inhomogeneous, with the presence of solid copper and cesium salts, and it is inferred that hot-spots are produced under microwave irradiation,[8] thus resulting in locally increased temperatures.[9]

Table S7. Impact of the thermal activation mode in the copper-catalysed perarylation of cyclopentadiene, using zirconocene dichloride as substrate.

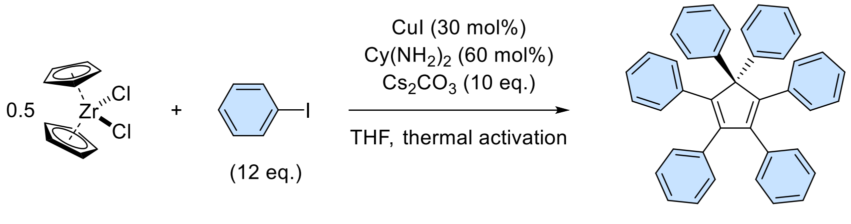

1a

Entry	Thermal activation mode	Duration	Yield (%)[a]
1[b]	MW, 300 W/ 200 °C max.	2 h	54
2[c]	preheated oil bath, 200 °C	2 h	5
3[c]	preheated oil bath, 200 °C	24 h	20

[a] Isolated yield. [b] The reaction was set up on a 0.048 mmol scale ($ZrCp_2Cl_2$) in 1 mL of THF in a 10 mL tube designed for microwave irradiation. [c] The reaction was set up on a 0.140 mmol scale ($ZrCp_2Cl_2$) in 3 mL of THF in a 30 mL sealable pressure Schlenk tube.

c. Scale up of the copper-catalysed perarylation reaction

The copper-catalysed perarylation reaction was scaled up (Table S8), using 100 mL PTFE microwave reactors instead of the 10 mL tubes used in routine experiments. A 20-fold increase of the reaction scale was performed and the reaction was performed on 0.96 mmol of zirconocene dichloride, *i.e.* 1.92 mmol of cyclopentadiene equivalent. Some reaction parameters were adjusted, such as the medium concentration and the available microwave power (set to 500 W), to afford after 2 h the desired hexaphenylcyclopentadiene **1a** in 27% isolated yield (267 mg).

Table S8. Scale up of the copper-catalysed perarylation of cyclopentadiene, using zirconocene dichloride as substrate.

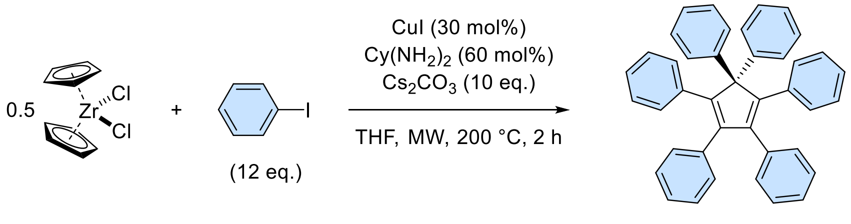

1a

Entry	Scale[a]	Volume of THF	MW available power	Yield (%)[b]	Average yield (%) per new C-C bond
1	0.048 mmol (*i.e.* 0.096 mmol of "Cp")	1 mL	300 W	54	90
2	0.96 mmol (*i.e.* 1.92 mmol of "Cp")	15 mL	500 W	27	80

[a] Amount of $ZrCp_2Cl_2$ used in the reaction, as source of two cyclopentadienyl moieties. [b] Isolated yield.

II.3. Mechanistic considerations

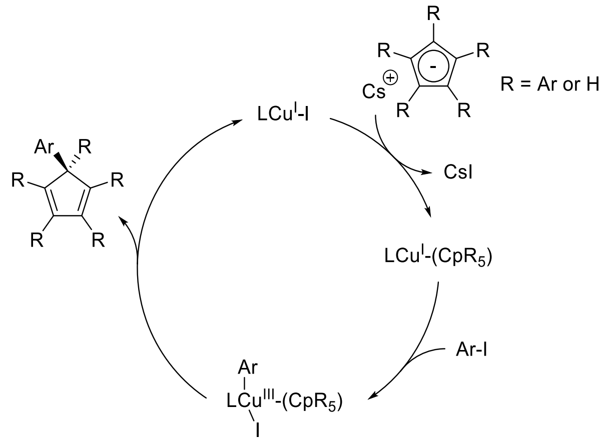

Scheme S2. Proposed mechanism for the arylation of cyclopentadiene catalysed by copper(I), involving the initial displacement of the iodide by the cyclopentadienide anion, followed by the oxidative addition of the iodoarene coupling partner to afford a Cu(III) intermediate and the subsequent reductive elimination to yield the arylated cyclopentadiene and release the catalytically-active copper(I) species. L = ligand.

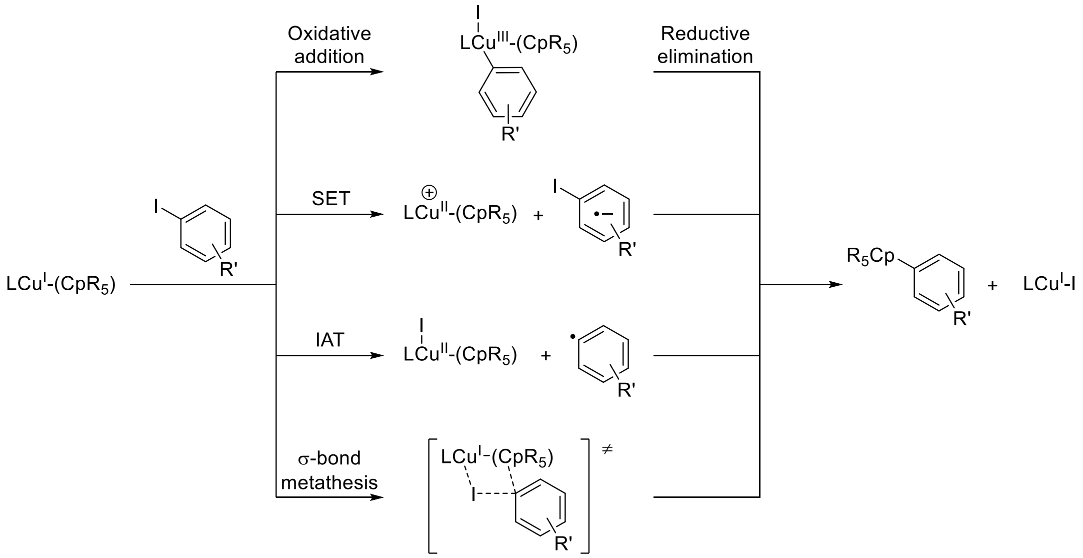

Scheme S3. Possible mechanistic pathways for the copper-mediated arylation of cyclopentadiene, after initial formation of the LCu^I(CpR$_5$) species *via* an halide displacement by cyclopentadienide: i) oxidative addition / reductive elimination sequence, ii) single-electron transfer (SET), iii) iodine atom transfer (IAT) and iv) four-centre σ-bond metathesis. L = ligand.

III. Experimental procedures and characterisation

Procedure A: *Copper-catalysed arylation of tetraphenylcyclopentadiene (model reaction)*

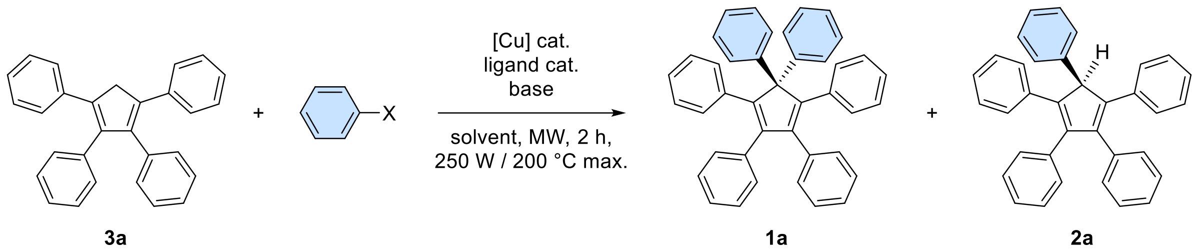

In a glovebox, 1,2,3,4-tetraphenylcyclopenta-1,3-diene[1] (**3a**) (185 mg, 0.50 mmol, 1.0 eq.), the appropriate base (1.51 mmol, 3.0 eq.), ligand (0.10 mmol, 20 mol%), copper(I) source (0.05 mmol, 10 mol%), anhydrous degassed solvent (2 mL) and a magnetic stir bar were placed in a 10 mL tube designed for microwave irradiation. The appropriate aryl halide (1.52 mmol, 3.0 eq. or 3.04 mmol, 6.0 eq.) was added and the suspension was briefly shaken before sealing the vial. The mixture was then heated using microwave irradiation at 200 °C for two hours, setting up the microwave with an available power of 250 W and a maximal pressure of 20 bars (typically the pressure stabilised between 10 and 15 bars depending on the conditions). After cooling down the reaction medium and carefully releasing the pressure, the reaction mixture was diluted with CH_2Cl_2 (10-20 mL) and *p*-toluenesulfonic acid (258 mg, 1.5 mmol, 3.0 eq.) was added. The resulting suspension was then filtered over a short celite plug (eluted with CH_2Cl_2) and the solvents were removed. The crude solid was dissolved in CH_2Cl_2, adsorbed onto silica and purified by column chromatography (SiO_2, CH_2Cl_2/pentane 20:80). Pentaphenylcyclopentadiene **2a** and hexaphenylcyclopentadiene **1a** were collected together (the corresponding fractions were combined if separated), filtered through a cotton wool plug and dried *in vacuo*. The ratio between the two species was estimated by ^{1}H NMR and the respective yields of products **1a** and **2a** were determined from the total mass of the fraction.

Procedure B: *Copper-catalysed perarylation of cyclopentadiene, using zirconocene dichloride as substrate*

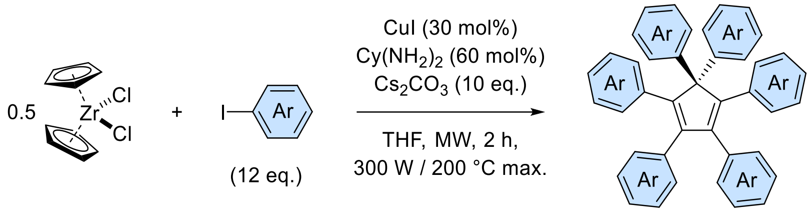

In a glovebox, zirconocene dichloride (14 mg, 0.048 mmol, 0.5 eq.), cesium carbonate (312 mg, 0.96 mmol, 10 eq.), (±)-*trans*-1,2-cyclohexanediamine (7 µL, 0.06 mmol, 60 mol%), copper(I) iodide (5.5 mg, 0.03 mmol, 30 mol%), anhydrous degassed THF (1 mL) and a magnetic stir bar were placed in a 10 mL tube designed for microwave irradiation. The appropriate aryl iodide (1.15 mmol, 12.0 eq.) was added and the suspension was briefly shaken before sealing the vial. The mixture was then heated using microwave irradiation at 200 °C for two hours, setting up the microwave with an available power of 300 W and a maximal pressure of 20 bars (typically the pressure stabilised between 10 and 15 bars depending on the conditions). After cooling down the reaction medium and carefully releasing the pressure, the reaction mixture was diluted with CH_2Cl_2 (10 mL) and filtered over a short celite plug (eluted with CH_2Cl_2). The solvents were removed and the crude product was dissolved in CH_2Cl_2, adsorbed onto silica and purified by column chromatography on silica gel to isolate the desired hexaarylated product.

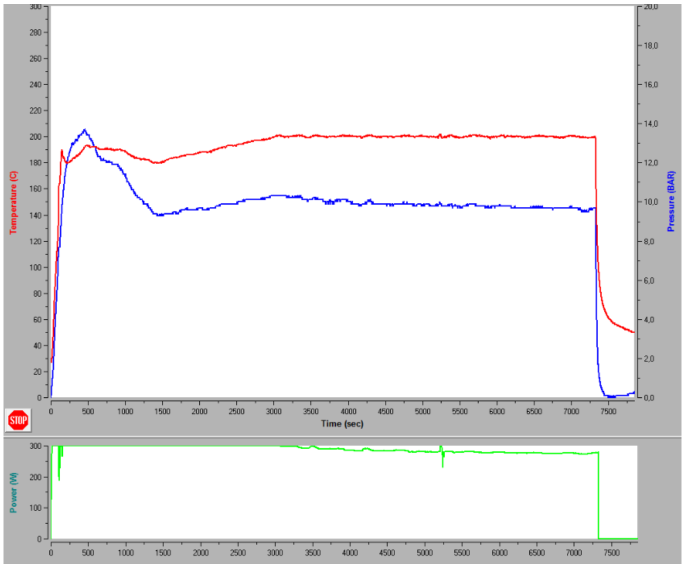

Figure S1. Heat/Pressure profile of a typical copper-catalysed perarylation reaction under microwave irradiation.

1,2,3,4,5,5-Hexaphenylcyclopenta-1,3-diene (1a):

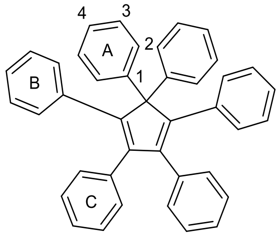

1,2,3,4,5,5-Hexaphenylcyclopenta-1,3-diene was synthesised from zirconocene dichloride according to the general **procedure B**, using iodobenzene (129 µL, 1.15 mmol, 12.0 eq.) as coupling partner. The compound was purified by column chromatography (SiO_2, CH_2Cl_2/pentane 10:90) to give pure hexaphenylcyclopentadiene **1a** as a white solid in 54% yield (26.9 mg, 51.4 µmol).

Scaled-up procedure: zirconocene dichloride (280 mg, 0.96 mmol, 0.5 eq.), cesium carbonate (6.242 g, 19.16 mmol, 10 eq.), copper(I) iodide (109 mg, 0.57 mmol, 30 mol%), (±)-*trans*-1,2-cyclohexanediamine (140 µL, 1.15 mmol, 60 mol%) and a magnetic stir bar were placed in a 100 mL Teflon microwave-reactor vessel in a glovebox. Degassed anhydrous THF (15 mL) and iodobenzene (2.58 mL, 22.99 mmol, 12 eq.) were successively added. The vessel was sealed and heated at 200 °C under microwave irradiation (500 W available) for two hours. After cooling down, the pressure was carefully released and the crude reaction medium was processed as described in **procedure B** for the smaller scale version of the reaction, to give pure hexaphenylcyclopentadiene **1a** in 27% yield (267 mg, 0.51 mmol).

Alternatively, this compound was also synthesised by the diarylation of 1,2,3,4-tetraphenylcyclopenta-1,3-diene (**3a**) according to the general **procedure A**. Under optimised conditions, 1,2,3,4-tetraphenyl-1,3-cyclopentadiene **3a** (185 mg, 0.50 mmol, 1.0 eq.), cesium carbonate (490 mg, 1.51 mmol, 3.0 eq.), (±)-*trans*-1,2-cyclohexanediamine (12 µL, 0.10 mmol, 20 mol%), copper(I) iodide (10 mg, 0.05 mmol, 10 mol%), anhydrous degassed THF (2 mL) and a magnetic stir bar were placed in a 10 mL tube designed for microwave irradiation, in a glovebox. Iodobenzene (338 µL, 3.04 mmol, 6.0 eq.) was added and the suspension was briefly shaken before sealing the vial. The mixture was then heated using microwave irradiation at 200 °C for two hours, setting up the microwave with an available power of 250W and a maximal pressure of 20 bars. After cooling down the reaction medium and carefully releasing the pressure, the reaction mixture was diluted with CH_2Cl_2 (10-20 mL) and *p*-toluenesulfonic acid (258 mg, 1.5 mmol, 3.0 eq.) was added. The resulting suspension was then filtered over a short celite plug (eluted with CH_2Cl_2) and the solvents were removed. The crude solid was dissolved in CH_2Cl_2, adsorbed onto silica and purified by column chromatography (SiO_2, CH_2Cl_2/pentane 20:80) to give hexaphenylcyclopentadiene **1a** as a white solid in 88% yield (231 mg, 0.44 mmol).

$\boldsymbol{R_f}$ = 0.39 (SiO_2, CH_2Cl_2/hexane 20:80). **m.p.:** 162 °C. **^{1}H NMR** (500 MHz, CD_2Cl_2, 25 °C): δ = 7.47 (m, 4H, H_{A2}), 7.23 – 7.18 (m, 6H, H_{A4} and H_{A3}), 7.14 – 7.09 (m, 6H, H_{C4} and H_{C3}), 7.03 (m, 4H, H_{C2}), 6.98 (tt, ^3J = 7.4 Hz, ^4J = 1.3 Hz, 2H, H_{B4}), 6.90 (virt. t, ^3J = 7.4 Hz, 4H, H_{B3}), 6.76 (m, 4H, H_{B2}) ppm. **^{13}C{^{1}H} NMR** (126 MHz, CD_2Cl_2, 25 °C): δ = 153.1 ($C^{Cp\text{-}B}$), 144.1 ($C^{Cp\text{-}C}$), 139.6 (C^{A1}), 136.5 (C^{C1}), 136.2 (C^{B1}), 130.8 (C^{B2} or C^{C2}), 130.6 (C^{B2} or C^{C2}), 129.4 (C^{A2}), 128.3 (C^{A3}), 128.0 (C^{C3}), 127.5 (C^{B3}), 127.1 (C^{A4} or C^{B4} or C^{C4}), 127.0 (C^{A4} or C^{B4} or C^{C4}), 126.8 (C^{A4} or C^{B4} or C^{C4}), 75.0 ($C^{Cp\text{-}A}$) ppm. **UV-Vis** (THF): λ_{max} (ε)= 247 (48000), 280 (sh, 29000), 340 nm (12500 $mol^{-1}dm^3cm^{-1}$). **HR-MS** (DCI-CH_4): calcd. for $C_{41}H_{30}$ $[M]^+$: 522.2348, found 522.2348. **Crystal data:** see section VII of the SI.

1,2,3,4,5,5-Hexa(4'-*tert*-butylphenyl)cyclopenta-1,3-diene (1b):

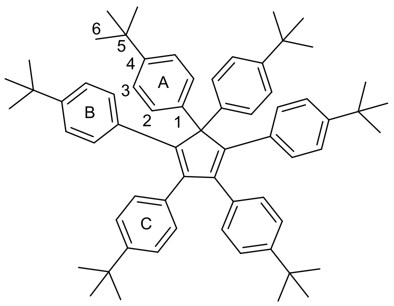

1,2,3,4,5,5-Hexa(4'-*tert*-butylphenyl)cyclopenta-1,3-diene was synthesised from zirconocene dichloride according to the general **procedure B**, using 1-*tert*-butyl-4-iodobenzene (205 µL, 1.15 mmol, 12.0 eq.) as coupling partner. The compound was purified by column chromatography (SiO_2, CH_2Cl_2/pentane 10:90) to give pure hexa(4-*tert*-butylphenyl)cyclopentadiene **1b** as a white solid in 37% yield (30.5 mg, 35.5 µmol).

***R*f** = 0.39 (SiO_2, CH_2Cl_2/hexane 10:90). **m.p.:** 150 °C. **^{1}H NMR** (500 MHz, CD_2Cl_2, 25 °C): δ = 7.32 (AA'BB' pattern, ^3J = 8.7 Hz, 4H, H_{A2}), 7.21 (AA'BB' pattern, ^3J = 8.7 Hz, 4H, H_{A3}), 7.10 (AA'BB' pattern, ^3J = 8.7 Hz, 4H, $H_{\beta3}$), 6.92 (AA'BB' pattern, ^3J = 8.8 Hz, 4H, $H_{\beta2}$ or $H_{\Upsilon3}$), 6.91 (AA'BB' pattern, ^3J = 8.7 Hz, 4H, $H_{\beta2}$ or $H_{\Upsilon3}$), 6.62 (AA'BB' pattern, ^3J = 8.8 Hz, 4H, $H_{\Upsilon2}$), 1.30 (s, 18H, H_{A6} or $H_{\beta6}$), 1.23 (s, 18H, H_{A6} or $H_{\beta6}$), 1.16 (s, 18H, $H_{\Upsilon6}$) ppm. Substituted aryls β and ϒ correspond to aryls B and C but could not be assigned certainly to either aryl using 2D NMR. **^{13}C{^{1}H} NMR** (126 MHz, CD_2Cl_2, 25 °C): δ = 152.1 ($C^{Cp-\Upsilon}$), 149.7 (C^{A4} and $C^{\beta4}$), 149.5 ($C^{\Upsilon4}$), 143.9 ($C^{Cp-\beta}$), 136.6 (C^{A1}), 134.1 ($C^{\beta1}$), 133.8 ($C^{\Upsilon1}$), 130.4 ($C^{\Upsilon2}$), 130.1 ($C^{\beta2}$), 129.3 (C^{A2}), 125.0 (C^{A3}), 124.7 ($C^{\beta3}$), 124.2 ($C^{\Upsilon3}$), 74.0 (C^{Cp-A}), 34.7 (C^{A5} or $C^{\beta5}$), 34.6 (C^{A5} or $C^{\beta5}$), 34.6 ($C^{\Upsilon5}$), 31.6 (C^{A6} or C^{B6}), 31.4 (C^{A6} or $C^{\beta6}$), 31.3 ($C^{\Upsilon6}$) ppm. **UV-Vis** (CH_2Cl_2): λ_{max} (ε)= 253 (29300), 282 (sh, 19600), 345 nm (7900 $mol^{-1}dm^3cm^{-1}$). **HR-MS** (DCI-CH_4): calcd. for $C_{65}H_{78}$ $[M]^+$: 858.6104, found 858.6069. **Crystal data:** see section VII of the SI.

1,2,3,4,5,5-Hexa(4'-tolyl)cyclopenta-1,3-diene (1c):

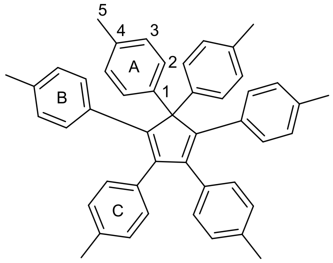

1,2,3,4,5,5-Hexa(4'-tolyl)cyclopenta-1,3-diene was synthesised from zirconocene dichloride according to the general **procedure B**, using 4-iodotoluene (251 mg, 1.15 mmol, 12.0 eq.) as coupling partner. The compound was purified by column chromatography (SiO_2, CH_2Cl_2/pentane 10:90) to give pure hexa(4-tolyl)cyclopentadiene **1c** as a white solid in 57% yield (33.2 mg, 54.7 µmol).

***R*f** = 0.59 (SiO_2, CH_2Cl_2/hexane 20:80). **m.p.:** 214 °C. **^{1}H NMR** (500 MHz, CD_2Cl_2, 25 °C): δ = 7.30 (AA'BB' pattern, ^3J = 8.2 Hz, 4H, H_{A2}), 7.00 (AA'BB' pattern, ^3J = 7.9 Hz, 4H, H_{A3}), 6.92 (AA'BB' pattern, ^3J = 7.9 Hz, 4H, H_{C3}), 6.88 (AA'BB' pattern, ^3J = 8.2 Hz, 4H, H_{C2}), 6.70 (AA'BB' pattern, ^3J = 8.0 Hz, 4H, H_{B3}), 6.61 (AA'BB' pattern, ^3J = 8.2 Hz, 4H, H_{B2}), 2.29 (s, 6H, H_{A5}), 2.25 (s, 6H, H_{C5}), 2.14 (s, 6H, H_{B5}) ppm. **^{13}C{^{1}H} NMR** (126 MHz, CD_2Cl_2, 25 °C): δ = 152.5 (C^{B4}), 143.5 (C^{C4}), 136.9 (C^{A4}), 136.5 (C^{C1}), 136.3 (C^{B1} or C^{A1}), 136.3 (C^{B1} or C^{A1}), 134.0 (C^{Cp-C}), 133.4 (C^{Cp-B}), 130.6 (C^{B3}), 130.5 (C^{C2}), 129.4 (C^{A2}), 128.8 (C^{A3}), 128.7 (C^{C3}), 128.1 (C^{B2}), 73.7 (C^{Cp-A}), 21.3 (C^{A5}), 21.2 (C^{B5} or C^{C5}), 21.1 (C^{B5} or C^{C5}) ppm. **UV-Vis** (CH_2Cl_2): λ_{max} (ε)= 253 (29600), 280 (sh, 21200), 348 nm (8700 $mol^{-1}dm^3cm^{-1}$). **HR-MS** (DCI-CH_4): calcd. for $C_{47}H_{42}$ $[M]^+$: 606.3287, found 606.3306. **Crystal data:** see section VII of the SI.

1,2,3,4,5,5-Hexa(3'-tolyl)cyclopenta-1,3-diene (1d):

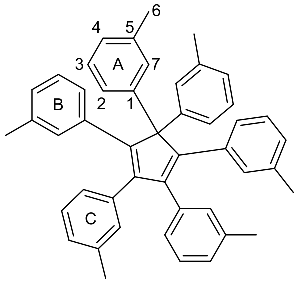

1,2,3,4,5,5-Hexa(3'-tolyl)cyclopenta-1,3-diene was synthesised from zirconocene dichloride according to the general **procedure B**, using 3-iodotoluene (148 µL, 1.15 mmol, 12.0 eq.) as coupling partner. The compound was purified by column chromatography (SiO_2, CH_2Cl_2/pentane 15:85) to give pure hexa(3-tolyl)cyclopentadiene **1d** as a white solid in 50% yield (29.1 mg, 48.0 µmol).

***R*f** = 0.14 (SiO_2, CH_2Cl_2/hexane 10:90). **m.p.:** 152 °C. **^{1}H NMR** (500 MHz, CD_2Cl_2, 25 °C): δ = 7.27 (d, ^3J = 7.7 Hz, 2H, H_{A2}), 7.19 (s, 2H, H_{A7}), 7.11 (virt. t, ^3J = 7.7 Hz, 2H, H_{A3}), 7.02 (d, ^3J = 7.7 Hz, 2H, H_{A4}), 6.99 (virt. t, ^3J = 7.7 Hz, 2H, H_{B3}), 6.93 (d, ^3J = 7.7 Hz, 2H, H_{B4}), 6.87 – 6.78 (m, 8H, H_{B2}, $H_{B7,}$ H_{C3}, H_{C4}), 6.61 – 6.55 (m, 2H, H_{C2}), 6.48 (s, 2H, H_{C7}), 2.23 (s, 6H, H_{A6}), 2.13 (s, 6H, H_{B6}), 1.98 (s, 6H, H_{C6}) ppm. **^{13}C{^{1}H} NMR** (126 MHz, CD_2Cl_2, 25 °C): δ = 152.5 (C^{C1}), 144.1 (C^{B1}), 139.6 (C^{A1}), 137.6 (C^{A5}), 137.4 (C^{B5}), 136.7 (C^{Cp-B}), 136.6 (C^{C5}), 136.3 (C^{Cp-C}), 131.7 (C^{C7}), 131.3 (C^{B7}), 130.7 (C^{A7}), 128.1 (C^{A3} or C^{C3}), 128.0 (C^{A3} or C^{C3}), 127.7 (C^{CH-Ar}), 127.6 (C^{CH-Ar}), 127.6 (C^{CH-Ar}), 127.5 (C^{CH-Ar}), 127.4 (C^{CH-Ar}), 127.1 (C^{CH-Ar}), 126.5 (C^{A2}), 74.7 (C^{Cp-A}), 21.7 (C^{A6}), 21.4 (C^{B6} and C^{C6}) ppm. C^{CH-Ar} = C^{A4}, C^{B2}, C^{B3}, C^{B4}, C^{C2}, C^{C4} which could not be assigned certainly using 2D NMR. **UV-Vis** (CH_2Cl_2): λ_{max} (ε)= 251 (20700), 284 (sh, 11300), 342 nm (5900 mol^{-1}dm^{3}cm^{-1}). **HR-MS** (DCI-CH_4): calcd. for $C_{47}H_{42}$ $[M]^+$: 606.3287, found 606.3381. **Crystal data:** see section VII of the SI.

1,2,3,4,5,5-Hexa(naphth-2'-yl)cyclopenta-1,3-diene (1f):

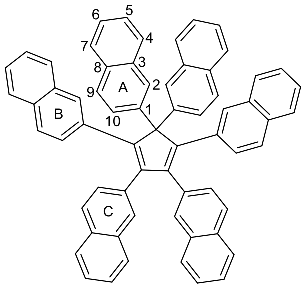

1,2,3,4,5,5-Hexa(naphth-2'-yl)cyclopenta-1,3-diene was synthesised from zirconocene dichloride according to the general **procedure B**, using 2-iodonaphthalene (292 mg, 1.15 mmol, 12.0 eq.) as coupling partner. The compound was purified by column chromatography (SiO_2, CH_2Cl_2/pentane gradient from 0:100 to 10:90) to give pure hexa(naphth-2-yl)cyclopentadiene **1f** as a pale-yellow solid in 46% yield (36.0 mg, 43.7 µmol).

***R*f** = 0.16 (SiO_2, CH_2Cl_2/hexane 20:80). **m.p.:** 180 °C. **^{1}H NMR** (500 MHz, CD_2Cl_2, 25 °C): δ = 8.16 (d, ^4J = 2.3 Hz, 2H, H_{2A}), 7.85 – 7.77 (m, 6H, H_{9A}, H_{10A} and H_7), 7.72 – 7.65 (m, 6H, H_4, H_7 and H_2), 7.57 (AA'BB' pattern, ^3J = 8.4 Hz, 2H, H_9), 7.51 (AA'BB' pattern, ^3J = 8.9 Hz, 4H, H_4), 7.46 (d, ^4J = 2.0 Hz, 2H, H_2), 7.45 – 7.16 (m, 18H, H_5, H_6, H_{7C}, H_9 and H_{10}), 6.87 (dd, ^3J = 8.6 Hz, ^4J = 1.8 Hz, 2H, H_{10}) ppm. **^{13}C{^{1}H} NMR** (126 MHz, CD_2Cl_2, 25 °C): δ = 153.6 (C^{Cp-B} or C^{Cp-C}), 145.1 (C^{Cp-B} or C^{Cp-C}), 137.4 (C^{1A}), 134.1 ($C^{quat-Ar}$), 133.8 ($C^{quat-Ar}$), 133.6 ($C^{quat-Ar}$), 133.5 ($C^{quat-Ar}$), 133.0 (C^{1B} or C^{1C}), 132.9 (C^{8A}), 132.7 (C^{1B} or C^{1C}), 132.4 (C^{8B} or C^{8C}), 129.9 (C^{2B} and C^{2C}), 129.1 (C^{CH-Ar}), 128.7 (C^{10B} and C^{10C}), 128.3 (C^{2A}), 128.3 (C^{CH-Ar}), 128.2 (C^{CH-Ar}), 128.0 (C^{CH-Ar}), 127.9 (C^{CH-Ar}), 127.8 (C^{CH-Ar}), 127.7 (C^{CH-Ar}), 127.6 (C^{CH-Ar}), 127.6 (C^{CH-Ar}), 126.8 (C^{CH-Ar}), 126.3 (C^{CH-Ar}), 126.3 (C^{CH-Ar}), 126.2 (C^{CH-Ar}), 126.2 (C^{CH-Ar}), 126.1 (C^{CH-Ar}), 125.9 (C^{CH-Ar}), 75.3 (C^{Cp-A}) ppm. Some NMR signals corresponding to aromatic CH and quaternary carbons could not be precisely assigned due to important overlapping in narrow ranges of the ^{13}C NMR spectrum. **UV-Vis** (CH_2Cl_2): λ_{max} (ε)= 272 (sh, 52000), 315 (sh, 21100), 330 (sh, 15700), 372 nm (sh, 900 mol^{-1}dm^{3}cm^{-1}). **HR-MS** (DCI-CH_4): calcd. for $C_{65}H_{42}$ $[M]^+$: 822.3287, found 822.3253.

1,2,3,4,5,5-Hexa(*p*-biphenyl)cyclopenta-1,3-diene (1g):

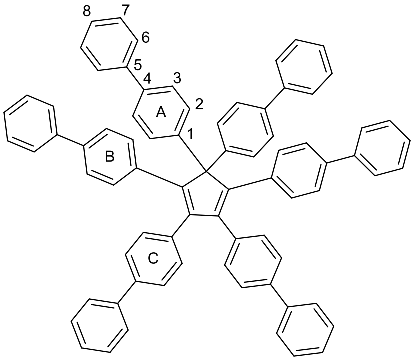

1,2,3,4,5,5-Hexa(*p*-biphenyl)cyclopenta-1,3-diene was synthesised from zirconocene dichloride according to the general **procedure B**, using 4-iodobiphenyl (322 mg, 1.15 mmol, 12.0 eq.) as coupling partner. The compound was purified by column chromatography (SiO_2, CH_2Cl_2/pentane gradient from 20:80 to 30:70) to give pure hexa(*p*-biphenyl)cyclopentadiene **1g** as a pale-yellow solid in 58% yield (54.8 mg, 56.0 µmol).

***R*$_f$** = 0.13 (SiO_2, CH_2Cl_2/hexane 20:80). **m.p.:** 165 °C. **^{1}H NMR** (500 MHz, CD_2Cl_2, 25 °C): δ = 7.68 (AA'BB' pattern, ^3J = 8.2 Hz, 4H, H_{2A}), 7.64 (dd, ^3J = 8.4 Hz, ^4J = 1.2 Hz, 4H, H_7), 7.59 (dd, ^3J = 8.4 Hz, ^4J = 1.2 Hz, 4H, H_7), 7.57 (AA'BB' pattern, ^3J = 8.7 Hz, 4H, H_{3A}), 7.50 – 7.45 (m, 8H, H_{3C} and H_7), 7.45 – 7.22 (m, 26H, H_6, H_8, H_{2C} and H_{3B}), 7.00 (AA'BB' pattern, ^3J = 8.8 Hz, 4H, H_{2B}) ppm. **^{13}C{^{1}H} NMR** (126 MHz, CD_2Cl_2, 25 °C): δ = 152.9 (C^{1B}), 144.1 (C^{1C}), 140.8 (C^4), 140.8 (C^4), 140.7 (C^4), 139.7 (C^{1A} or C^5), 139.6 (C^{1A} or C^5), 139.3 (C^{1A} or C^5), 138.8 (C^{1A} or C^5), 135.6 ($C^{Cp\text{-}C}$), 135.1 ($C^{Cp\text{-}B}$), 131.3 (C^{2B} or C^{2C}), 131.2 (C^{2B} or C^{2C}), 130.0 (C^{2A}), 129.2 (C^6), 129.1 (C^6), 129.1 (C^6), 127.7 (C^8), 127.7 (C^8), 127.6 (C^8), 127.2 (C^{3A} or C^7), 127.2 (C^{3A} or C^7), 127.0 (C^{3A} or C^7), 127.0 (C^{3A} or C^7), 126.7 (C^{3C}), 126.1 (C^{3B}), 74.3 ($C^{Cp\text{-}A}$) ppm. **UV-Vis** (CH_2Cl_2): λ_{max} (ε)= 277 (92400), 312 (sh, 51500), 306 nm (15800 $mol^{-1}dm^3cm^{-1}$). **HR-MS** (DCI-CH_4): calcd. for $C_{77}H_{54}$ $[M]^+$: 978.4226, found 978.4240.

1,2,3,4,5,5-Hexa(4'-methoxyphenyl)cyclopenta-1,3-diene (1h):

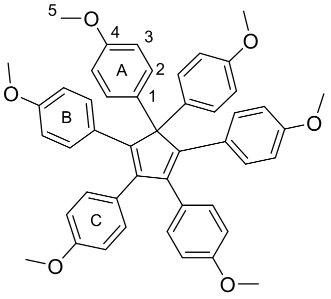

1,2,3,4,5,5-Hexa(4'-methoxyphenyl)cyclopenta-1,3-diene was synthesised from zirconocene dichloride according to the general **procedure B**, using 4-iodoanisole (269 mg, 1.15 mmol, 12.0 eq.) as coupling partner. The compound was purified by column chromatography (SiO_2, ethyl acetate/cyclohexane 30:70) to give pure hexa(4-methoxyphenyl) cyclopentadiene **1h** as a white solid in 10% yield (6.4 mg, 9.11 µmol).

***R*$_f$** = 0.66 (SiO_2, EtOAc/hexane 40:60). **m.p.:** 242 °C. **^{1}H NMR** (500 MHz, CD_2Cl_2, 25 °C): δ = 7.30 (AA'BB' pattern, ^3J = 9.0 Hz, 4H, H_{A2}), 6.90 (AA'BB' pattern, ^3J = 9.0 Hz, 4H, H_{B3}), 6.72 (AA'BB' pattern, ^3J = 9.0 Hz, 4H, H_{A3}), 6.67 (AA'BB' pattern, ^3J = 9.0 Hz, 4H, H_{C3}), 6.66 (AA'BB' pattern, ^3J = 9.0 Hz, 4H, H_{B2}), 6.45 (AA'BB' pattern, ^3J = 9.0 Hz, 4H, H_{C2}), 3.75 (s, 6H, H_{A5}), 3.72 (s, 6H, H_{B5}), 3.64 (s, 6H, H_{C5}) ppm. **^{13}C{^{1}H} NMR** (126 MHz, CD_2Cl_2, 25 °C): δ = 158.6 (C^{B4}), 158.5 (C^{A4}), 158.3 (C^{C4}), 151.8 (C^{C1}), 142.4 (C^{B1}), 132.2 (C^{A1}), 131.9 (C^{B3} or C^{C3}), 131.8 (C^{B3} or C^{C3}), 130.5 (C^{A2}), 129.3 ($C^{Cp\text{-}B}$), 129.0 ($C^{Cp\text{-}C}$), 113.4 (C^{A3} and C^{B2}), 112.8 (C^{C2}), 73.0 ($C^{Cp\text{-}A}$), 55.5 (C^{A5}), 55.4 (C^{B5}), 55.3 (C^{C5}) ppm. **UV-Vis** (THF): λ_{max} (ε)= 237 (50000), 258 (39500), 287 (35500), 352 nm (11500 $mol^{-1}dm^3cm^{-1}$). **HR-MS** (DCI-CH_4): calcd. for $C_{47}H_{42}O_6$ $[M]^+$: 702.2981, found 702.2957. **Crystal data:** see section VII of the SI.

1,2,3,4,5,5-Hexa(3’-methoxyphenyl)cyclopenta-1,3-diene (1i):

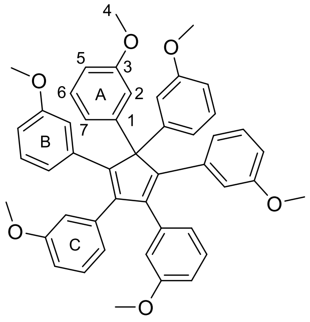

1,2,3,4,5,5-Hexa(3’-methoxyphenyl)cyclopenta-1,3-diene was synthesised from zirconocene dichloride according to the general **procedure B**, using 3-iodoanisole (269 mg, 1.15 mmol, 12.0 eq.) as coupling partner. The compound was purified by column chromatography (SiO_2, ethyl acetate/cyclohexane 20:80) to give pure hexa(3-methoxyphenyl) cyclopentadiene **1i** as an amorphous transparent film in 28% yield (18.8 mg, 26.7 µmol).

***R*f** = 0.21 (SiO_2, EtOAc/hexane 20:80). **^{1}H NMR** (500 MHz, CD_2Cl_2, 25 °C): δ = 7.16 (virt. t, ^3J = 7.9 Hz, 2H, H_{A6}), 7.11 (ddd, ^3J = 7.9 Hz, ^4J = 1.7 Hz, ^4J = 1.1 Hz, 2H, H_{A7}), 7.05 (virt. t, ^3J = 8.0 Hz, 2H, $H_{β6}$), 6.98 (br. s, 2H, H_{A2}), 6.86 (virt. t, ^3J = 8.0 Hz, 2H, $H_{γ6}$), 6.74 (ddd, ^3J = 8.0 Hz, ^4J = 2.6 Hz, ^4J = 1.1 Hz, 2H, H_{A5}), 6.69 (ddd, ^3J = 8.3 Hz, ^4J = 2.6 Hz, ^4J = 1.0 Hz, 2H, $H_{β5}$), 6.66 (ddd, ^3J = 7.6 Hz, ^4J = 1.5 Hz, ^4J = 1.0 Hz, 2H, $H_{β7}$), 6.59 – 6.52 (m, 4H, $H_{γ5}$ and $H_{β2}$), 6.44 (ddd, ^3J = 7.8 Hz, ^4J = 1.6 Hz, ^4J = 1.0 Hz, 2H, $H_{γ7}$), 6.34 (br. s, 2H, $H_{γ2}$), 3.62 (s, 6H, H_{A4} or $H_{β4}$), 3.54 (s, 6H, H_{A4} or $H_{β4}$), 3.39 (s, 6H, $H_{γ4}$) ppm. Substituted aryls β and ϒ correspond to aryls B and C but could not be assigned certainly to either aryl using 2D NMR. **^{13}C{^{1}H} NMR** (126 MHz, CD_2Cl_2, 25 °C): δ = 159.6 (C^{A3} or $C^{β3}$), 159.5 (C^{A3} or $C^{β3}$), 158.8 ($C^{γ3}$), 152.4 ($C^{γ1}$), 144.0 ($C^{β1}$), 141.0 (C^{A1}), 137.9 ($C^{Cp-β}$), 137.4 ($C^{Cp-γ}$), 129.1 (C^{A6} or $C^{β6}$), 129.1 (C^{A6} or $C^{β6}$), 128.4 ($C^{γ6}$), 123.4 ($C^{γ7}$), 123.0 ($C^{β7}$), 121.7 (C^{A7}), 116.1 ($C^{γ2}$), 115.9 ($C^{β2}$), 115.6 (C^{A2}), 113.0 ($C^{γ5}$ or $C^{β5}$), 112.9 ($C^{γ5}$ or $C^{β5}$), 112.6 (C^{A5}), 74.6 (C^{Cp-A}), 55.4 (C^{A4} and $C^{β4}$), 55.1 ($C^{γ4}$) ppm. **UV-Vis** (CH_2Cl_2): $λ_{max}$ (ε)= 287 (16900), 347 nm (6700 $mol^{-1}dm^3cm^{-1}$). **Fluorescence** (CH_2Cl_2): $λ_{ex}$ = 300 nm, $λ_{em}$ = 432 nm. **HR-MS** (DCI-CH_4): calcd. for $C_{47}H_{42}O_6$ $[M]^+$: 702.2981, found 702.2978.

1,2,3,4,5,5-Hexa(4’-fluorophenyl)cyclopenta-1,3-diene (1j):

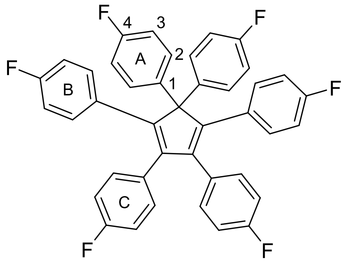

1,2,3,4,5,5-Hexa(4’-fluorophenyl)cyclopenta-1,3-diene was synthesised from zirconocene dichloride according to the general **procedure B**, using 1-fluoro-4-iodobenzene (133 µL, 1.15 mmol, 12.0 eq.) as coupling partner. The compound was purified by column chromatography (SiO_2, CH_2Cl_2/pentane 20:80) to give pure hexa(4-fluorophenyl)cyclopentadiene **1j** as a pale-yellow solid in 29% yield (17.7 mg, 28.1 µmol).

***R*f** = 0.50 (SiO_2, CH_2Cl_2/hexane 20:80). **m.p.:** 86 °C. **^{1}H NMR** (500 MHz, CD_2Cl_2, 25 °C): δ = 7.33 (dd, ^3J = 9.1 Hz, $^4J_{H-F}$ = 5.3 Hz, 4H, H_{A2}), 6.95 (dd, ^3J = 8.9 Hz, $^4J_{H-F}$ = 5.5 Hz, 4H, H_{C2}), 6.91 (dd, ^3J = 8.8 Hz, $^3J_{H-F}$ = 8.8 Hz, 4H, H_{A3}), 6.84 (dd, ^3J = 8.9 Hz, $^3J_{H-F}$ = 8.9 Hz, 4H, H_{C3}), 6.70 (dd, ^3J = 9.0 Hz, $^4J_{H-F}$ = 5.6 Hz, 4H, H_{B2}), 6.65 (dd, ^3J = 8.9 Hz, $^3J_{H-F}$ = 8.9 Hz, 4H, H_{B3}) ppm. **^{13}C{^{1}H} NMR** (126 MHz, CD_2Cl_2, 25 °C): δ = 162.3 (d, $^1J_{C-F}$ = 246.2 Hz, C^{C4}), 162.2 (d, $^1J_{C-F}$ = 246.0 Hz, C^{A4}), 162.0 (d, $^1J_{C-F}$ = 246.5 Hz, C^{B4}), 152.1 (C^{Cp-B}), 142.8 (C^{Cp-C}), 134.8 (d, $^4J_{C-F}$ = 3.4 Hz, C^{A1}), 132.8 (d, $^3J_{C-F}$ = 7.8 Hz, C^{C2} or C^{B2}), 132.8 (d, $^3J_{C-F}$ = 7.8 Hz, C^{B2} or C^{C2}), 131.7 (d, $^4J_{C-F}$ = 3.5 Hz, C^{B1}), 131.6 (d, $^4J_{C-F}$ = 3.5 Hz, C^{C1}), 130.7 (d, $^3J_{C-F}$ = 7.6 Hz, C^{A2}), 115.4 (d, $^2J_{C-F}$ = 21.3 Hz, C^{A3} or C^{C3}), 115.3 (d, ^2J = 21.3 Hz, C^{A3} or C^{C3}), 114.8 (d, ^2J = 21.2 Hz, C^{B3}), 74.0 (C^{Cp-A}) ppm. **^{19}F NMR** (471 MHz, CD_2Cl_2, 25 °C): δ = -115.2 (m, 4F), -116.2 (m, 2F). **^{19}F{^{1}H}** NMR (376 MHz, CD_2Cl_2, 25 °C): δ = -115.2, -115.2, -116.2 ppm. **UV-**

Vis (CH_2Cl_2): λ_{max} (ε)= 254 (sh, 12200), 320 nm (3400 $mol^{-1}dm^3cm^{-1}$). **HR-MS** (DCI-CH_4): calcd. for $C_{41}H_{24}F_6$ $[M]^+$: 630.1782, found 630.1777.

1,2,3,4,5,5-Hexa(4'-bromophenyl)cyclopenta-1,3-diene (1k):

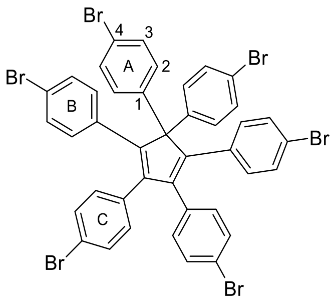

1,2,3,4,5,5-Hexa(4'-bromophenyl)cyclopenta-1,3-diene was synthesised from zirconocene dichloride according to the general **procedure B**, using 1-bromo-4-iodobenzene (325 mg, 1.15 mmol, 12.0 eq.) as coupling partner. The compound was purified by column chromatography (SiO_2, CH_2Cl_2/pentane gradient from 10:90 to 20:80) to give hexa(4-bromophenyl)cyclopentadiene **1k** as a transparent amorphous film in 7% yield (7.0 mg, 7.0 µmol). It was evidenced that the sample incorporated partially dehalogenated products, which were not separable by chromatography due to similar polarities.

***R*$_f$** = 0.39 (SiO_2, CH_2Cl_2/hexane 20:80). **^{1}H NMR** (500 MHz, CD_2Cl_2, 25 °C): δ = 7.35 (AA'BB' pattern, ^3J = 8.5 Hz, 4H, H_{Ar}), 7.29 (AA'BB' pattern, ^3J = 8.4 Hz, 4H, H_{Ar}), 7.24 – 7.18 (m, 4H, H_{Ar}), 7.09 (AA'BB' pattern, ^3J = 8.5 Hz, 4H, H_{Ar}), 6.86 – 6.81 (m, 4H, H_{Ar}), 6.59 (AA'BB' pattern, ^3J = 8.5 Hz, 4H, H_{Ar}) ppm. **^{13}C{^{1}H} NMR** (126 MHz, CD_2Cl_2, 25 °C): δ = 152.1 (C^{Cp-B}), 143.3 (C^{Cp-C}), 137.5 ($C^{quat-Ar}$), 134.1 ($C^{quat-Ar}$), 134.0 ($C^{quat-Ar}$), 132.1 (C^{CH-Ar}), 132.1 (C^{CH-Ar}), 131.9 (C^{CH-Ar}), 131.7 (C^{CH-Ar}), 131.3 (C^{CH-Ar}), 130.7 (C^{CH-Ar}), 122.0 ($C^{quat-Ar}$), 121.8 ($C^{quat-Ar}$), 121.5 ($C^{quat-Ar}$), 74.1 (C^{Cp-A}) ppm. Signals corresponding to partially dehalogenated products were also observed on ^{1}H and ^{13}C NMR spectra, but only signals corresponding to the title compound are described above. The overall proportion of debrominated phenyl moieties was estimated to be around ca. 20-25%. **UV-Vis** (CH_2Cl_2): λ_{max} (ε)= 257 (17600), 281 (11800), 350 nm (3900 $mol^{-1}dm^3cm^{-1}$). **HR-MS** (ESI^-): calcd. for $C_{42}H_{25}Br_6O_2$ $[M+HCOO]^-$: 1040.6901, found 1040.6884.

1,2,3,4,5-Pentaphenylcyclopenta-1,3-diene (2a):

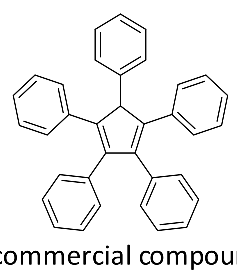

1,2,3,4,5-Pentaphenylcyclopenta-1,3-diene (**2a**) was obtained in various amounts during the optimisation of the model reaction starting from 1,2,3,4-tetraphenylcyclopenta-1,3-diene (**3a**), according to general **procedure A**.

^{1}H NMR (300 MHz, $CDCl_3$, 25 °C): δ = 7.24 – 6.93 (m, 30H), 5.08 (s, 1H) ppm. **LR-MS** (DCI-NH_3) *m/z*: 447 $[M+H]^+$. These data are in full agreement with those of the commercial compound.

Spiro[*1H*-cyclopenta[*l*]phenanthrene-1,9'-*[9H]*fluorene] (4):

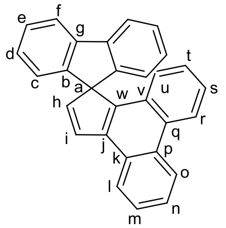

Spiro[*1H*-cyclopenta[*l*]phenanthrene-1,9'-*[9H]*fluorene] was synthesised from zirconocene dichloride according to the general **procedure B**, using 2,2'-diiodobiphenyl[2] (156 mg, 0.38 mmol, 4.0 eq.) as coupling partner. The compound was purified by preparative TLC (SiO_2, CH_2Cl_2/pentane 20:80) to give pure spirofluorene **4** as a transparent oil in 54% yield (18.8 mg, 51.4 µmol).

***R*$_f$** = 0.40 (SiO_2, CH_2Cl_2/hexane 20:80). **^{1}H NMR** (500 MHz, CD_2Cl_2, 25 °C): δ = 8.79 (d, ^3J = 8.0 Hz, 1H, H_o), 8.71 – 8.66 (m, ^3J = 8.4 Hz, 1H, H_r), 8.37 – 8.34 (m, 1H, H_l), 7.98 (ddd, ^3J = 7.7 Hz, ^4J = 1.1 Hz, ^5J = 0.7 Hz, 2H, H_f), 7.79 – 7.71 (m, 2H, H_m and H_n), 7.68 (d, ^3J = 5.4 Hz, 1H, H_i), 7.45 – 7.38 (m, 3H, H_s and H_e), 7.18 – 7.07 (m, 3H, H_d and H_t), 6.81 (ddd, ^3J = 7.6 Hz, ^4J = 1.1 Hz, ^5J = 0.7 Hz, 2H, H_c), 6.61 (ddd, ^3J = 8.2 Hz, ^4J = 1.4 Hz, ^5J = 0.6 Hz, 1H, H_u), 6.45 (d, ^3J = 5.3 Hz, 1H, H_h) ppm. **^{13}C{^{1}H} NMR** (126 MHz, CD_2Cl_2, 25 °C): δ = 145.0 (C^g), 143.4 (C^h), 142.4 (C^b), 141.4 (C^j), 140.1 (C^w), 131.4 (C^k), 130.1 (C^v), 129.3 (C^i), 128.3 (C^d), 128.2 (C^q), 128.1 (C^e), 127.9 (C^p), 127.2 (C^m or C^n), 127.0 (C^t), 126.8 (C^m or C^n), 125.5 (C^s), 125.1 (C^l), 123.9 (C^c), 123.9 (C^u), 123.7 (C^o), 123.6 (C^r), 121.2 (C^f), 70.5 (C^a) ppm. **UV-Vis** (CH_2Cl_2): λ_{max} (ε)= 242 (sh, 63100), 247 (65500), 260 (54500), 274 (sh, 38400), 285 (sh, 18200), 314 (10200), 328 (9600), 344 nm (8300 $mol^{-1}dm^3cm^{-1}$). **HR-MS** (DCI-CH_4): calcd. for $C_{29}H_{18}$ $[M]^+$: 366.1409, found 366.1413.

1,1,2,3-Tetraphenyl-*1H*-cyclopenta[*l*]phenanthrene (5):

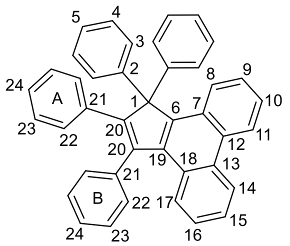

1,1,2,3-Tetraphenyl-*1H*-cyclopenta[*l*]phenanthrene was synthesised using a variation of the general **procedure A**. 1,2,3,4-Tetraphenylcyclopenta-1,3-diene (185 mg, 0.5 mmol, 1.0 eq.) was placed in a 10 mL microwave reactor equipped with a magnetic stir bar in a glovebox. Cesium carbonate (489 mg, 1.5 mmol, 3.0 eq.), 2,2'-diiodobiphenyl[2] (223 mg, 0.55 mmol, 1.1 eq.), copper(I) iodide (9.5 mg, 0.05 mmol, 10 mol%), (±)-*trans*-1,2-cyclohexanediamine (12 µL, 0.1 mmol, 20 mol%) and anhydrous degassed THF (2 mL) were then added. The reaction vessel was sealed and submitted to microwave irradiation (250 W, temperature up to 200 °C, stabilised to 15-19 bar) for two hours. After cooling down, the crude mixture was filtered through a celite plug (eluted with CH_2Cl_2) and the solvents were removed. The crude product was adsorbed onto silica gel and purified by column chromatography (SiO_2, CH_2Cl_2/pentane gradient from 10:90 to 70:30) to give pure compound **5** as a white solid in 49% yield (126.9 mg, 0.24 mmol).

***R*$_f$** = 0.27 (SiO_2, CH_2Cl_2/hexane 20:80). **m.p.:** 303 °C. **^{1}H NMR** (500 MHz, CD_2Cl_2, 25 °C): δ = 8.80 (d, ^3J = 9.0 Hz, 1H, H_{11} or H_{14}), 8.76 (d, ^3J = 8.0 Hz, 1H, H_{11} or H_{14}), 7.66 – 7.57 (m, 3H, H_8, H_{17} and H_{10} or H_{15}), 7.53 (virt. t, ^3J = 8.4 Hz, 1H, H_{10} or H_{15}), 7.41 – 7.37 (m, 2H, H_{22B}), 7.37 – 7.32 (m, 3H, H_{23B} and H_{24B}), 7.32 – 7.27 (m, 6H, H_3, H_9 and H_{16}), 7.23 – 7.15 (m, 6H, H_4 and H_5), 7.05 (t, ^3J = 7.4 Hz, 1H, H_{24A}), 6.93 (virt. t, ^3J = 7.7 Hz, 2H, H_{23A}), 6.56 (dd, ^3J = 8.3 Hz, ^4J = 1.3 Hz, 2H, H_{22A}) ppm. **^{13}C{^{1}H} NMR** (126 MHz, CD_2Cl_2, 25 °C): δ = 157.2 (C^{20A}), 147.6 (C^6 or C^{19}), 141.8 (C^{20B}), 139.0 (C^2), 138.4 (C^{21B}), 138.1 (C^6 or C^{19}), 136.4 (C^{21A}), 131.9 (C^{12} or C^{13}), 131.0 (C^{22A}), 130.6 (C^{22B}), 130.5 (C^{12} or C^{13}), 129.9 (C^3), 128.7 (C^7 or C^{18}), 128.6 (C^{23B}), 128.3 (C^4), 128.0 (C^7 or C^{18}), 127.7 (C^{24B}), 127.3 (C^{23A}), 127.2 (C^{24A}), 127.2 (C^5), 126.4 (C^8, C^9, C^{16} and C^{17}), 125.9 (C^{10} and C^{15}), 123.7 (C^{11}

and C^{14}), 70.8 (C^{1}) ppm. **UV-Vis** (CH_2Cl_2): λ_{max} (ε)= 252 (41200), 281 (24100), 291 (sh, 18400), 348 nm (9300 $mol^{-1}dm^3cm^{-1}$). **HR-MS** (DCI-CH_4): calcd. for $C_{41}H_{28}$ $[M]^+$: 520.2191, found 520.2180. **Crystal data:** see section VII of the SI.

17,17-Diphenylcyclopenta[*l*,*l'*]diphenanthrene (6a):

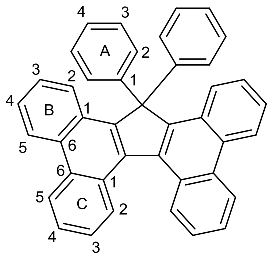

In a dry Schlenk tube, hexaphenylcyclopentadiene **1a** (50 mg, 0.09 mmol, 1.0 eq.) was dissolved in anhydrous dichloromethane (14 mL) and the resulting solution was degassed by bubbling argon for 15 minutes. A solution of iron(III) chloride $FeCl_3$ (465 mg, 2.87 mmol, 30.0 eq.) in degassed anhydrous nitromethane (10 mL) was then added dropwise *via* cannula, inducing a colour change from pale-yellow to dark purple. The reaction was stirred for 1 hour at room temperature, while continuously degassing with an argon flow. Ethanol was then added to quench the reaction and the medium was diluted with dichloromethane, washed with water (twice) and finally with brine. The organic layer was dried over magnesium sulfate, filtered and the solvents were removed *in vacuo*. The crude product was partially purified by a first column chromatography (SiO_2, chloroform/pentane 20:80) and a second column chromatography (SiO_2, chloroform/pentane 15:85) afforded pure 17,17-diphenylcyclopenta[*l*,*l'*]diphenanthrene **6a** as a pale-yellow solid in 32% yield (16 mg, 0.03 mmol).

***R*$_f$**= 0.43 (SiO_2, CH_2Cl_2/hexane 20:80). **m.p.:** 289 °C. **^{1}H NMR** (500 MHz, CD_2Cl_2, 25 °C): δ = 8.79 (dt, ^3J = 8.2 Hz, ^4J = 0.7 Hz, 2H, H_{C5}), 8.77 (dt, ^3J = 8.3 Hz, ^4J = 0.7 Hz, 2H, H_{B5}), 8.67 (dd, ^3J = 8.1 Hz, ^4J = 0.8 Hz, 2H, H_{C2}), 7.81 (dd, ^3J = 8.3 Hz, ^4J = 0.7 Hz, 2H, H_{B2}), 7.75 (ddd, ^3J = 8.3 Hz, ^3J = 6.9 Hz, ^4J = 1.4 Hz, 2H, H_{C4}), 7.68 (ddd, ^3J = 8.3 Hz, ^3J = 6.9 Hz, ^4J = 1.4 Hz, 2H, H_{C3}), 7.64-7.60 (m, 4H, H_{A2}), 7.58 (ddd, ^3J = 8.3 Hz, ^3J = 6.9 Hz, ^4J = 1.4 Hz, 2H, H_{B4}), 7.41 (ddd, ^3J = 8.3 Hz, ^3J = 6.9 Hz, ^4J = 1.4 Hz, 2H, H_{B3}), 7.20-7.14 (m, 6H, H_{A3} and H_{A4}) ppm. **^{13}C{^{1}H} NMR** (126 MHz, CD_2Cl_2, 25 °C): δ = 150.0 ($C^{Cp\text{-}B}$), 140.8 (C^{A1}), 136.1 ($C^{Cp\text{-}C}$), 132.1 (C^{C6}), 131.7 (C^{B6}), 129.2 (C^{A2}), 128.9 (C^{A3}), 128.5 (C^{C1}), 128.2 (C^{C2}), 127.7 (C^{B1}), 127.5 (C^{A4}), 126.8 (C^{C4}), 126.5 (C^{B3}), 126.3 (C^{B4}), 126.0 (C^{B2}), 125.9 (C^{C3}), 123.9 (C^{B5} and C^{C5}), 69.3 ($C^{Cp\text{-}A}$) ppm. **UV-Vis** (CH_2Cl_2): λ_{max} (ε)= 256 (43000), 293 (21200), 305 (29600), 371 (10000), 389 nm (9700 $mol^{-1}dm^3cm^{-1}$). **Fluorescence** (CH_2Cl_2): λ_{ex} = 300 nm, λ_{em} = 410, 425 nm. **HR-MS** (DCI-CH_4): calcd. for $C_{41}H_{26}$ $[M]^+$: 518.2035, found 518.2048.

3,6,11,14-Tetra(*tert*-butyl)-17,17-di(4'-*tert*-butylphenyl)cyclopenta[*l*,*l'*]diphenanthrene (6b):

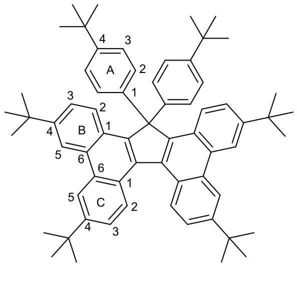

In a dry Schlenk tube, hexa(4'-*tert*-butylphenyl)cyclopenta-1,3-diene **1b** (50 mg, 0.058 mmol, 1.0 eq.) was dissolved in anhydrous dichloromethane (12 mL) and the resulting solution was degassed by bubbling argon for 15 minutes. A solution of iron(III) chloride $FeCl_3$ (170 mg, 1.05 mmol, 18.0 eq.) in degassed anhydrous nitromethane (10 mL) was then added dropwise *via* cannula, inducing a colour change from pale-yellow to dark purple. The reaction was stirred for 1 hour at room temperature, while continuously degassing with an argon flow. Ethanol was then added to quench the reaction and the medium was diluted with dichloromethane, washed with water (twice) and finally with brine. The organic layer was dried over magnesium sulfate, filtered and the solvents were removed *in vacuo*. The crude product was purified by

column chromatography (SiO_2, CH_2Cl_2/pentane 10:90) to give pure cyclopenta[*l*,*l'*]diphenanthrene **6b** as a yellow solid in 72% yield (36 mg, 0.042 mmol).

***R*f**= 0.50 (SiO_2, CH_2Cl_2/hexane 10:90). **m.p.**: 305 °C. **¹H NMR** (500 MHz, CD_2Cl_2, 25 °C): δ = 8.78 (br. s, 4H, H_{B5} and H_{C5}), 8.64 (d, ^3J = 8.7 Hz, 2H, H_{C2}), 7.75 (dd, ^3J = 8.7 Hz, ^4J = 2.0 Hz, 2H, H_{C3}), 7.72 (d, ^3J = 8.7 Hz, 2H, H_{B2}), 7.51 (AA'BB' pattern, ^3J = 8.8 Hz, 4H, H_{A2}), 7.48 (dd, ^3J = 8.7 Hz, ^4J = 2.0 Hz, 2H, H_{B3}), 7.20 (AA'BB' pattern, ^3J = 8.7 Hz, 4H, H_{A3}), 1.56 (s, 18H, $H_{tBu\text{-}C}$), 1.49 (s, 18H, $H_{tBu\text{-}B}$), 1.22 (s, 18H, $H_{tBu\text{-}A}$) ppm. **^{13}C{^{1}H} NMR** (126 MHz, CD_2Cl_2, 25 °C): δ = 149.9 (C^{A4}), 149.3 ($C^{Cp\text{-}B}$), 149.1 (C^{C4}), 148.5 (C^{B4}), 138.3 (C^{A1}), 135.4 ($C^{Cp\text{-}C}$), 131.8 (C^{B6} or C^{C6}), 131.5 (C^{B6} or C^{C6}), 128.9 (C^{A2}), 128.0 (C^{C2}), 126.6 (C^{B1} or C^{C1}), 125.9 (C^{B1} or C^{C1}), 125.8 (C^{B2}), 125.7 (C^{A3}), 124.5 (C^{B3}), 123.9 (C^{C3}), 119.6 (C^{B5} or C^{C5}), 119.5 (C^{B5} or C^{C5}), 68.3 ($C^{Cp\text{-}A}$), 35.5 ($C^{quat\text{-}tBu\text{-}B}$ or $C^{quat\text{-}tBu\text{-}C}$), 35.4 ($C^{quat\text{-}tBu\text{-}B}$ or $C^{quat\text{-}tBu\text{-}C}$), 34.6 ($C^{quat\text{-}tBu\text{-}A}$), 31.7 ($C^{tBu\text{-}B}$ or $C^{tBu\text{-}C}$), 31.6 ($C^{tBu\text{-}B}$ or $C^{tBu\text{-}C}$), 31.4 ($C^{tBu\text{-}A}$) ppm. **UV-Vis** (CH_2Cl_2): λ_{max} (ε)= 259 (118400), 296 (58600), 310 (77600), 378 (30500), 397 nm (30100 $mol^{-1}dm^3cm^{-1}$). **Fluorescence** (CH_2Cl_2): λ_{ex} = 300 nm, λ_{em} = 416, 436 nm. **HR-MS** (DCI-CH_4): calcd. for $C_{65}H_{74}$ $[M]^+$: 854.5791, found 854.5801. **Crystal data:** see section VII of the SI.

3,6,11,14-Tetramethyl-17,17-di(4'-methylphenyl)cyclopenta[*l*,*l'*]diphenanthrene (6c):

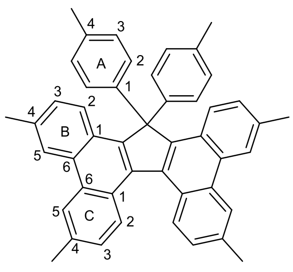

In a dry Schlenk tube, hexa(4-tolyl)cyclopentadiene **1c** (40 mg, 0.07 mmol, 1.0 eq.) was dissolved in anhydrous dichloromethane (13 mL) and the resulting solution was degassed by bubbling argon for 15 minutes. A solution of iron(III) chloride $FeCl_3$ (192 mg, 1.19 mmol, 18.0 eq.) in degassed anhydrous nitromethane (9 mL) was then added dropwise *via* cannula, inducing a colour change from pale-yellow to dark purple. The reaction was stirred for 1 hour at room temperature, while continuously degassing with an argon flow. Ethanol was then added to quench the reaction and the medium was diluted with dichloromethane, washed with water (twice) and finally with brine. The organic layer was dried over magnesium sulfate, filtered and the solvents were removed *in vacuo*. The crude product was purified by column chromatography (SiO_2, CH_2Cl_2/pentane 15:85) to give pure cyclopenta[*l*,*l'*]diphenanthrene **6c** as a pale-yellow solid in 61% yield (24 mg, 0.04 mol).

***R*f**= 0.25 (SiO_2, CH_2Cl_2/hexane 10:90). **m.p.** > 410 °C. **¹H NMR** (500 MHz, CD_2Cl_2, 25 °C): δ = 8.56 (br. s, 2H, H_{B5} or H_{C5}), 8.54 (br. s, 2H, H_{B5} or H_{C5}), 8.53 (d, ^3J = 8.3 Hz, 2H, H_{C2}), 7.68 (d, ^3J = 8.4 Hz, 2H, H_{B2}), 7.49-7.44 (m, 6H, H_{A2} and H_{C3}), 7.21 (dd, ^3J = 8.5 Hz, ^4J = 1.4 Hz, 2H, H_{B3}), 6.97 (d, ^3J = 8.1 Hz, 4H, H_{A3}), 2.67 (s, 6H, $H_{Me\text{-}C}$), 2.55 (s, 6H, $H_{Me\text{-}B}$), 2.20 (s, 6H, $H_{Me\text{-}A}$) ppm. **^{13}C{^{1}H} NMR** (126 MHz, CD_2Cl_2, 25 °C): δ = 149.2 ($C^{Cp\text{-}B}$), 138.2 (C^{A1}), 137.0 (C^{A4}), 136.2 (C^{C4}), 135.7 (C^{B4}), 135.2 ($C^{Cp\text{-}C}$), 131.8 (C^{C6}), 131.4 (C^{B6}), 129.4 (C^{A3}), 129.1 (C^{A2}), 128.1 (C^{B3} or C^{C2}), 127.9 (C^{B3} or C^{C2}), 127.3 (C^{C3}), 126.6 (C^{C1}), 125.8 (C^{B1}), 125.8 (C^{B2}), 123.7 (C^{B5} or C^{C5}), 123.6 (C^{B5} or C^{C5}), 68.3 ($C^{Cp\text{-}A}$), 22.1 ($C^{Me\text{-}B}$ or $C^{Me\text{-}C}$), 22.0 ($C^{Me\text{-}B}$ or $C^{Me\text{-}C}$), 21.0 ($C^{Me\text{-}A}$) ppm. **UV-Vis** (CH_2Cl_2): λ_{max} (ε)= 261 (64500), 296 (31400), 310 (41300), 378 (16300), 397 nm (16900 $mol^{-1}dm^3cm^{-1}$). **Fluorescence** (CH_2Cl_2): λ_{ex} = 300 nm, λ_{em} = 413, 435 nm. **HR-MS** (DCI-CH_4): calcd. for $C_{47}H_{38}$ $[M]^+$: 602.2974, found 602.2990. **Crystal data:** see section VII of the SI.

5-(4'-Fluorophenyl)-1,2,3,4,5-pentaphenylcyclopenta-1,3-diene (7):
(obtained as a 73:18:9 mixture of regioisomers)

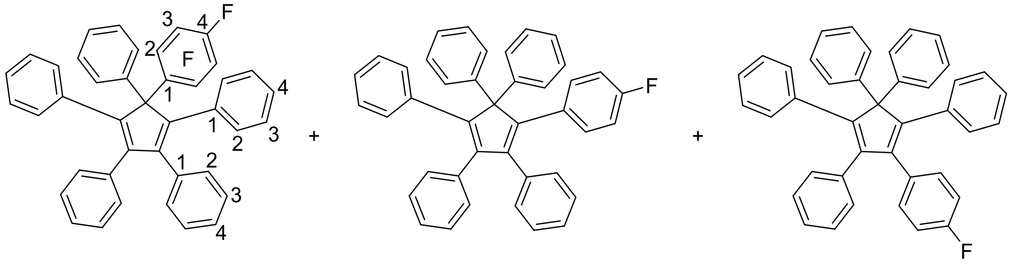

5-(4'-Fluorophenyl)-1,2,3,4,5-pentaphenylcyclopenta-1,3-diene was synthesised using 1,2,3,4,5-pentaphenylcyclopenta-1,3-diene as starting material. Pentaphenylcyclopentadiene **2a** (223 mg, 0.5 mmol, 1.0 eq.) was placed in a 10 mL microwave reactor equipped with a magnetic stir bar in a glovebox. Cesium carbonate (489 mg, 1.5 mmol, 3.0 eq.), 1-fluoro-4-iodobenzene (137 µL, 1.5 mmol, 3.0 eq.), copper(I) iodide (9.5 mg, 0.05 mmol, 10 mol%), (±)-*trans*-1,2-cyclohexanediamine (12 µL, 0.1 mmol, 20 mol%) and anhydrous degassed THF (2 mL) were then added. The reaction vessel was sealed and submitted to microwave irradiation (250 W available, temperature up to 200 °C, pressure stabilised to 15-19 bar) for two hours. After cooling down, the crude mixture was filtered through a celite plug (eluted with CH_2Cl_2) and the solvents were removed. The crude product was then adsorbed onto silica gel and purified by column chromatography (SiO_2, CH_2Cl_2/pentane gradient from 10:90 to 70:30) to give compound **7** as a white solid in 47% yield (125 mg, 0.23 mmol), as a 73:18:9 mixture of regioisomers.

$\boldsymbol{R_f}$ = 0.32 (SiO_2, CH_2Cl_2/hexane 20:80). **m.p.**: 101 °C. **^{1}H NMR** (500 MHz, CD_2Cl_2, 25 °C): δ = 7.47 – 7.39 (m, 3.96H), 7.24 – 7.16 (m, 3.72H), 7.15 – 7.07 (m, 5.69H), 7.05 – 6.95 (m, 5.78H), 6.94 – 6.86 (m, 5.12H), 6.80 (d, ^3J = 8.8 Hz, 0.26H), 6.77 – 6.70 (m, 3.99H), 6.60 (t, ^3J = 9.0 Hz, 0.47H) ppm. **^{13}C{^{1}H} NMR** (126 MHz, CD_2Cl_2, 25 °C): δ = 162.1 (d, $^1J_{C\text{-}F}$ = 245.3 Hz, $C^{F4\text{-}regio3}$), 162.0 (d, $^1J_{C\text{-}F}$ = 244.8 Hz, $C^{F4\text{-}regio1}$), 161.8 (d, $^1J_{C\text{-}F}$ = 245.3 Hz, $C^{F4\text{-}regio2}$), 153.0 ($C^{quat\text{-}Cp\text{-}regio3}$), 153.0 ($C^{quat\text{-}Cp\text{-}regio1}$), 152.0 ($C^{quat\text{-}Cp\text{-}regio2}$), 144.1 ($C^{quat\text{-}Cp}$), 139.5 ($C^{quat\text{-}Cp}$), 136.4 ($C^1$), 136.2 ($C^1$), 136.1 ($C^1$), 135.4 (d, $^4J_{C\text{-}F}$ = 3.4 Hz, C^{F1}), 132.4 (d, $^3J_{C\text{-}F}$ = 8.1 Hz, $C^{F2\text{-}regio2}$), 132.3 (d, $^3J_{C\text{-}F}$ = 7.8 Hz, $C^{F2\text{-}regio3}$), 131.0 (d, $^3J_{C\text{-}F}$ = 7.9 Hz, $C^{F2\text{-}regio1}$), 130.8 ($C^{CH\text{-}Ar}$), 130.6 ($C^{CH\text{-}Ar}$), 129.4 ($C^{CH\text{-}Ar}$), 129.4 ($C^{CH\text{-}Ar}$), 129.3 ($C^{CH\text{-}Ar}$), 128.4 ($C^{CH\text{-}Ar}$), 128.3 ($C^{CH\text{-}Ar}$), 128.1 ($C^{CH\text{-}Ar}$), 127.6 ($C^{CH\text{-}Ar}$), 127.5 ($C^{CH\text{-}Ar}$), 127.1 ($C^{CH\text{-}Ar}$), 127.0 ($C^{CH\text{-}Ar}$), 126.9 ($C^{CH\text{-}Ar}$), 115.0 (d, $^2J_{C\text{-}F}$ = 20.3 Hz, $C^{F3\text{-}regio1}$ and $C^{F3\text{-}regio3}$), 114.4 (d, $^2J_{C\text{-}F}$ = 20.9 Hz, $C^{F3\text{-}regio2}$), 75.1 ($C^{quat\text{-}Cp}$), 74.4 ($C^{quat\text{-}Cp}$) ppm. Due to the complexity of the spectra obtained for this regioisomers mixture, ^{1}H and ^{13}C NMR spectra could not be fully assigned. **^{19}F{^{1}H} NMR** (376 MHz, CD_2Cl_2, 25 °C): δ = -115.9 (F_{regio3}), -116.0 (F_{regio2}), -116.8 (F_{regio1}) ppm. **^{19}F NMR** (471 MHz, CD_2Cl_2, 25 °C): δ = -115.9 (m, 0.09 F, F_{regio3}), -116.0 (m, 0.18 F, F_{regio2}), -116.9 (m, 0.73 F, F_{regio1}) ppm. **UV-Vis** (CH_2Cl_2): λ_{max} (ε)= 247 (11900), 276 (7900), 337 nm (3300 mol^{-1}dm^{3}cm^{-1}). **HR-MS** (DCI-CH_4): calcd. for $C_{41}H_{29}F$ $[M]^+$: 540.2253 found 540.2252. **Crystal data:** see section VII of the SI.

IV. NMR spectra of new compounds

1,2,3,4,5,5-Hexaphenylcyclopenta-1,3-diene (1a):

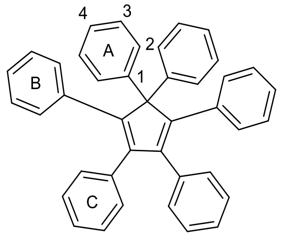

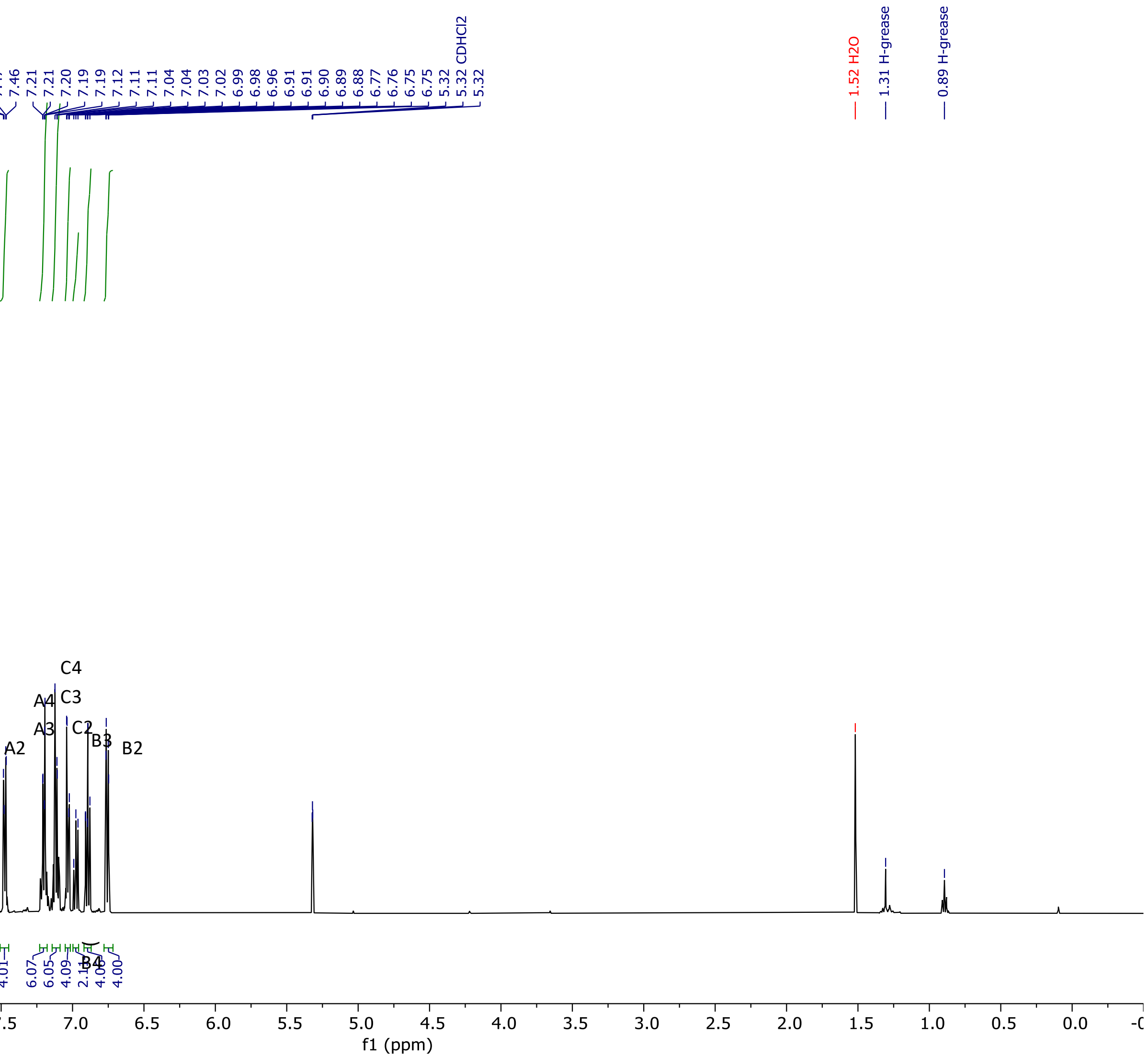

^{1}H-NMR of compound **1a** (500 MHz, CD_2Cl_2, 25 °C).

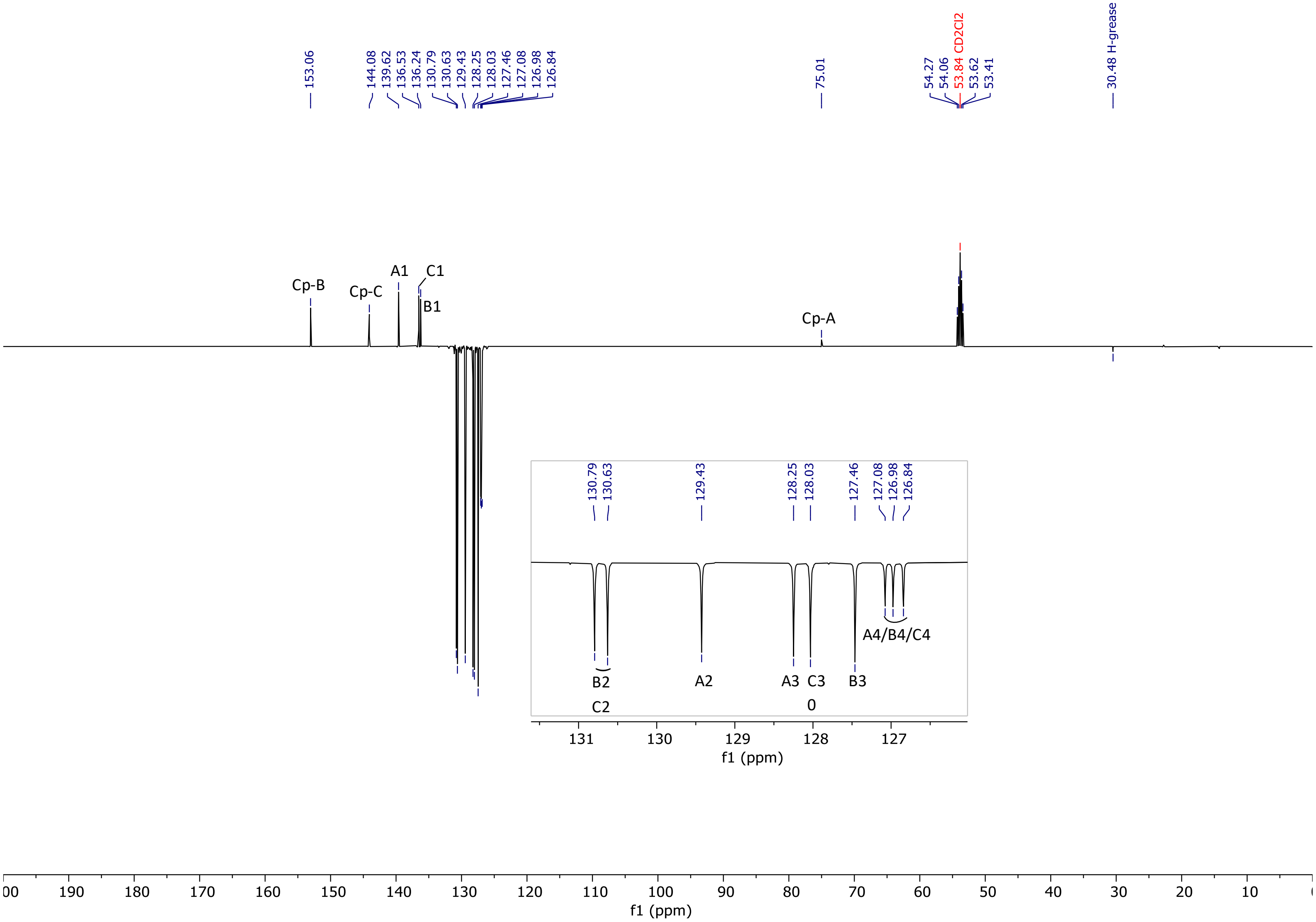

^{13}C-Jmod-NMR of compound **1a** (126 MHz, CD_2Cl_2, 25 °C).

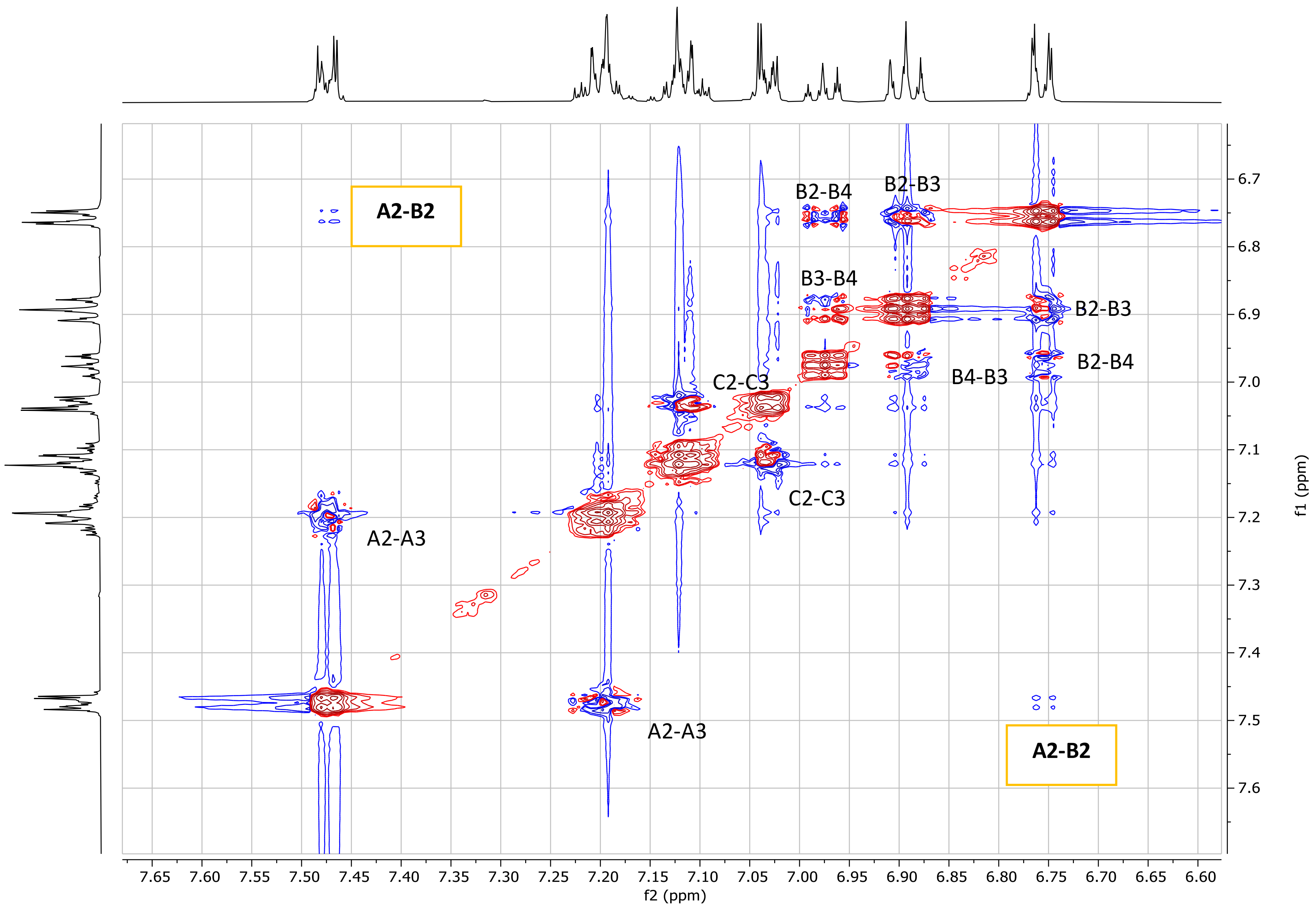

NOESY NMR of compound **1a** (500 MHz, CD_2Cl_2, 25 °C).

1,2,3,4,5,5-Hexa(4'-*tert*-butylphenyl)cyclopenta-1,3-diene (1b):

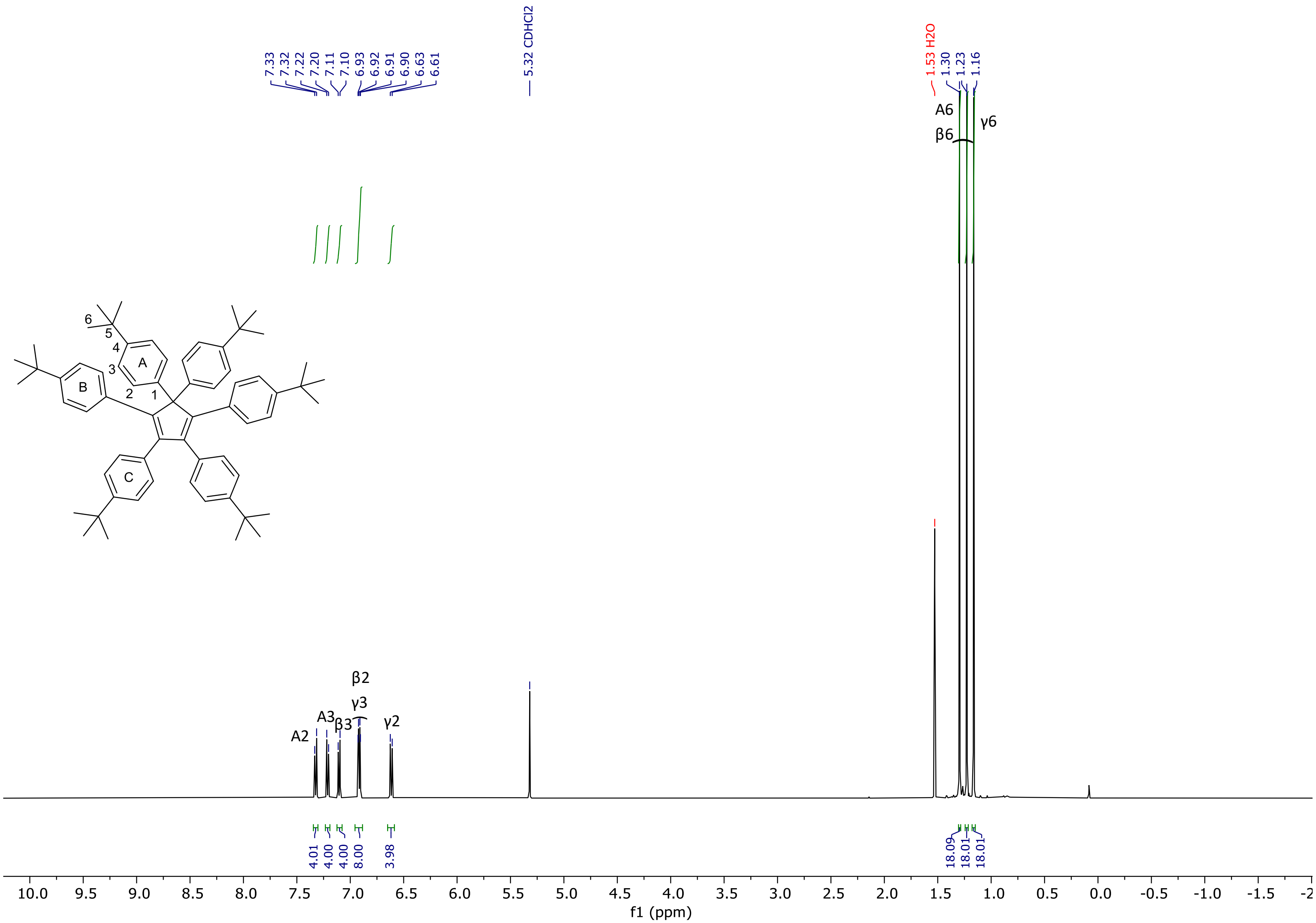

^{1}H-NMR of compound **1b** (500 MHz, CD_2Cl_2, 25 °C).

β and γ are protons from cycles B and C which could not be certainly differentiated.

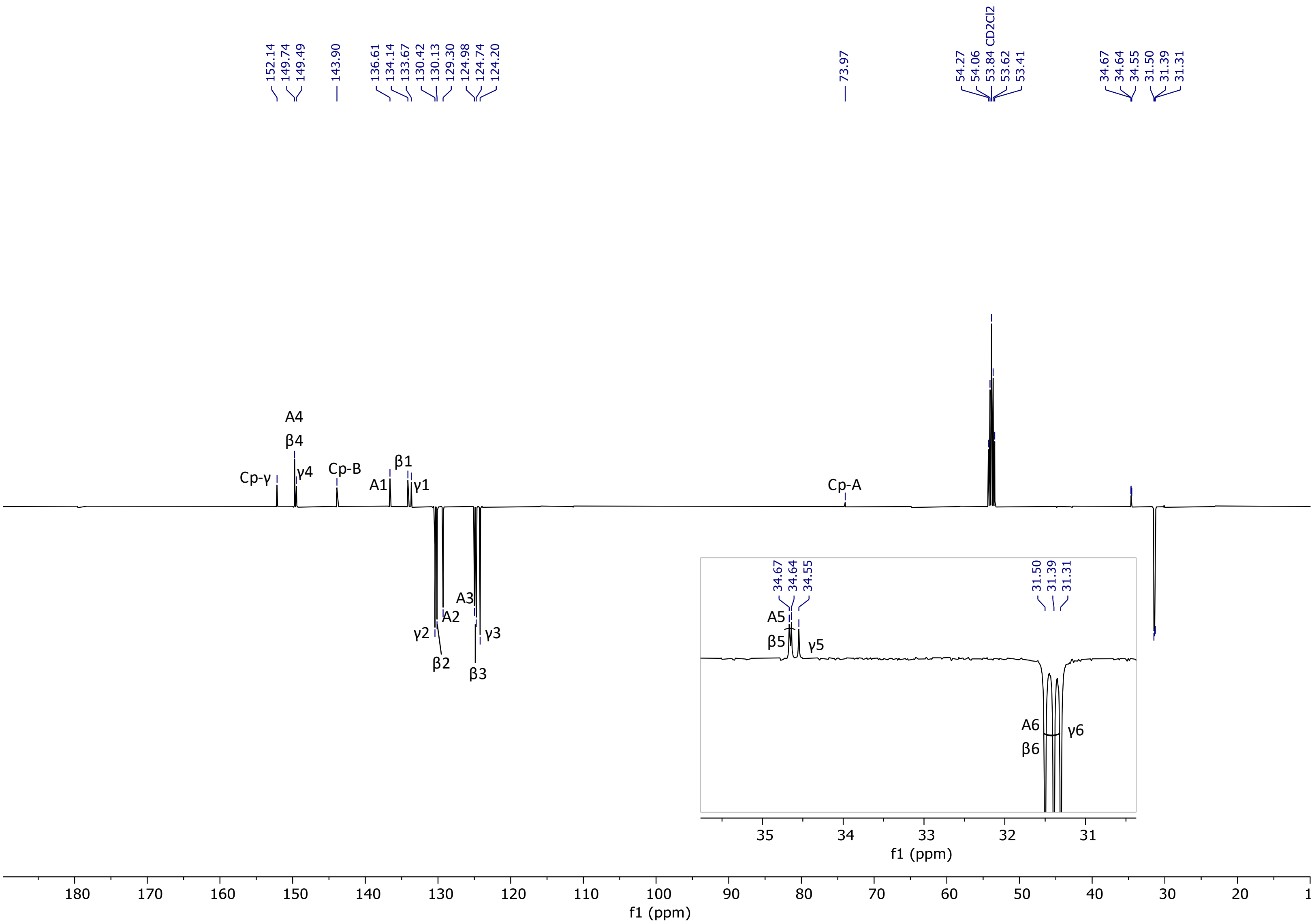

^{13}C-Jmod-NMR of compound **1b** (126 MHz, CD_2Cl_2, 25 °C).

β and γ are carbons from cycles B and C which could not be certainly differentiated.

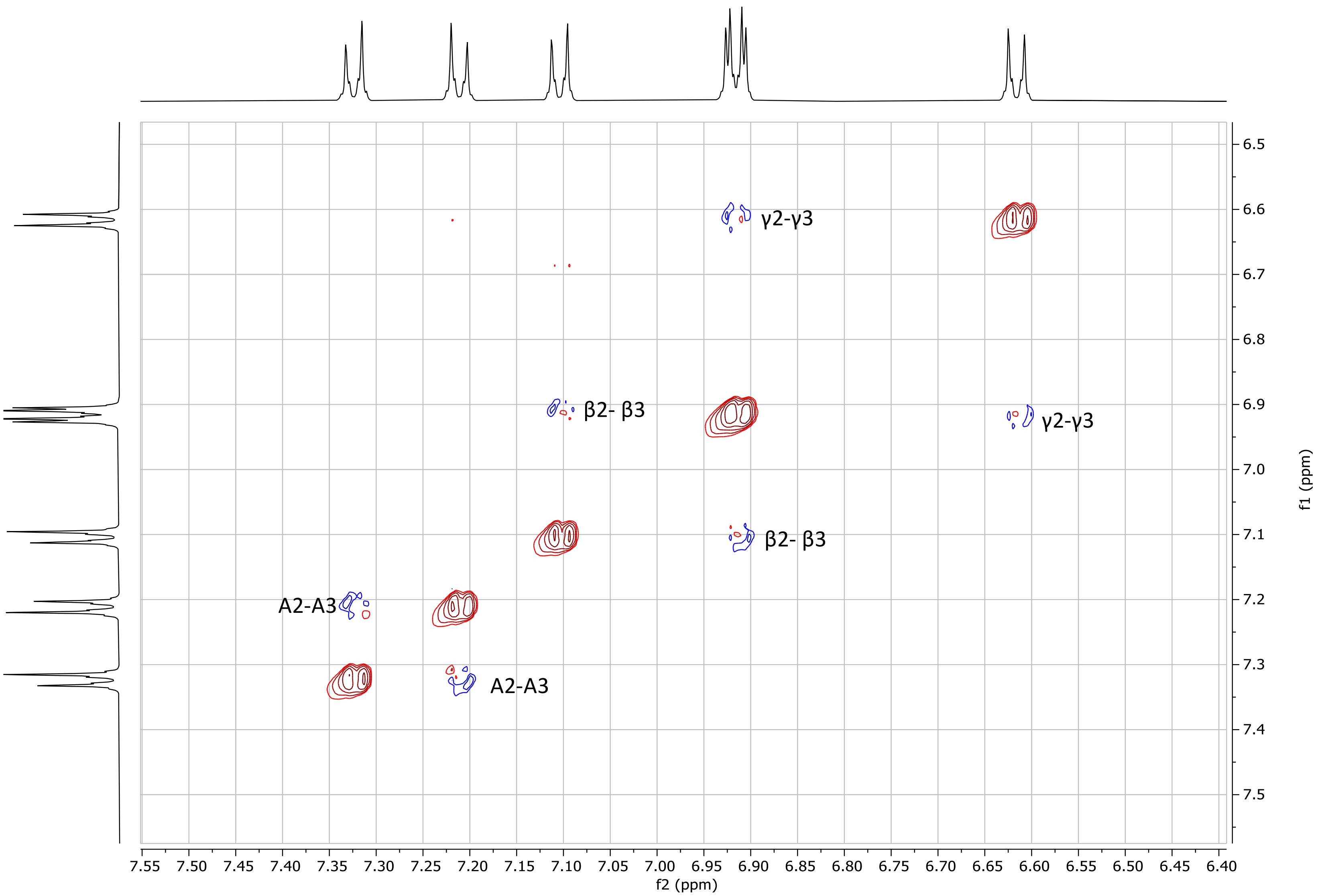

NOESY NMR of compound **1b** (500 MHz, CD_2Cl_2, 25 °C).

β and γ are carbons from cycles B and C which could not be certainly differentiated.

1,2,3,4,5,5-Hexa(4'-tolyl)cyclopenta-1,3-diene (1c):

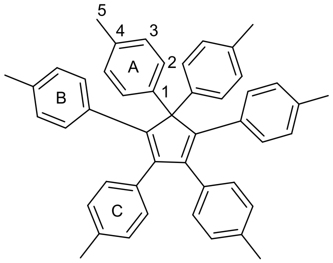

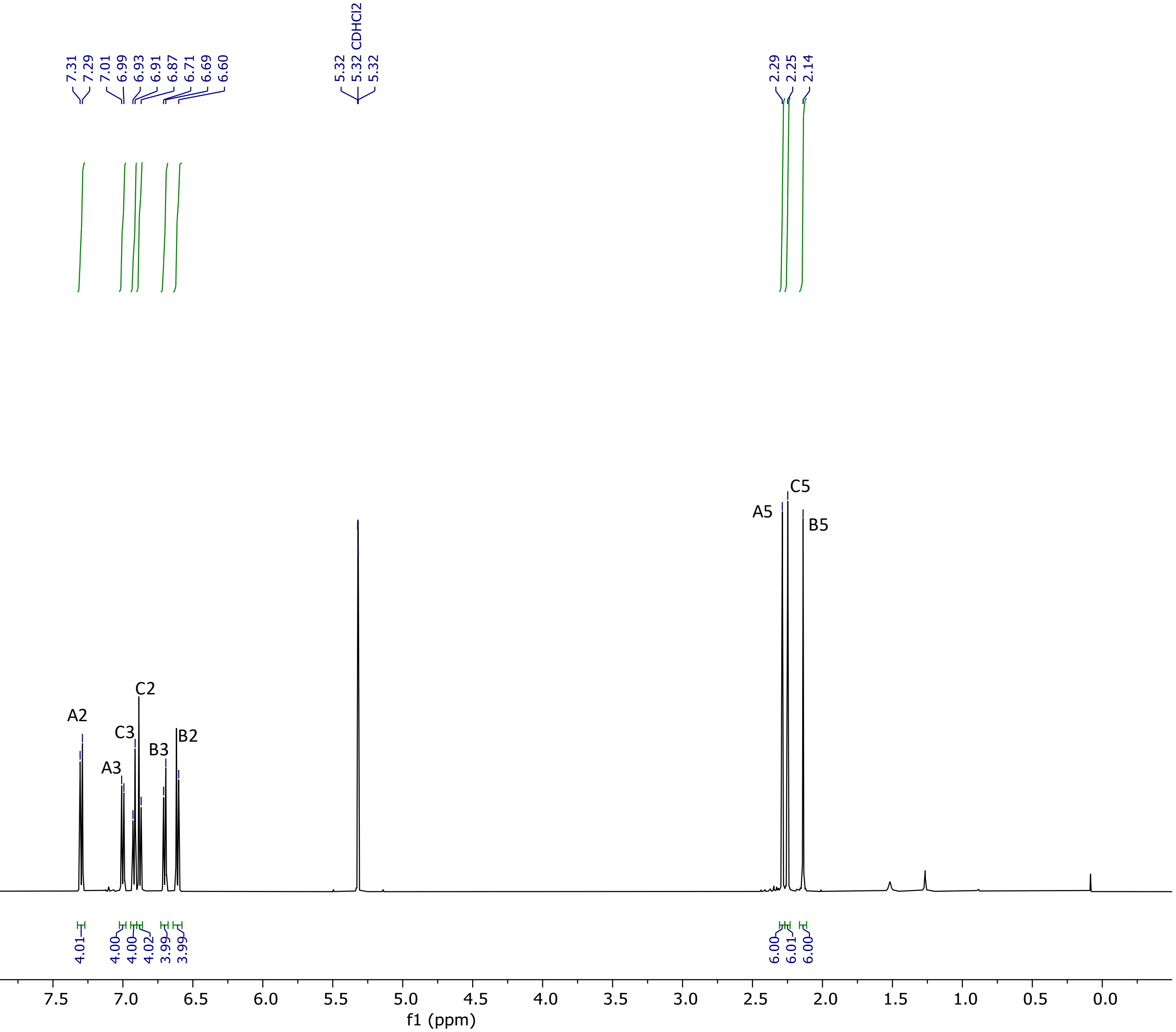

^{1}H-NMR of compound **1c** (500 MHz, CD_2Cl_2, 25 °C).

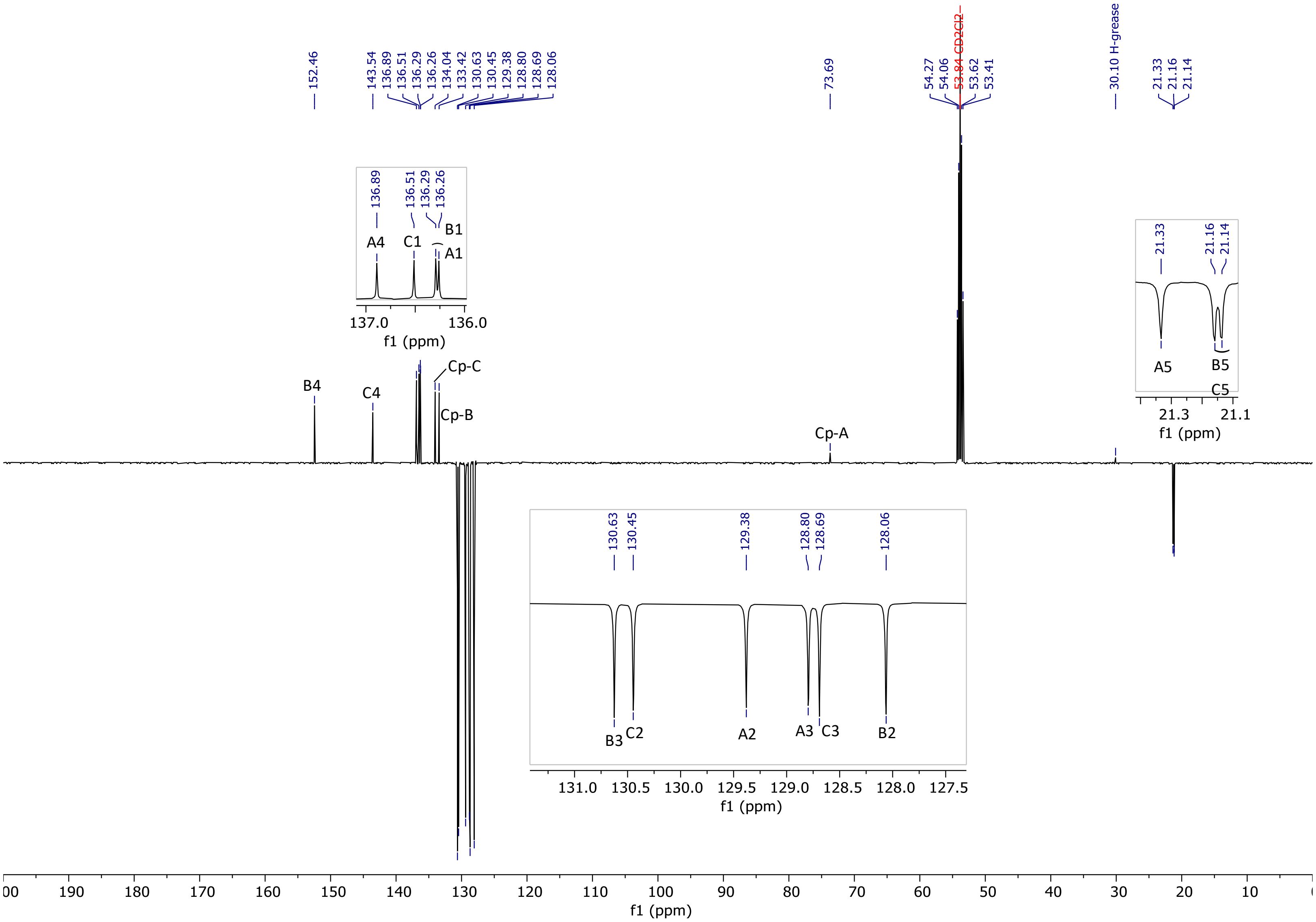

^{13}C-Jmod-NMR of compound **1c** (126 MHz, CD_2Cl_2, 25 °C).

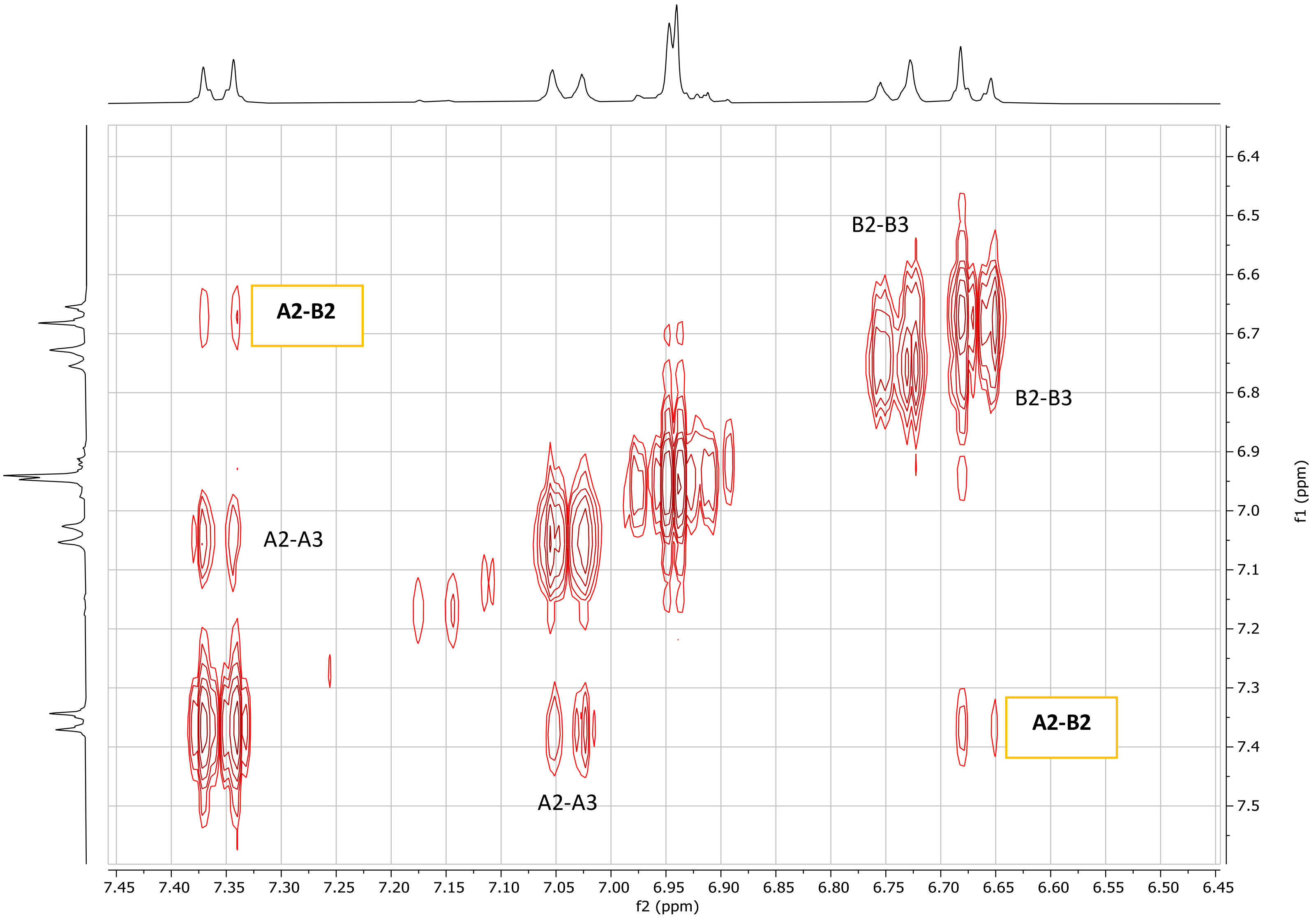

NOESY NMR of compound **1c** (300 MHz, CD_2Cl_2, 25 °C).

1,2,3,4,5,5-Hexa(3'-tolyl)cyclopenta-1,3-diene (1d):

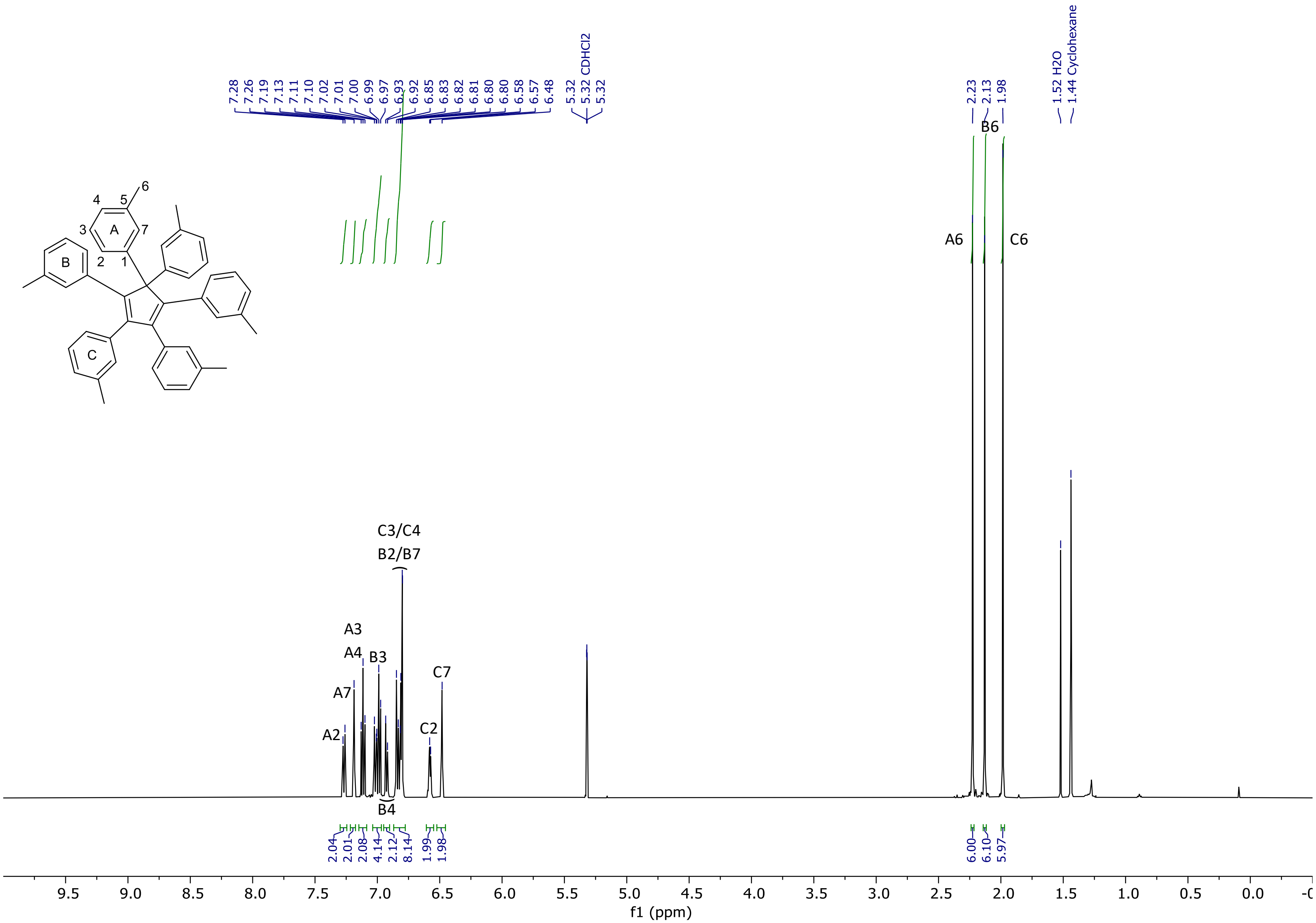

^{1}H-NMR of compound **1d** (500 MHz, CD_2Cl_2, 25 °C).

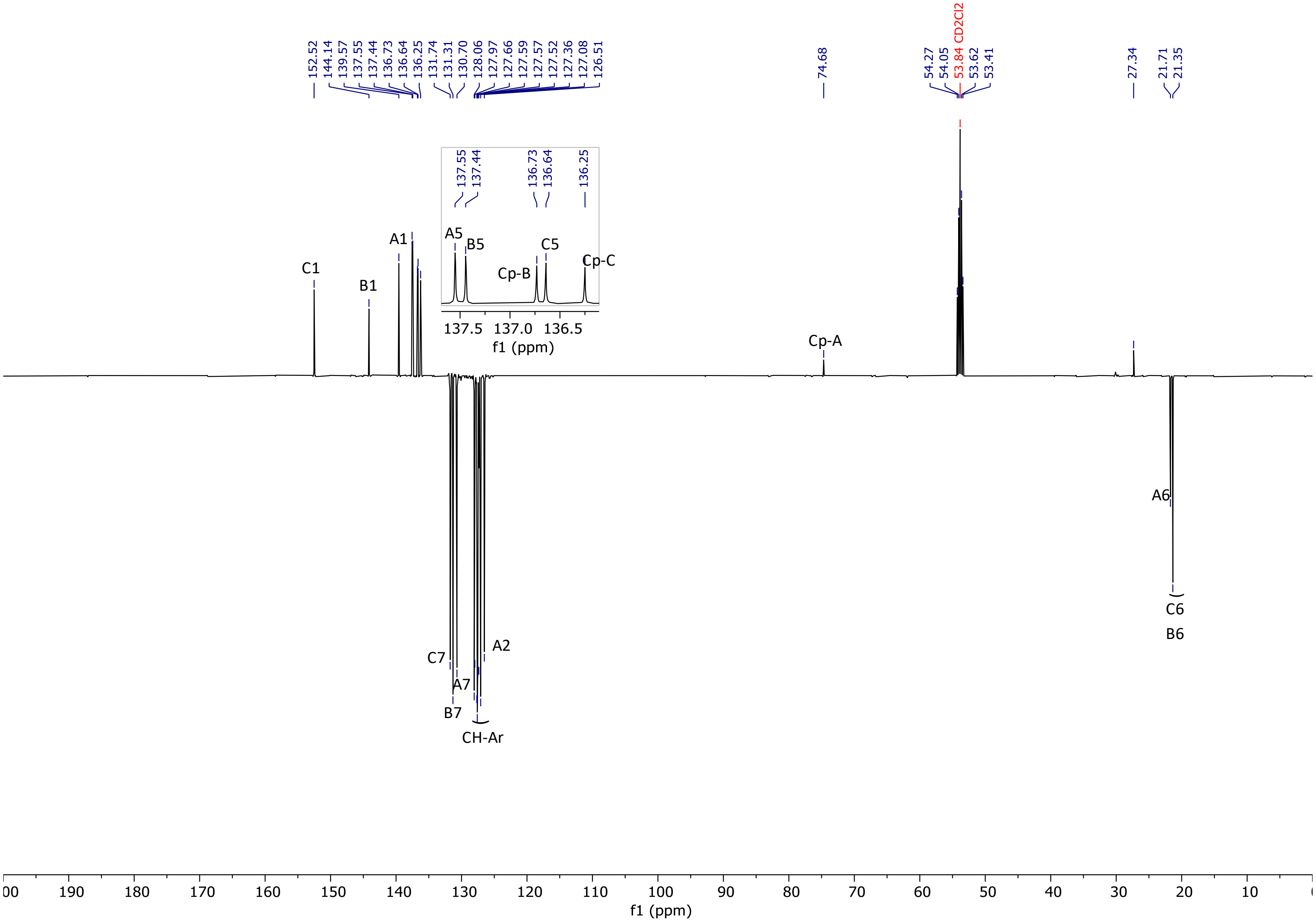

^{13}C-Jmod-NMR of compound **1d** (126 MHz, CD_2Cl_2, 25 °C).

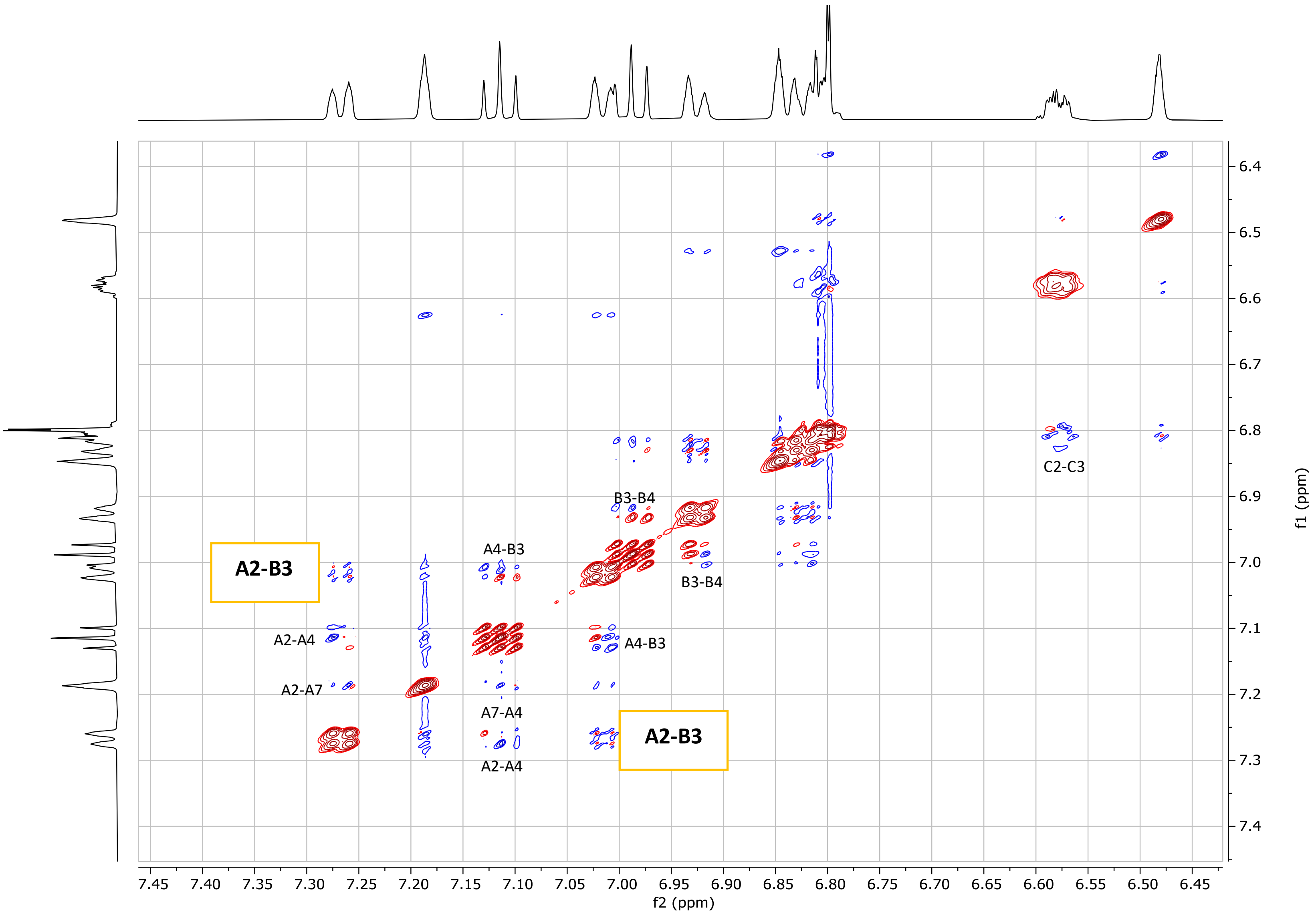

NOESY NMR of compound **1d** (500 MHz, CD_2Cl_2, 25 °C).

1,2,3,4,5,5-Hexa(naphth-2'-yl)cyclopenta-1,3-diene (1f):

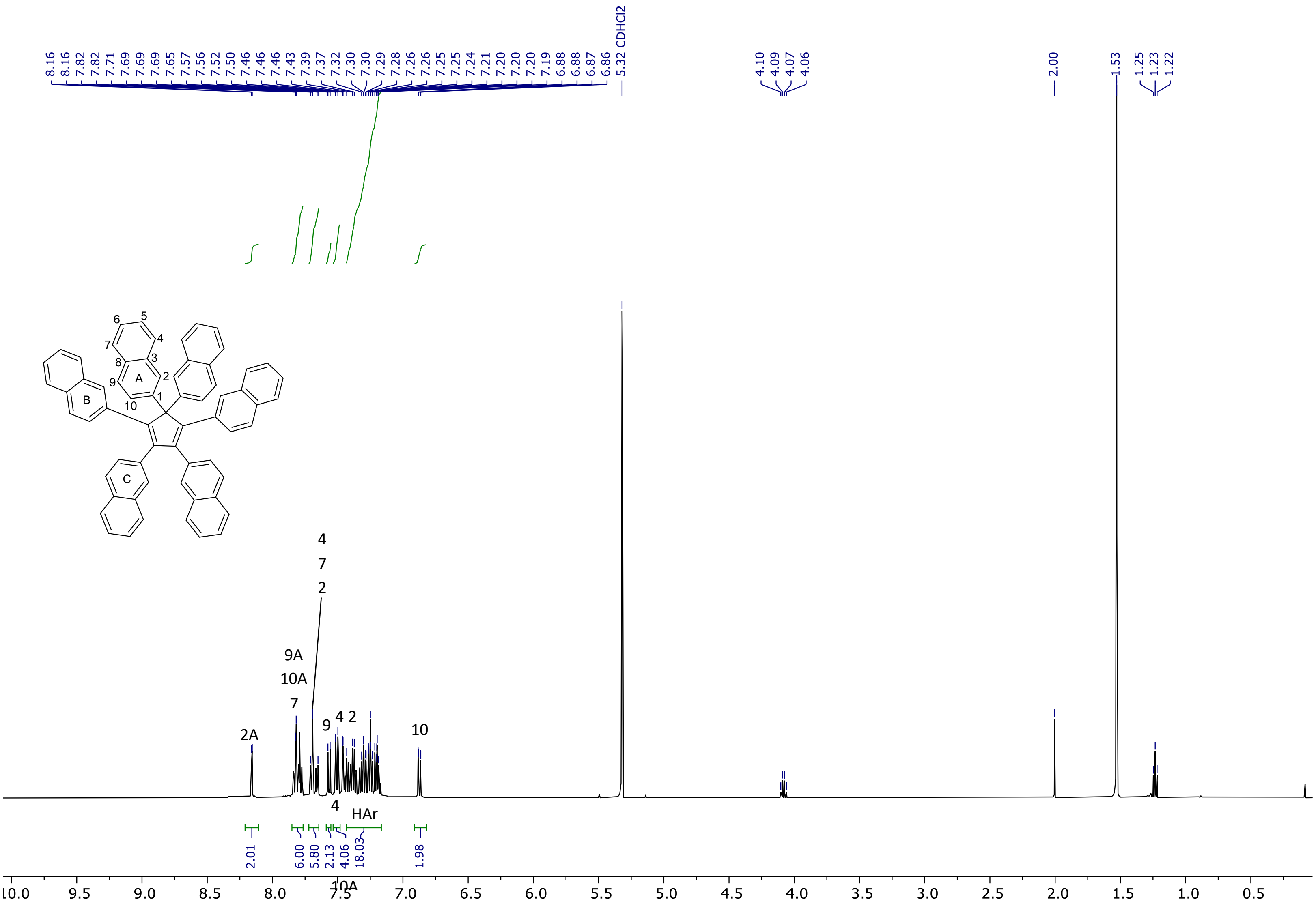

^{1}H-NMR of compound **1f** (500 MHz, CD_2Cl_2, 25 °C).

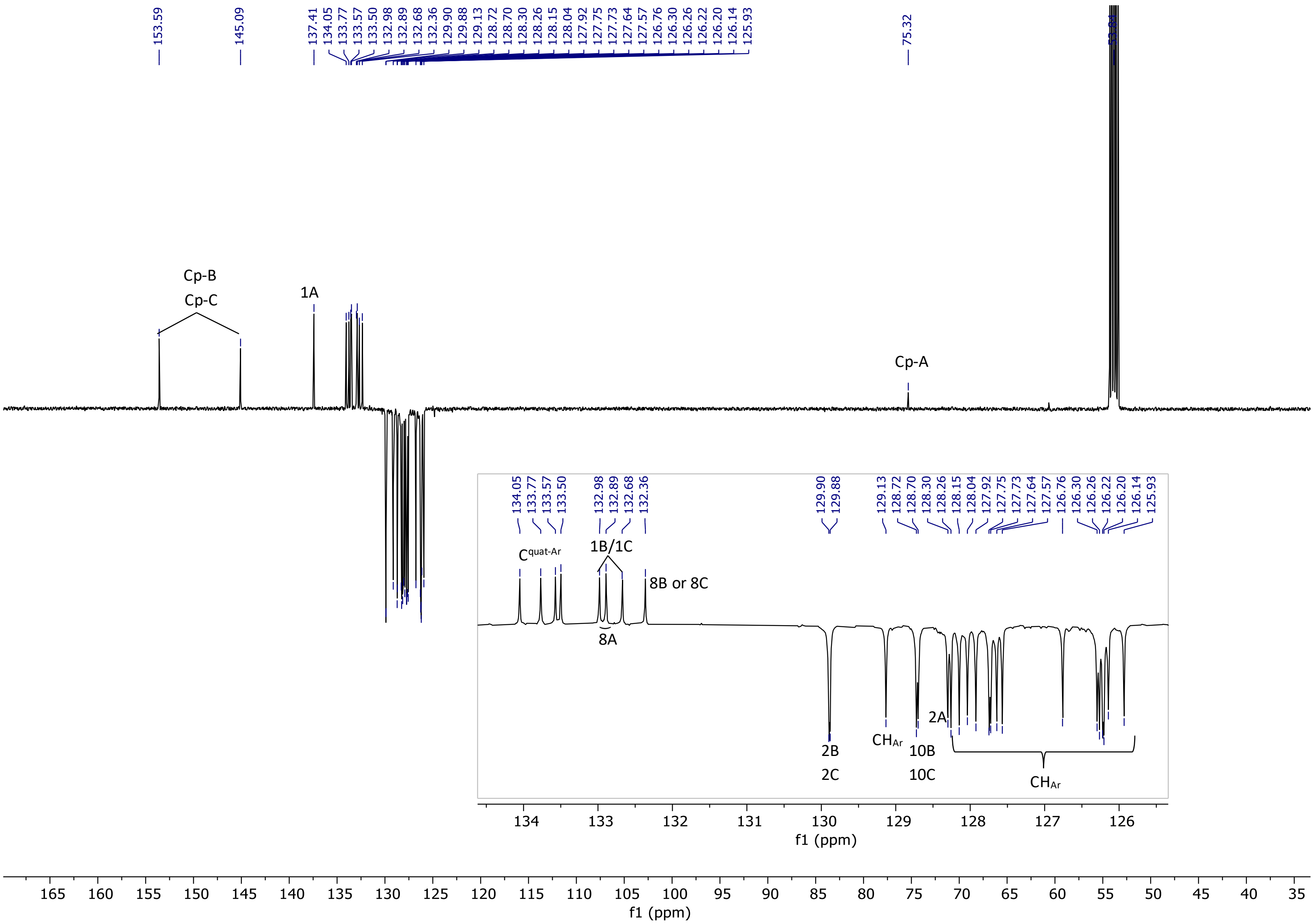

^{13}C-Jmod-NMR of compound **1f** (126 MHz, CD_2Cl_2, 25 °C).

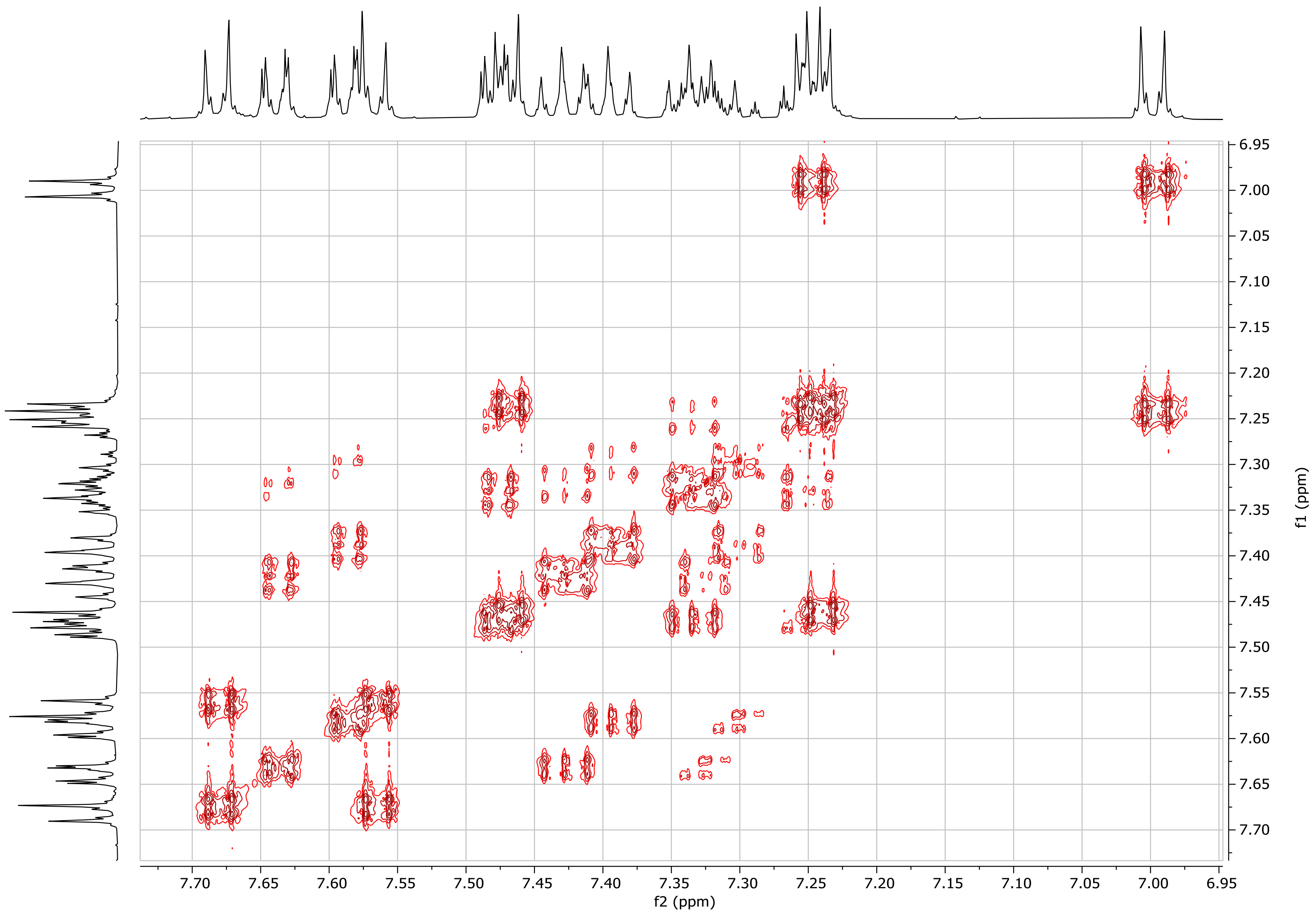

NOESY NMR of compound **1f** (500 MHz, CD_2Cl_2, 25 °C).

1,2,3,4,5,5-Hexa(*p*-biphenyl)cyclopenta-1,3-diene (1g):

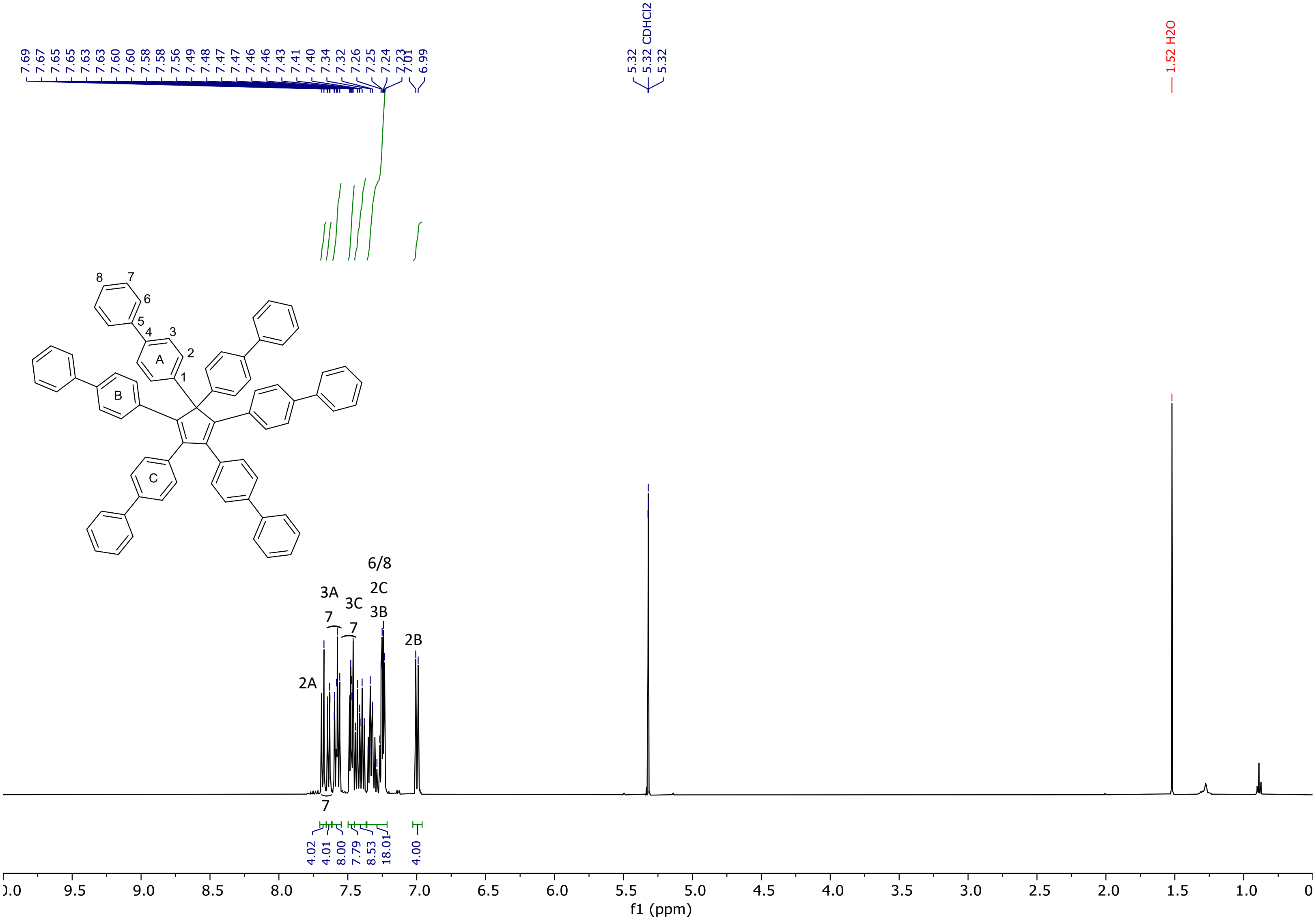

^{1}H-NMR of compound **1g** (500 MHz, CD_2Cl_2, 25 °C).

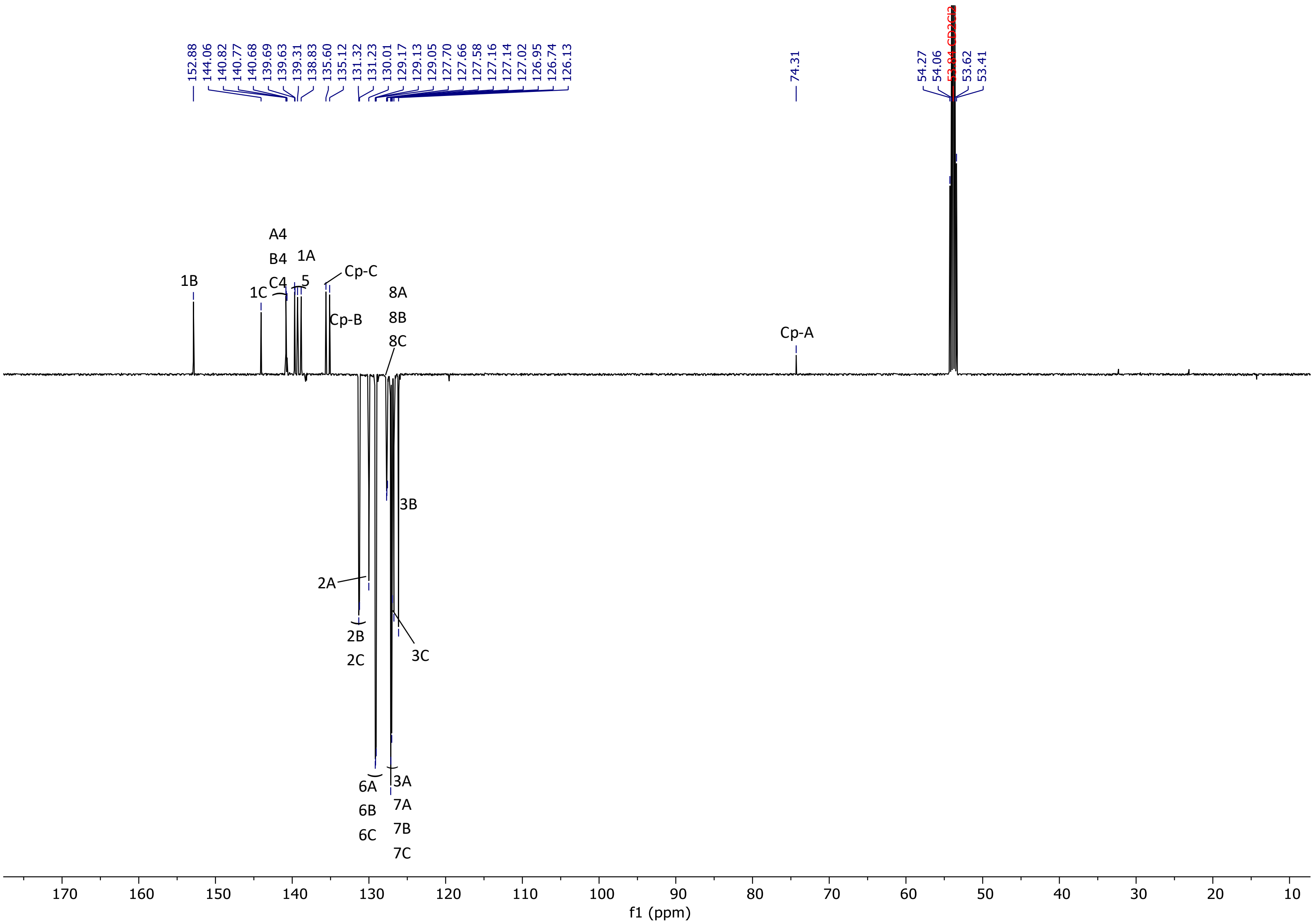

^{13}C-Jmod-NMR of compound **1g** (126 MHz, CD_2Cl_2, 25 °C).

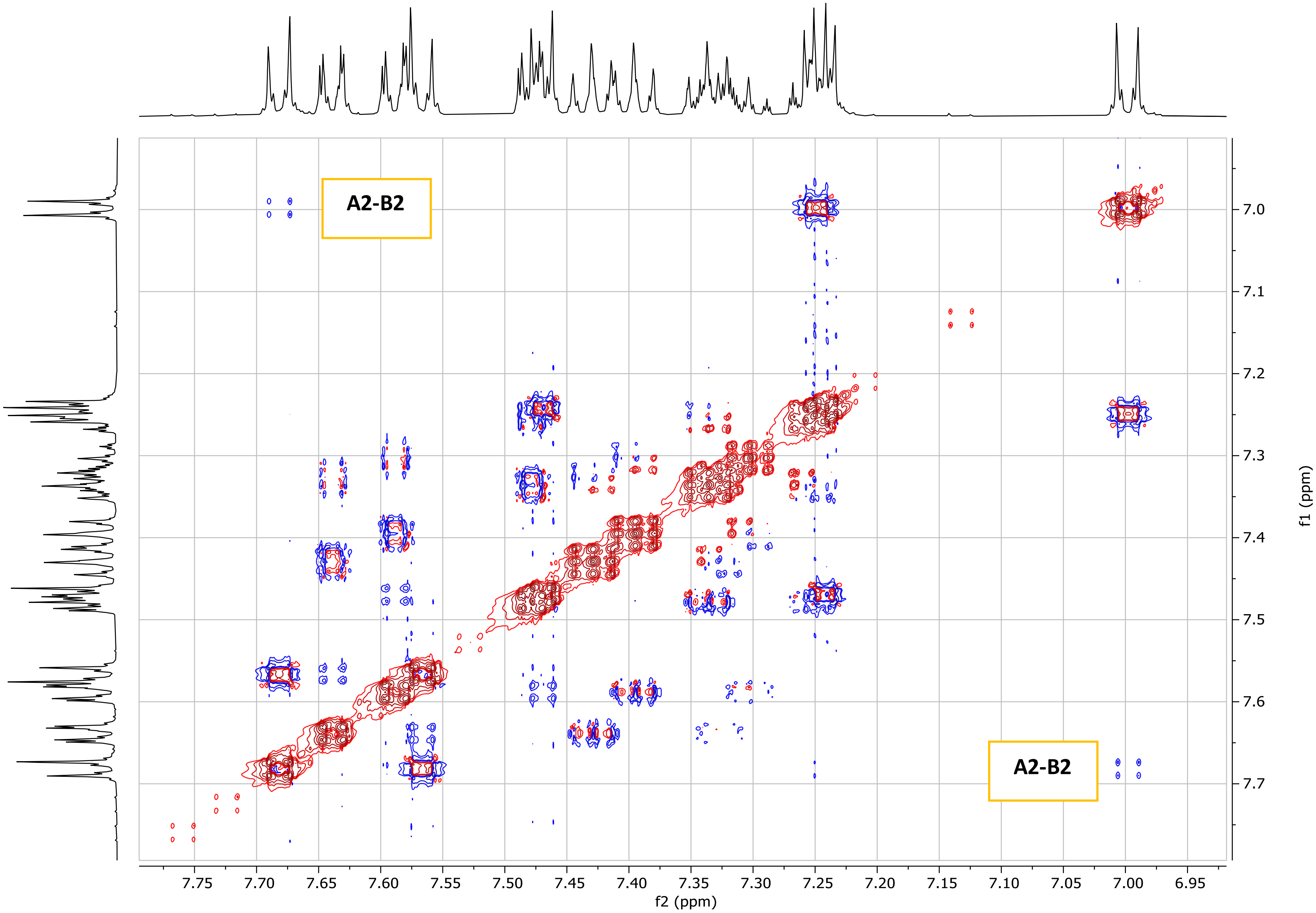

NOESY NMR of compound **1g** (500 MHz, CD_2Cl_2, 25 °C).

1,2,3,4,5,5-Hexa(4'-methoxyphenyl)cyclopenta-1,3-diene (1h):

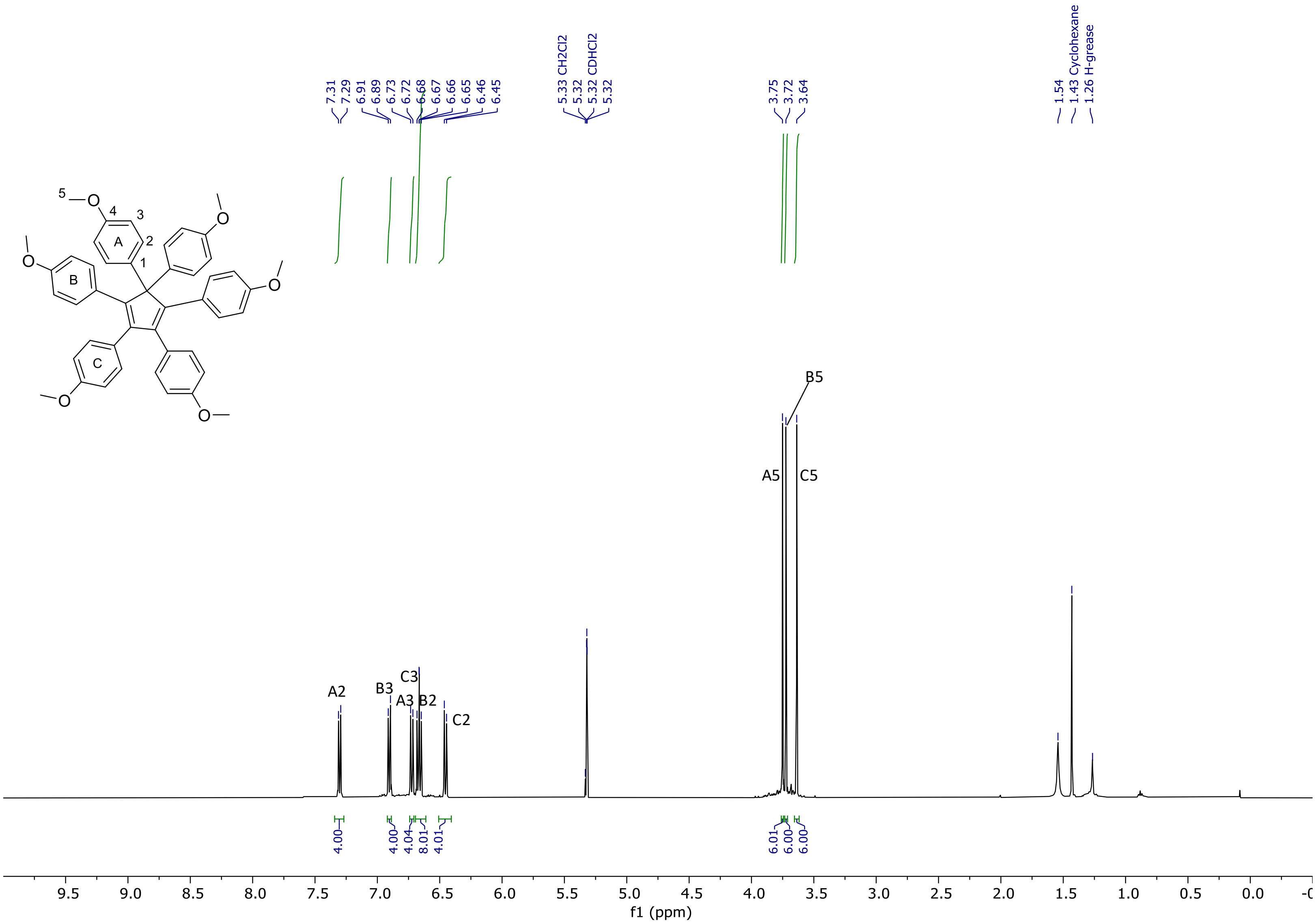

^{1}H-NMR of compound **1h** (500 MHz, CD_2Cl_2, 25 °C).

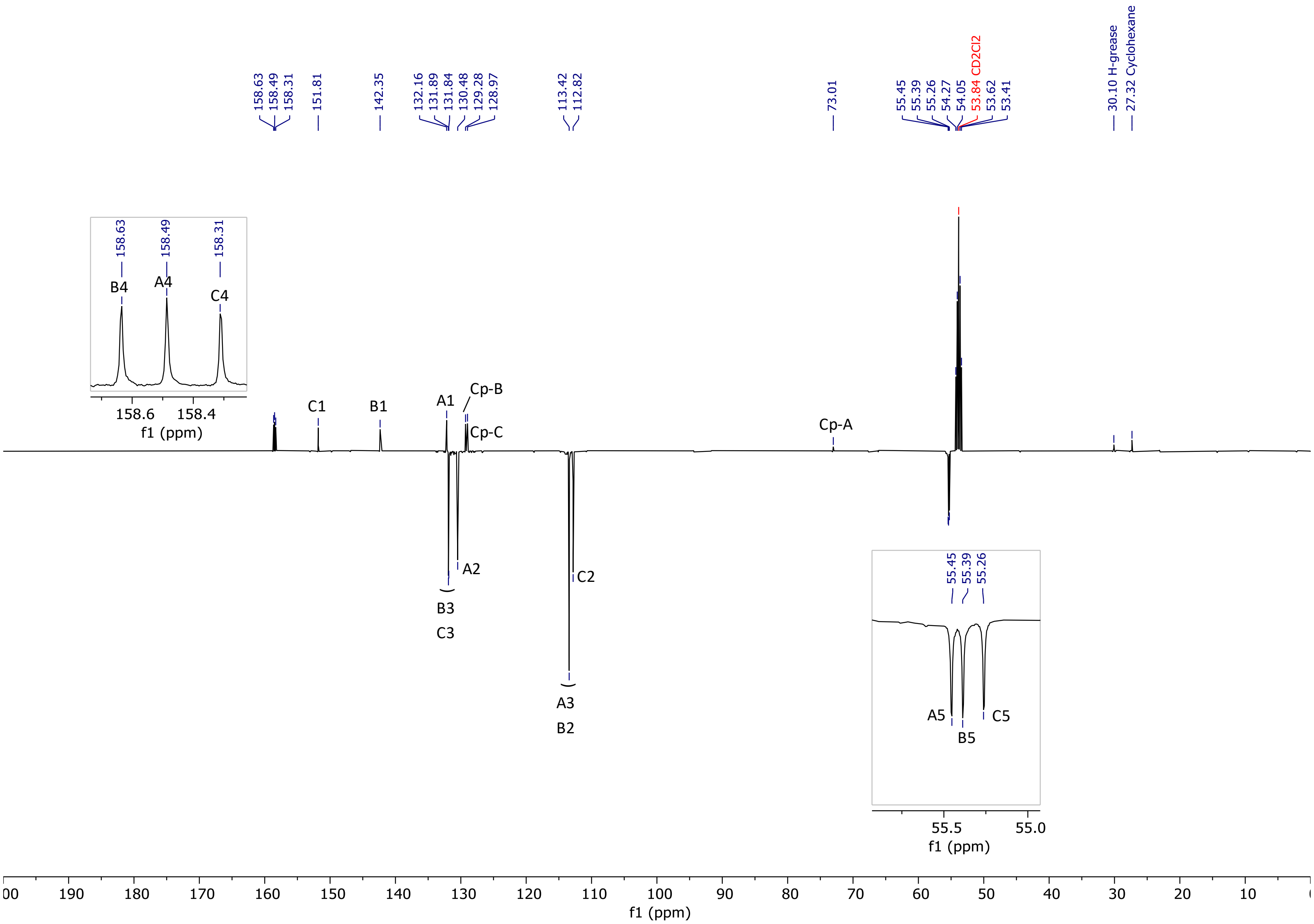

^{13}C-Jmod-NMR of compound **1h** (126 MHz, CD_2Cl_2, 25 °C).

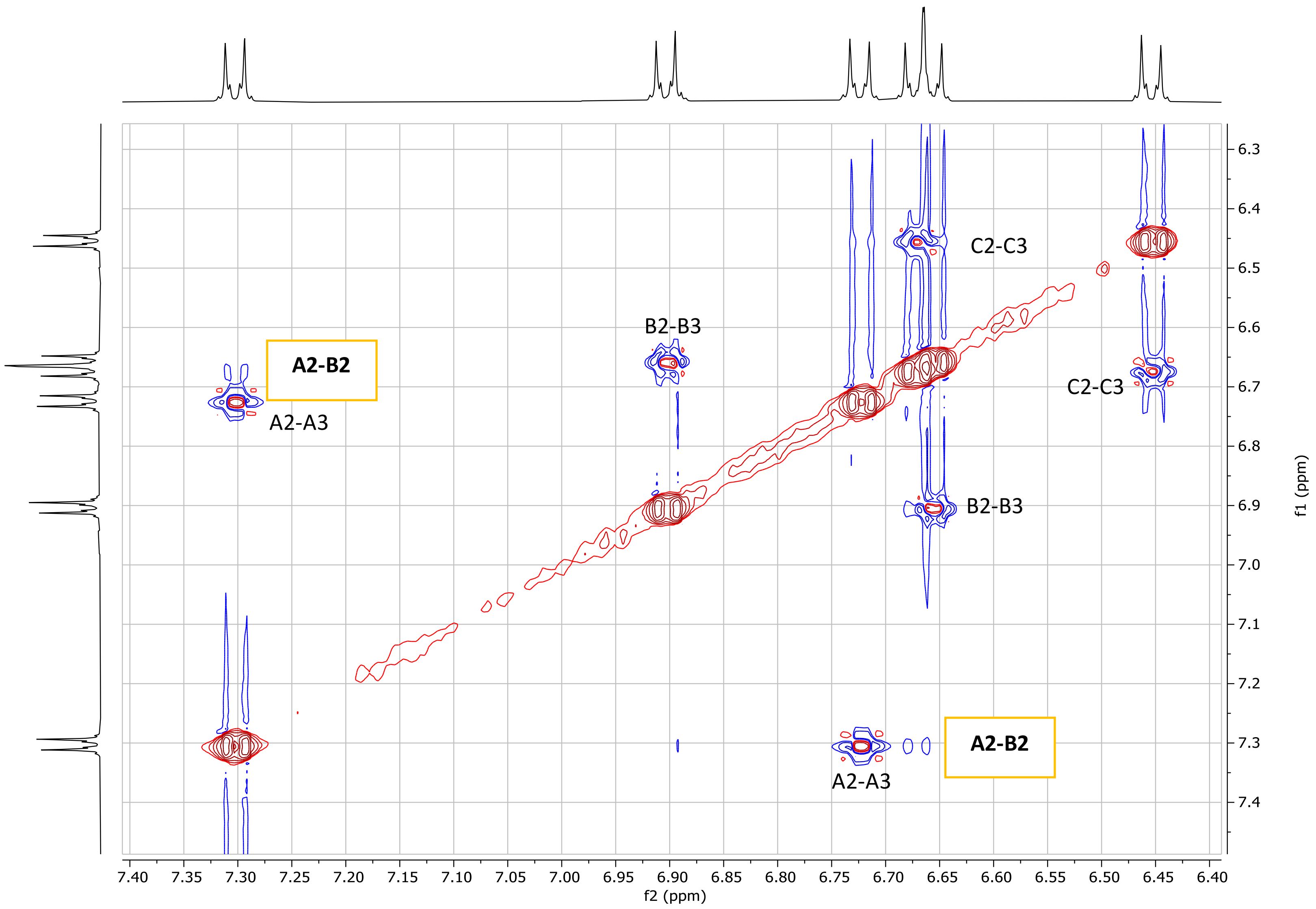

NOESY NMR of compound **1h** (500 MHz, CD_2Cl_2, 25 °C).

1,2,3,4,5,5-Hexa(3'-methoxyphenyl)cyclopenta-1,3-diene (1i):

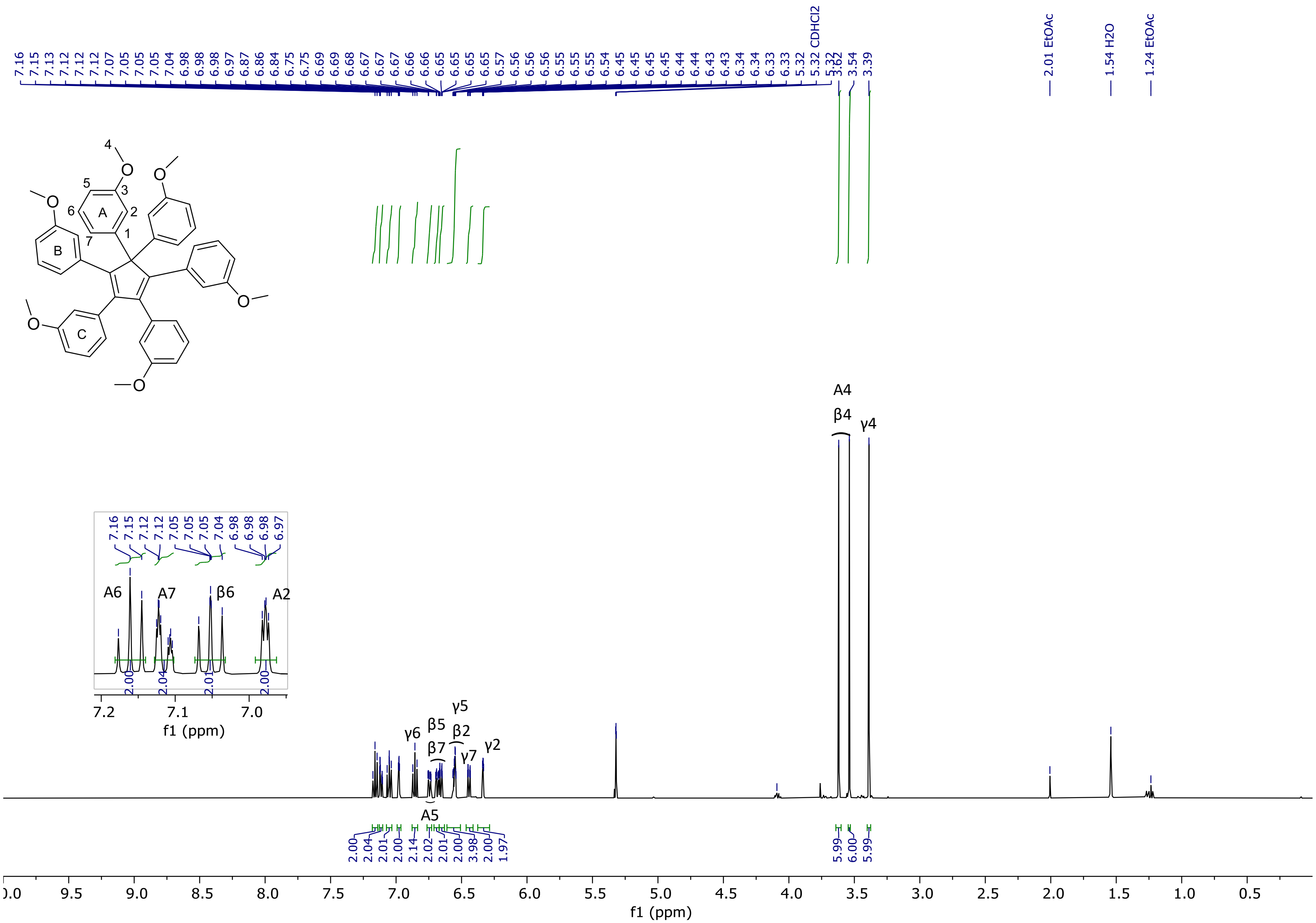

^{1}H-NMR of compound **1i** (500 MHz, CD_2Cl_2, 25 °C).

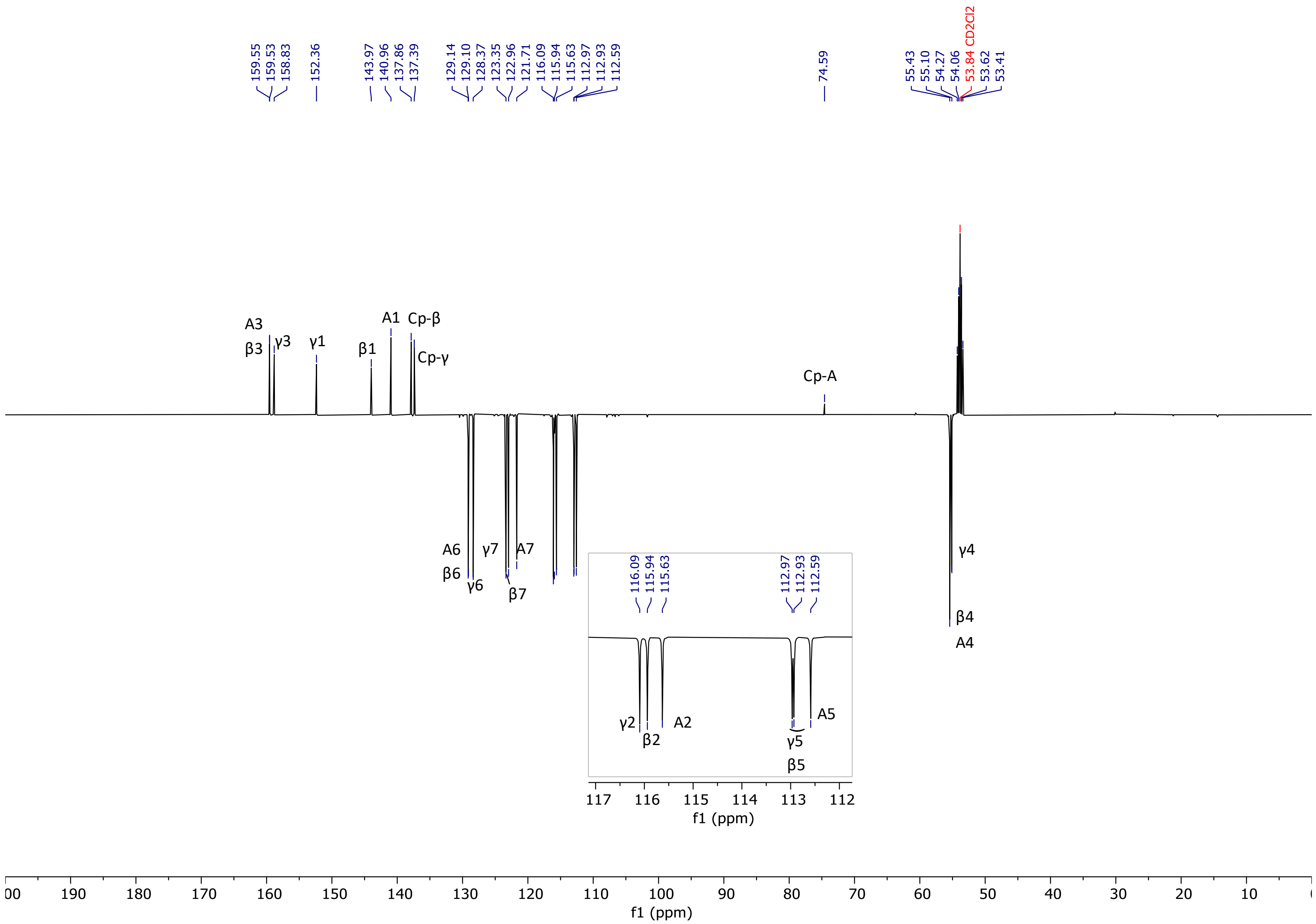

^{13}C-Jmod-NMR of compound **1i** (126 MHz, CD_2Cl_2, 25 °C).

1,2,3,4,5,5-Hexa(4'-fluorophenyl)cyclopenta-1,3-diene (1j):

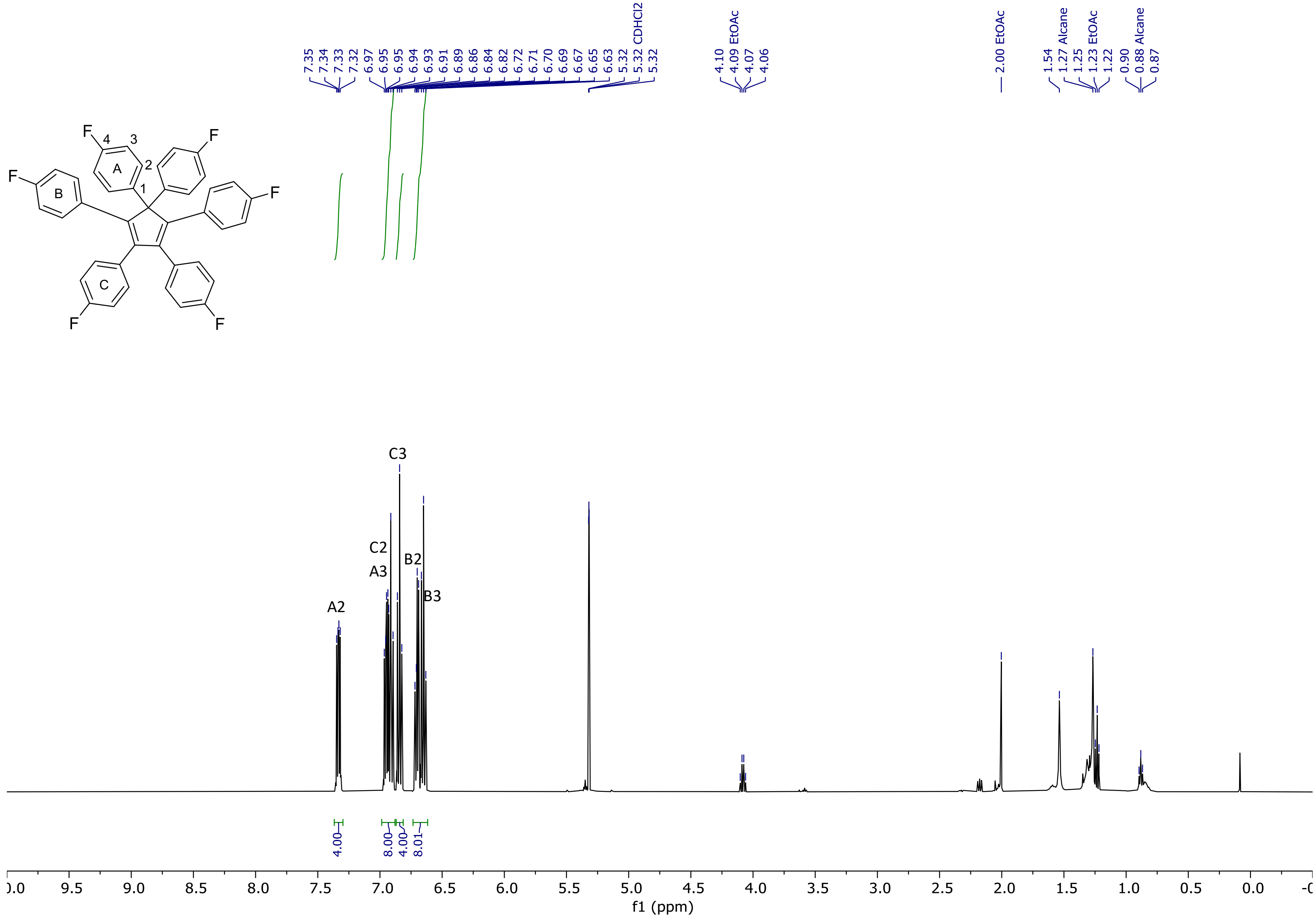

^{1}H-NMR of compound **1j** (500 MHz, CD_2Cl_2, 25 °C).

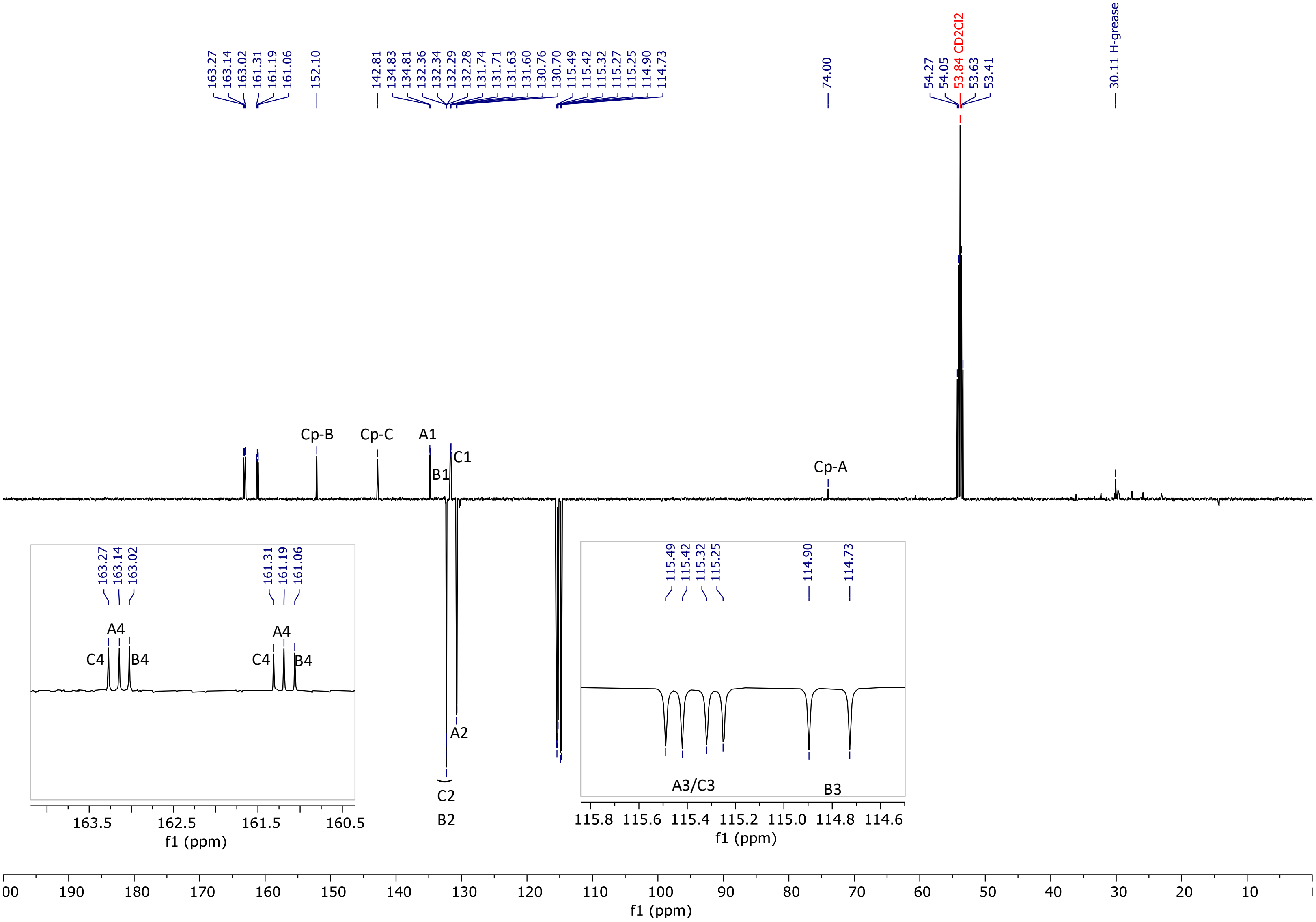

^{13}C-Jmod-NMR of compound **1j** (126 MHz, CD_2Cl_2, 25 °C).

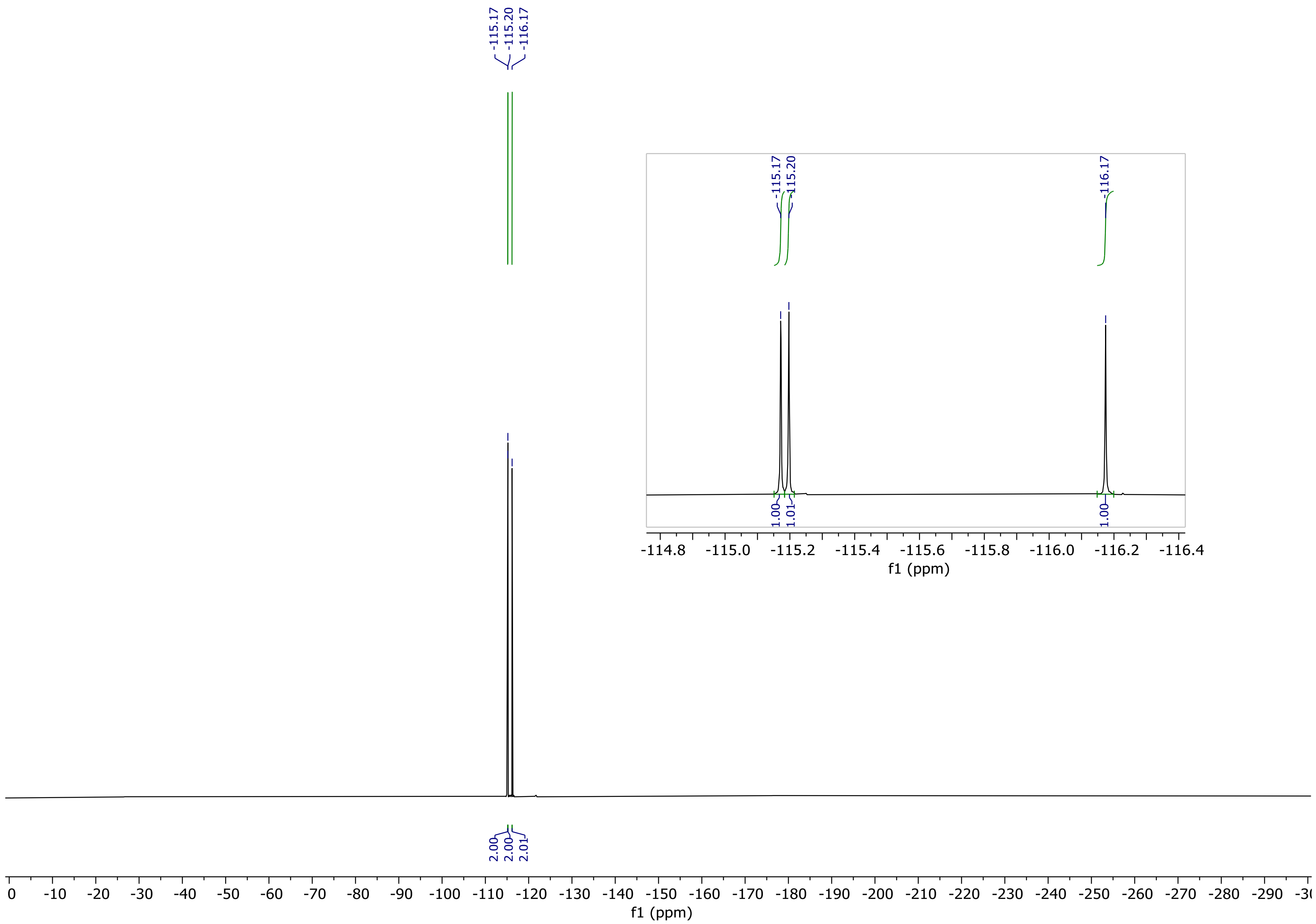

$^{19}F\{^1H\}$ NMR of compound **1j** (376 MHz, CD_2Cl_2, 25 °C).

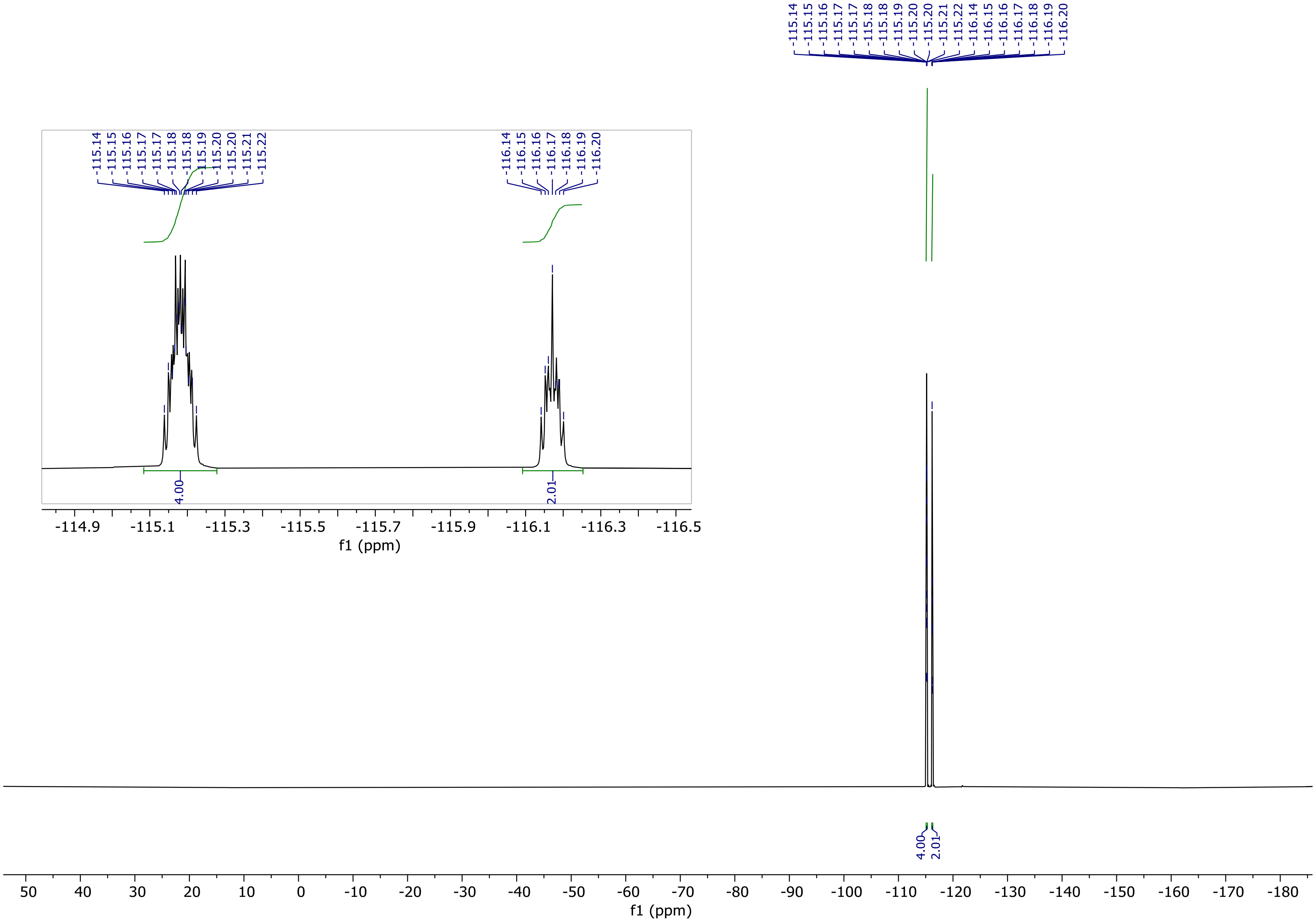

^{19}F NMR of compound **1j** (471 MHz, CD_2Cl_2, 25 °C).

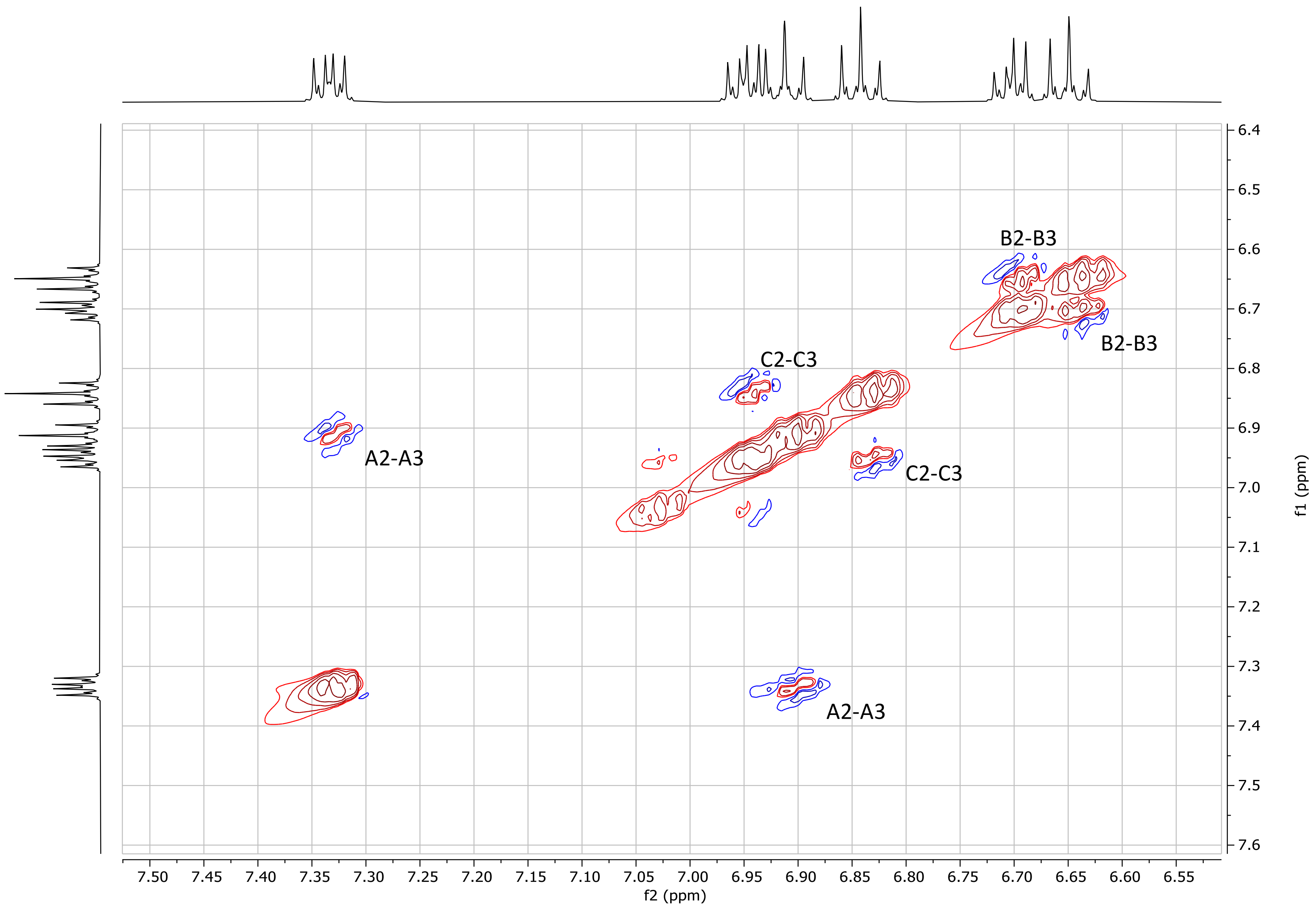

NOESY NMR of compound **1j** (500 MHz, CD_2Cl_2, 25 °C).

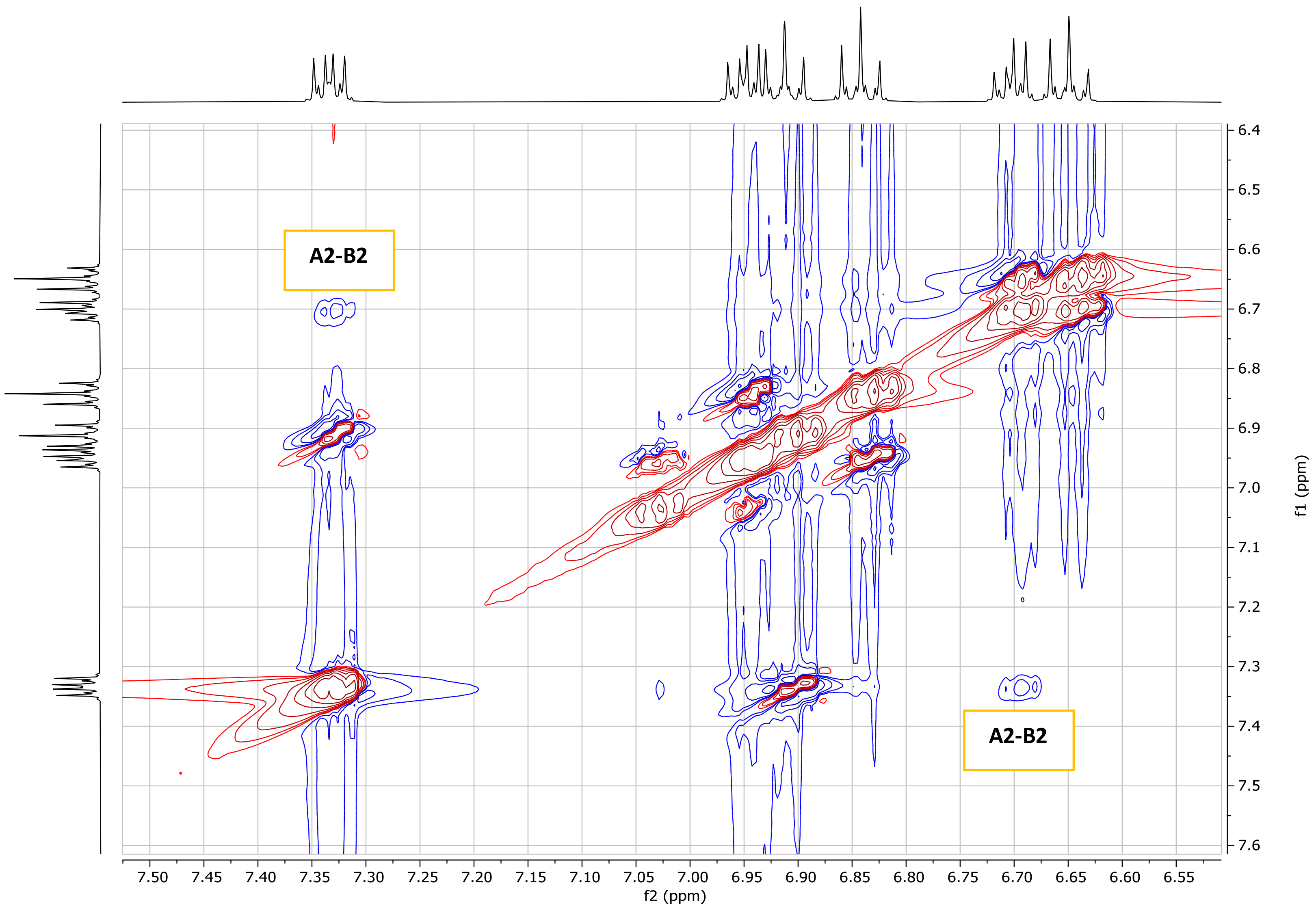

NOESY NMR (increased intensity) of compound **1j** (500 MHz, CD_2Cl_2, 25 °C).

1,2,3,4,5,5-Hexa(4'-bromophenyl)cyclopenta-1,3-diene (1k):

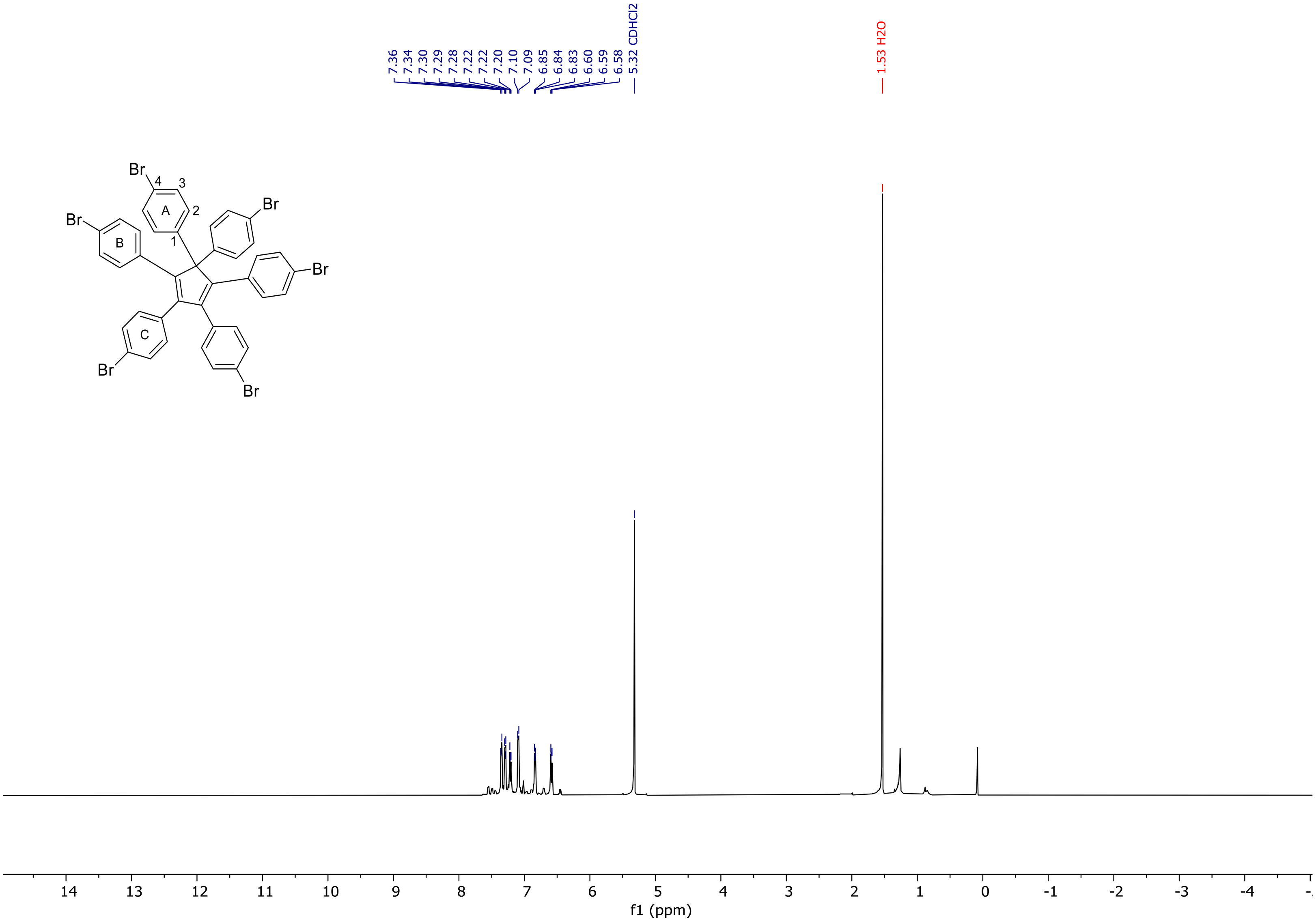

^{1}H-NMR of compound **1k** (500 MHz, CD_2Cl_2, 25 °C, signals corresponding to partially dehalogenated products are present).

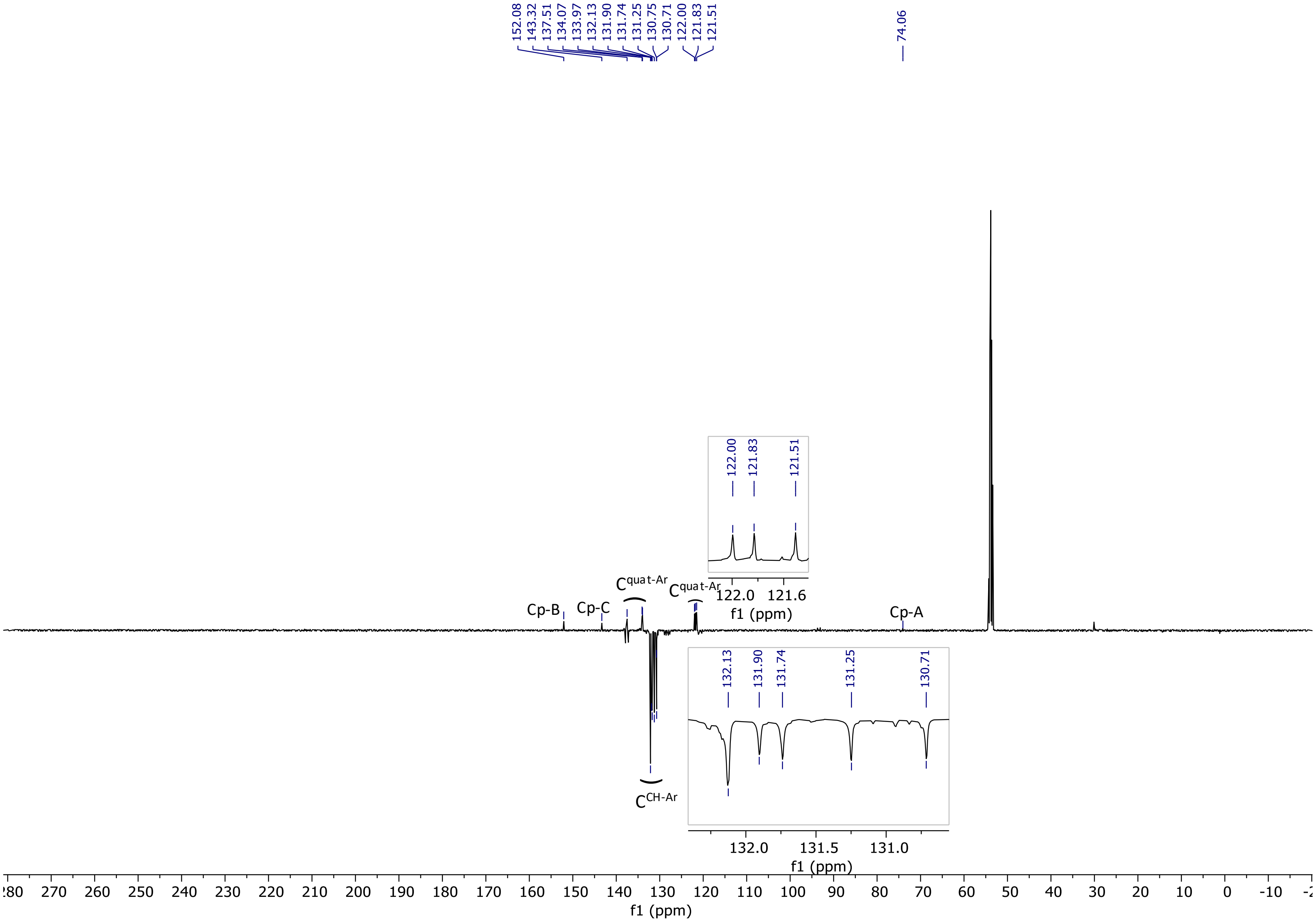

^{13}C-Jmod-NMR of compound **1k** (126 MHz, CD_2Cl_2, 25 °C, signals corresponding to partially dehalogenated products are present).

Spiro[*1H*-cyclopenta[*l*]phenanthrene-1,9'-*[9H]*fluorene] (4):

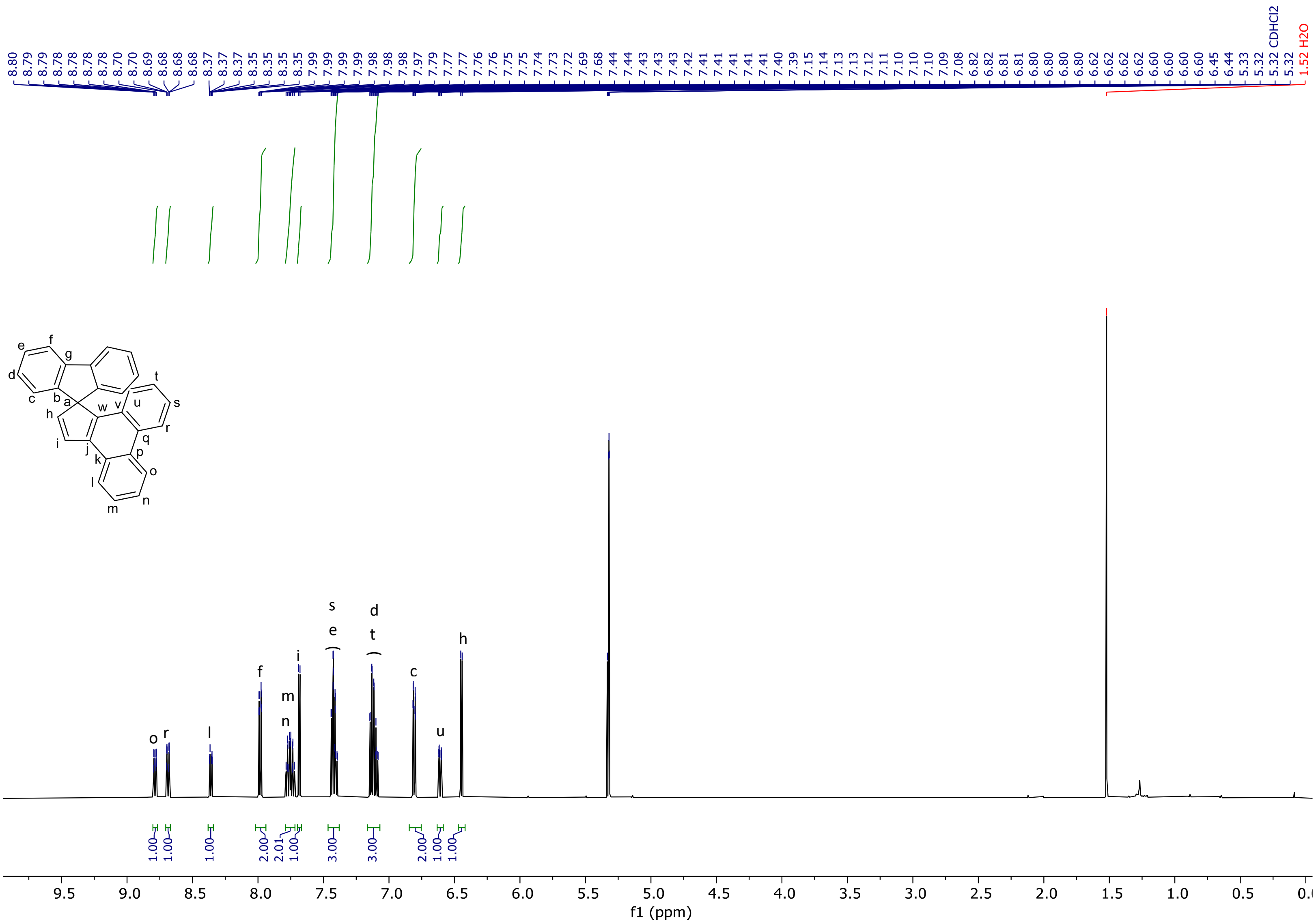

^{1}H-NMR of compound **4** (500 MHz, CD_2Cl_2, 25 °C).

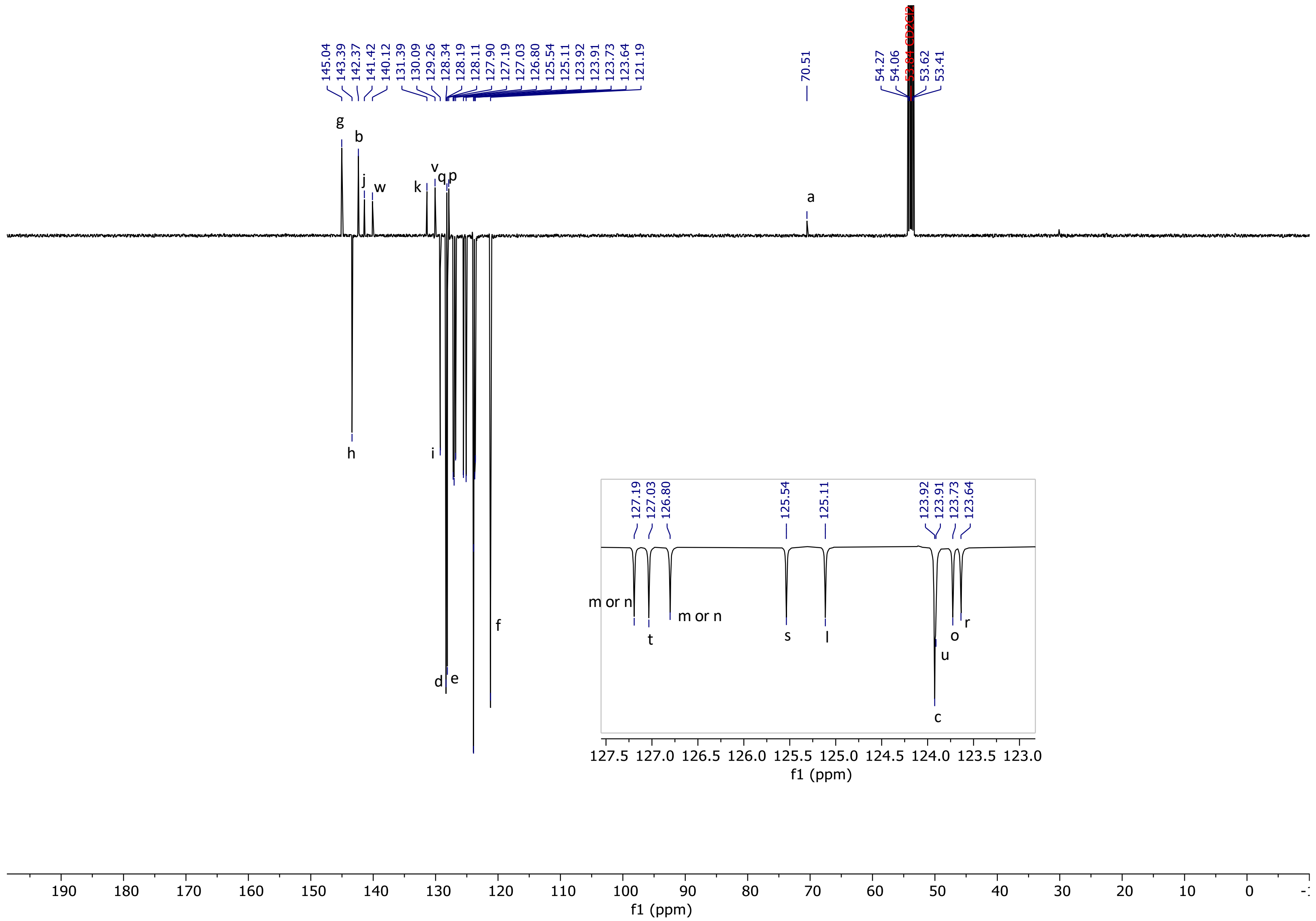

^{13}C-Jmod-NMR of compound **4** (126 MHz, CD_2Cl_2, 25 °C).

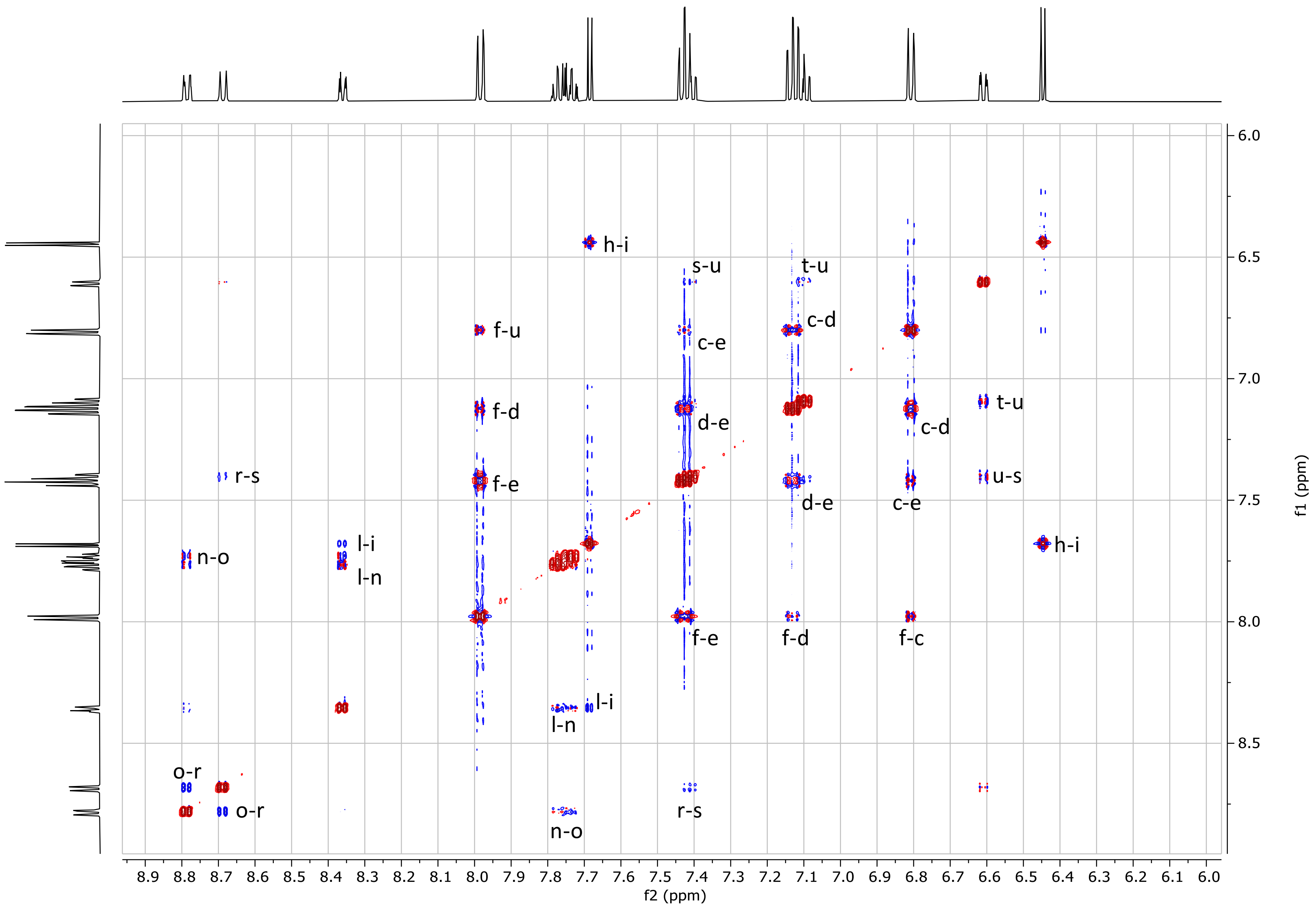

NOESY NMR of compound **4** (500 MHz, CD_2Cl_2, 25 °C).

1,1,2,3-Tetraphenyl-*1H*-cyclopenta[*l*]phenanthrene (5):

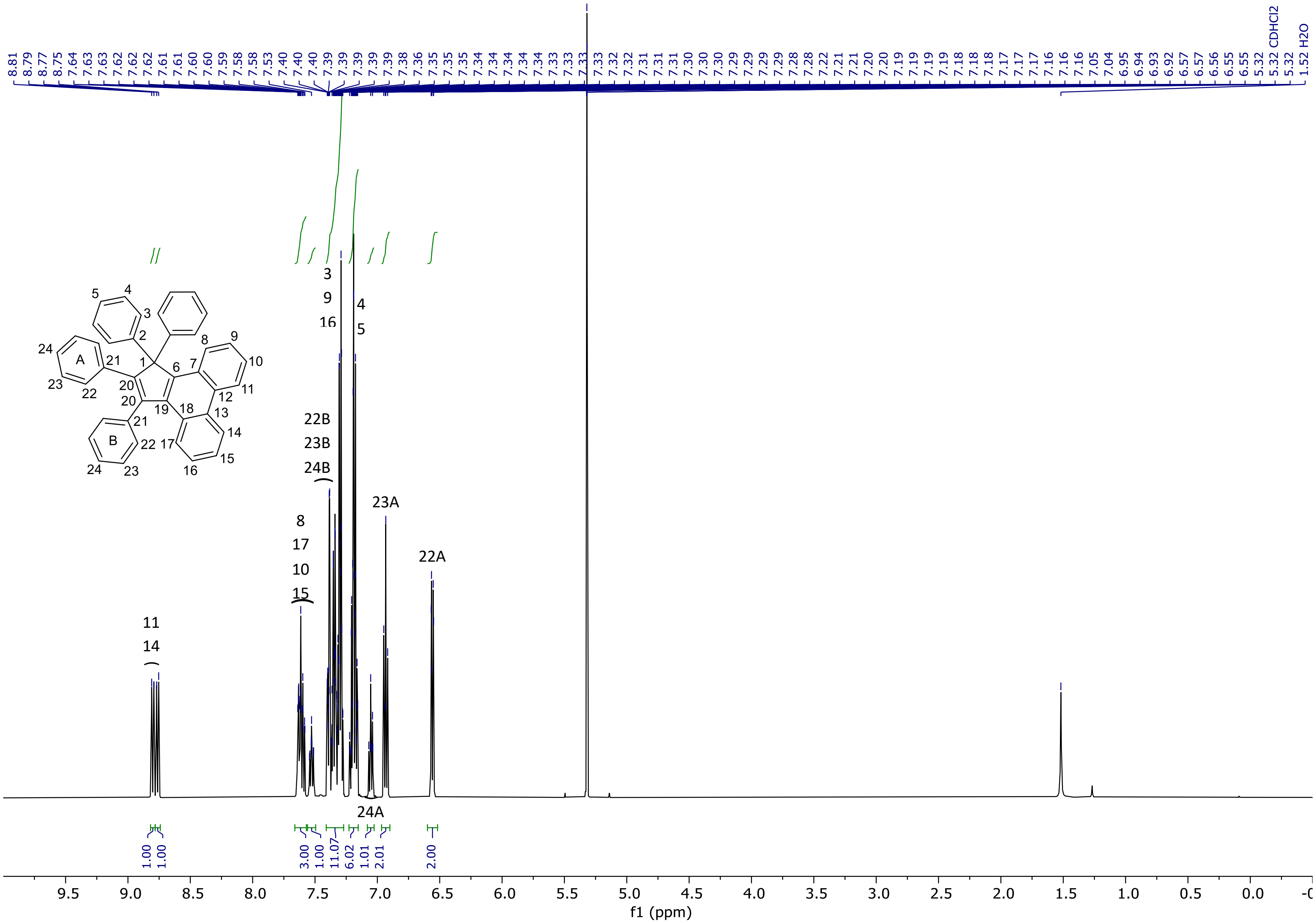

^{1}H-NMR of compound **5** (500 MHz, CD_2Cl_2, 25 °C).

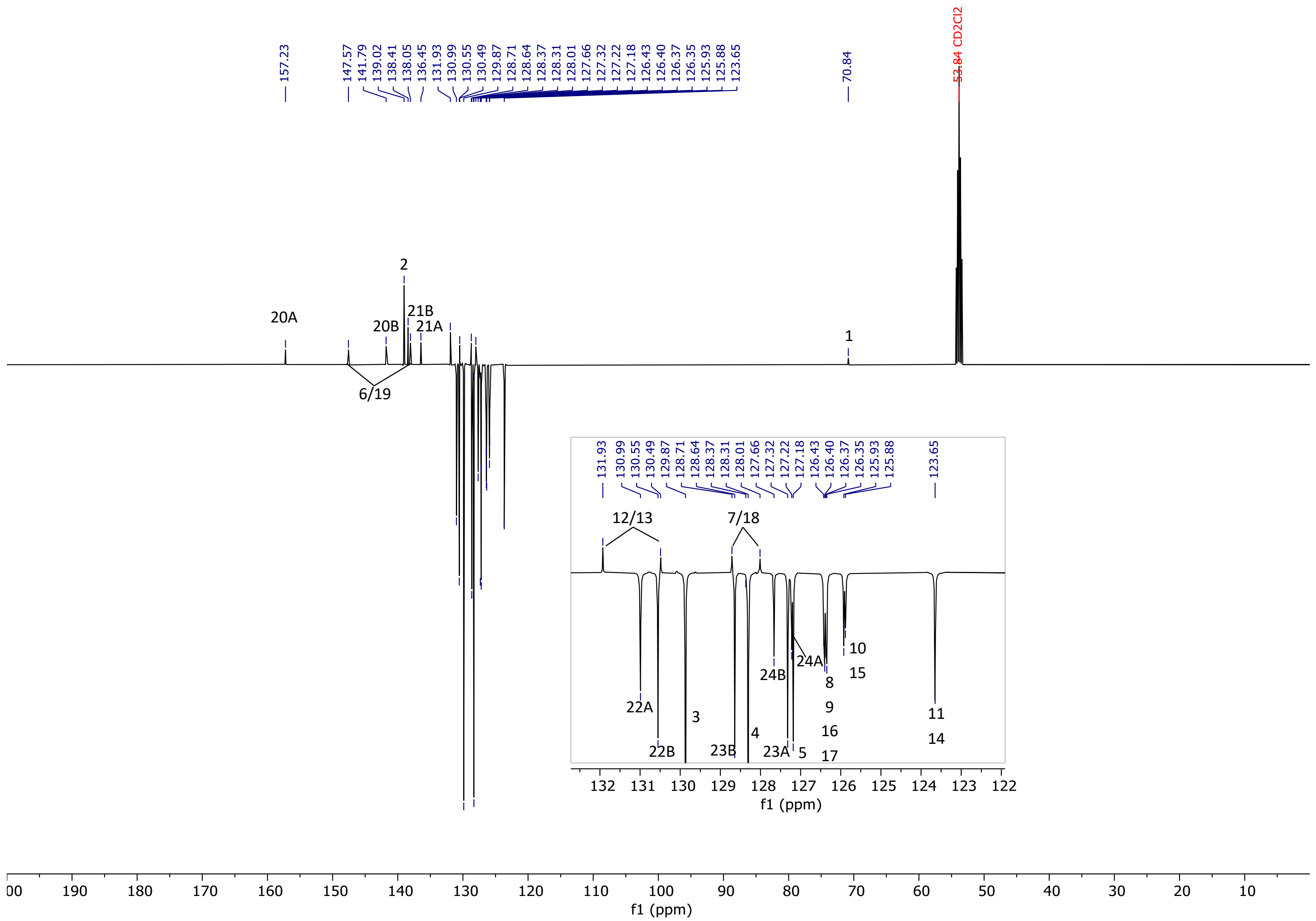

^{13}C-Jmod-NMR of compound **5** (126 MHz, CD_2Cl_2, 25 °C).

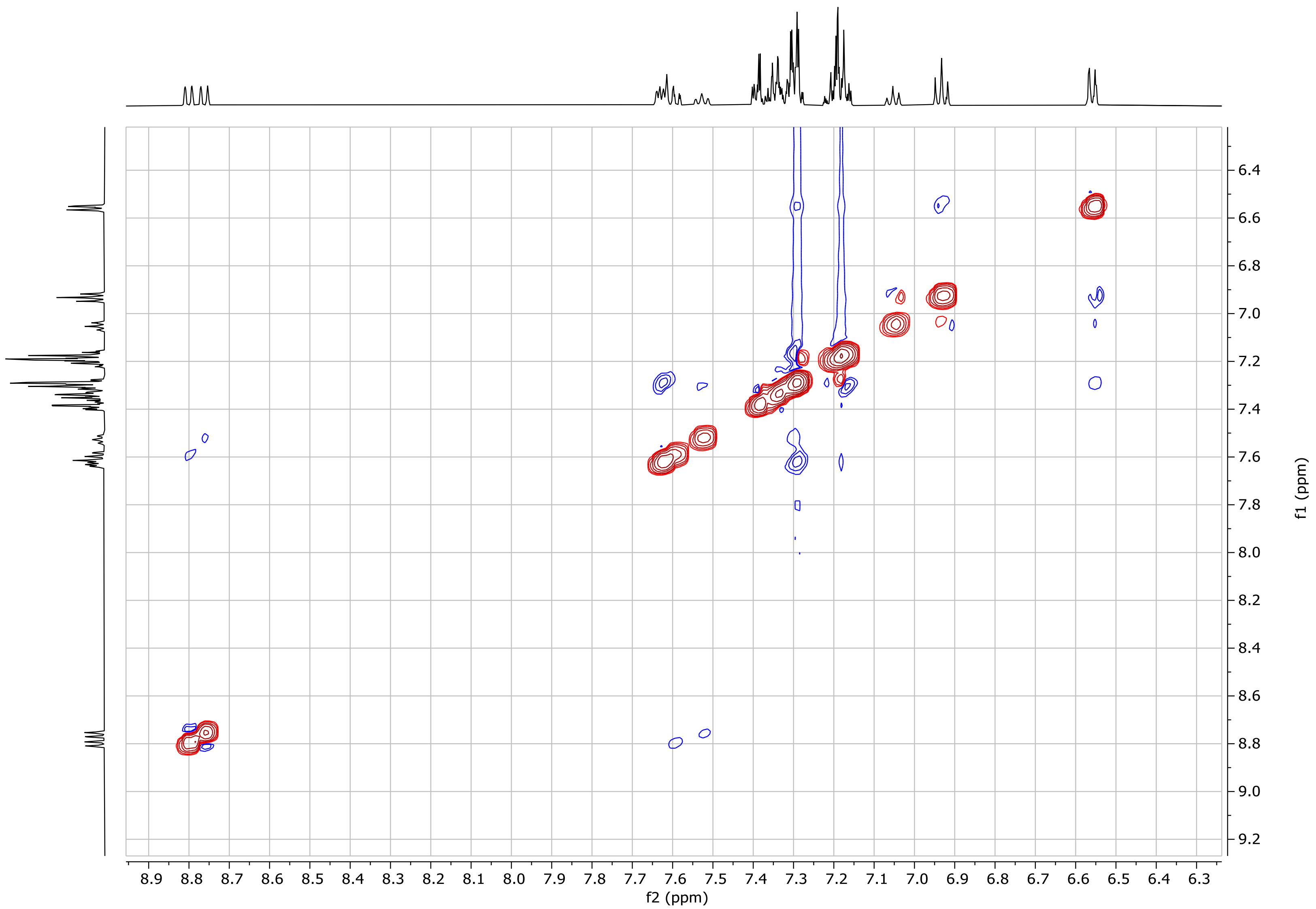

NOESY NMR of compound **5** (500 MHz, CD_2Cl_2, 25 °C).

17,17-Diphenylcyclopenta[*l,l'*]diphenanthrene (6a):

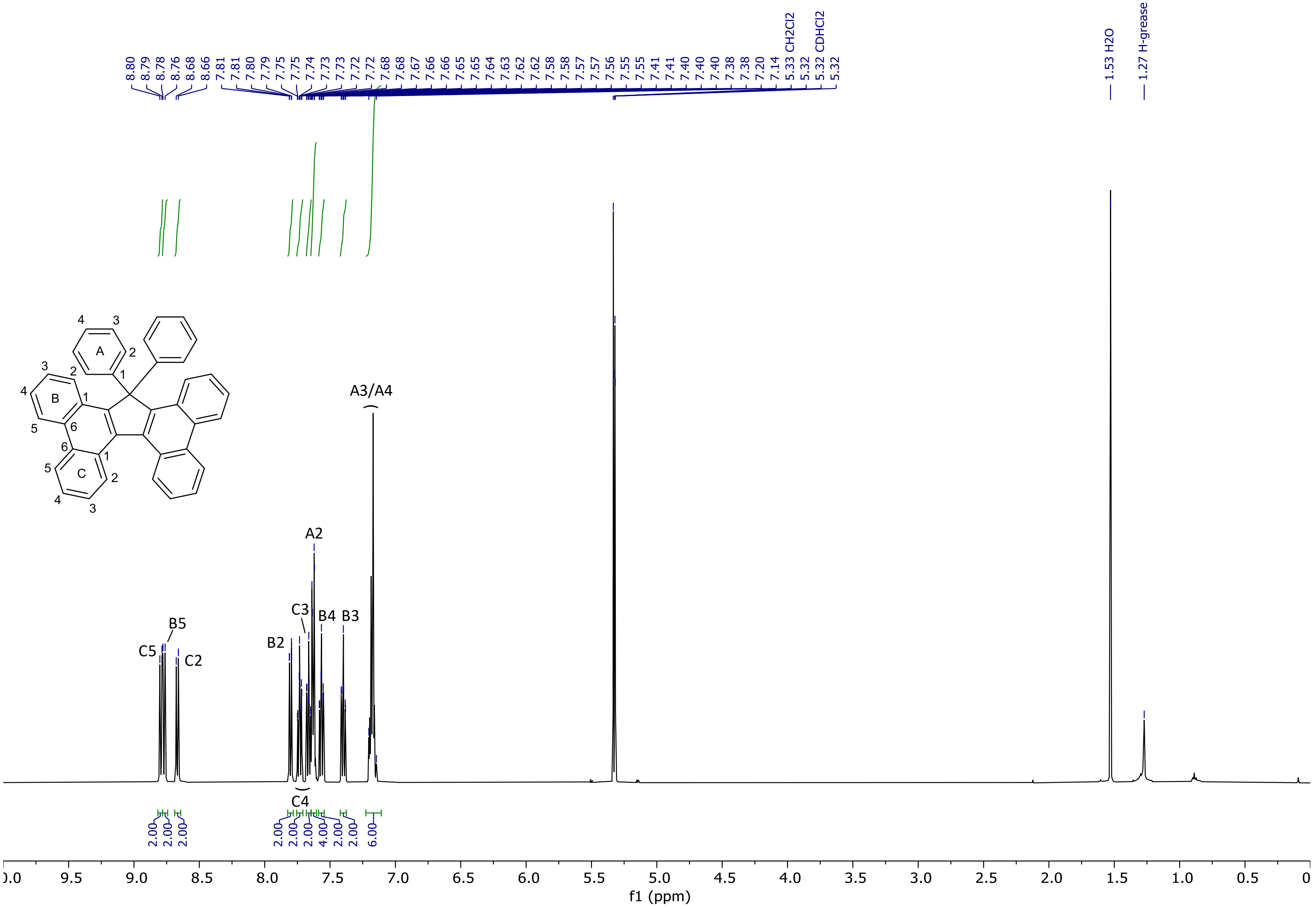

^{1}H-NMR of compound **6a** (500 MHz, CD_2Cl_2, 25 °C).

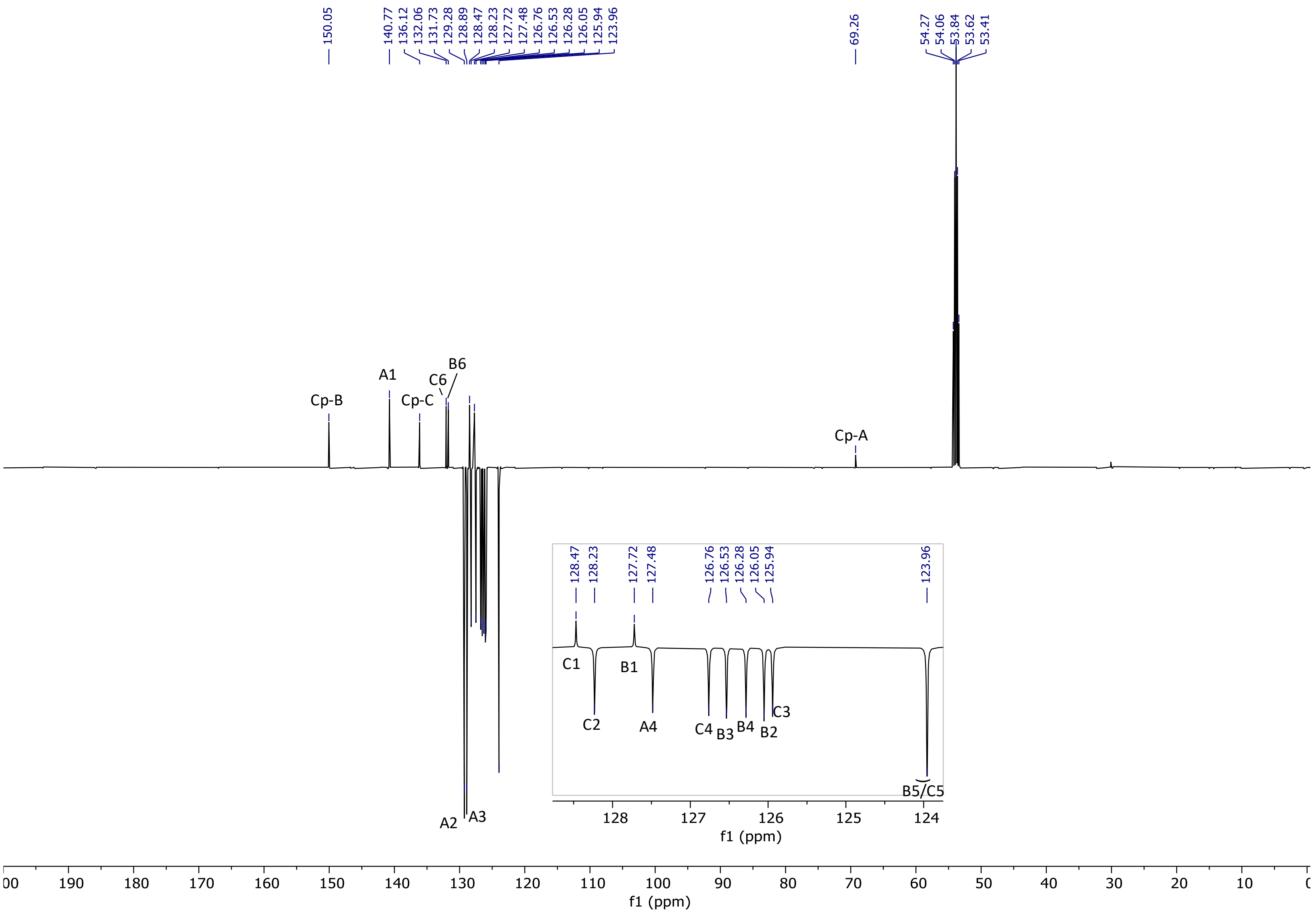

^{13}C-Jmod-NMR of compound **6a** (126 MHz, CD_2Cl_2, 25 °C).

3,6,11,14-Tetra(*tert*-butyl)-17,17-di(4'-*tert*-butylphenyl)cyclopenta[*l*,*l'*]diphenanthrene (6b):

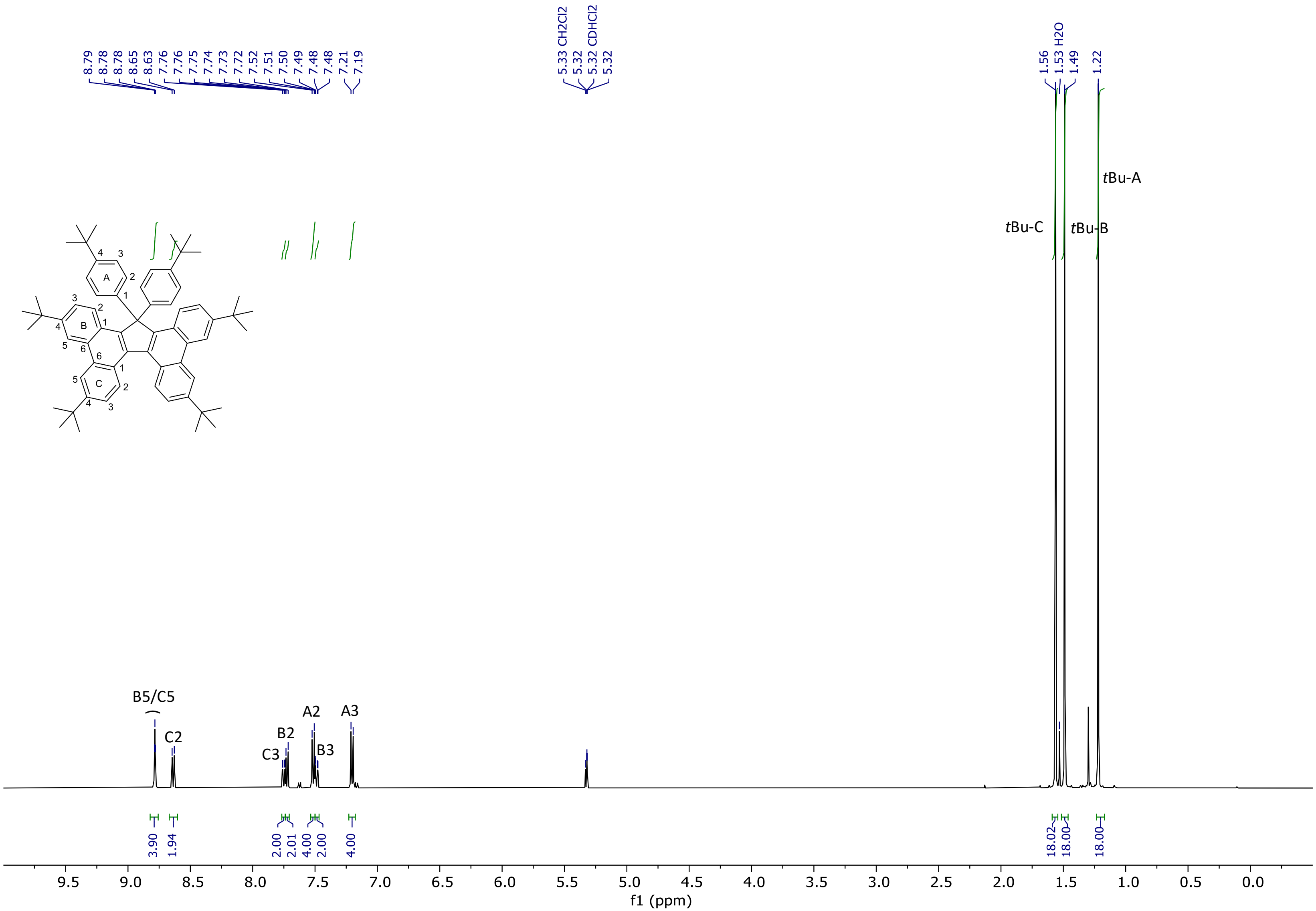

^{1}H-NMR of compound **6b** (500 MHz, CD_2Cl_2, 25 °C).

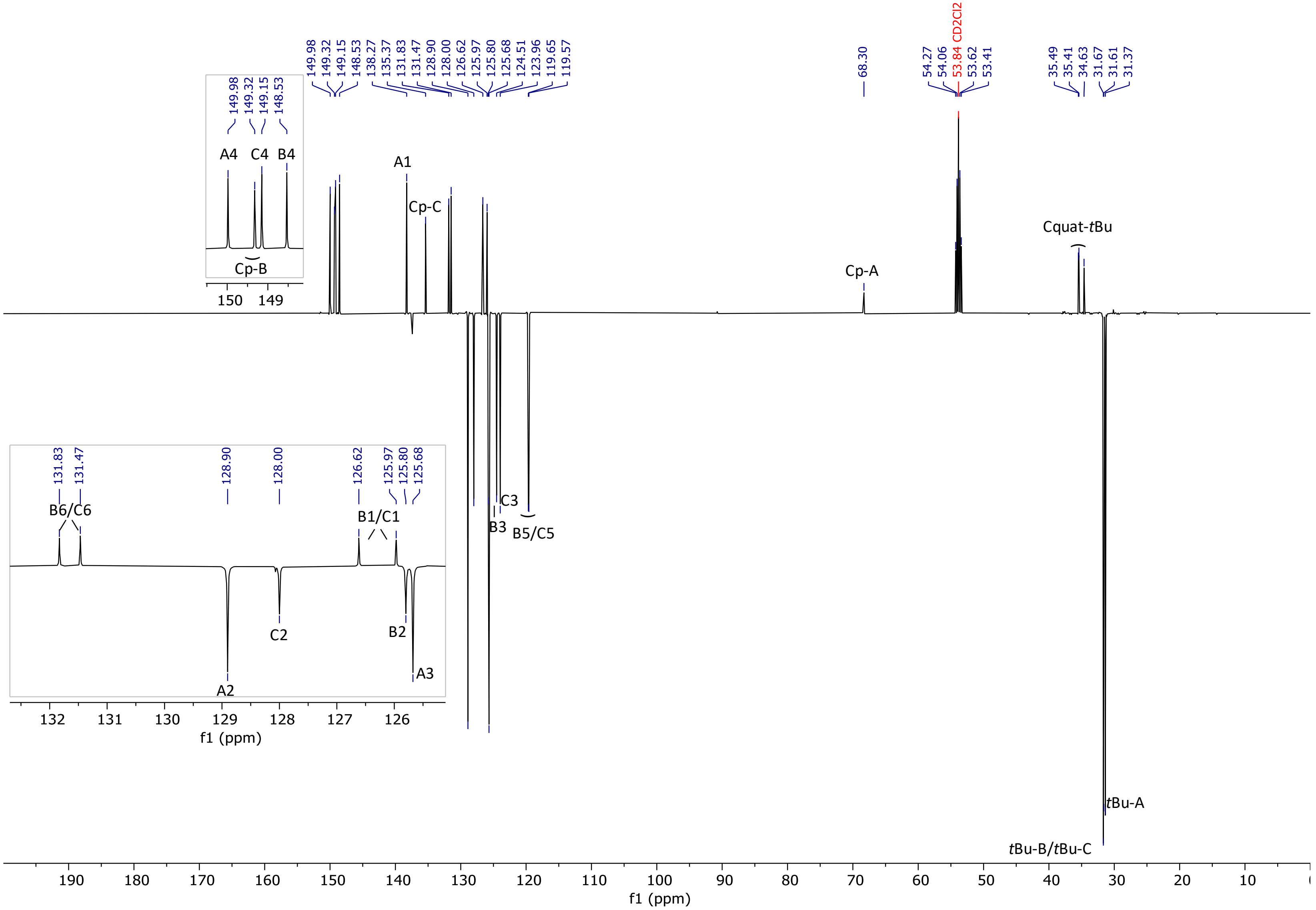

^{13}C-Jmod-NMR of compound **6b** (126 MHz, CD_2Cl_2, 25 °C).

3,6,11,14-Tetramethyl-17,17-di(4’-methylphenyl)cyclopenta[*l,l’*]diphenanthrene (6c):

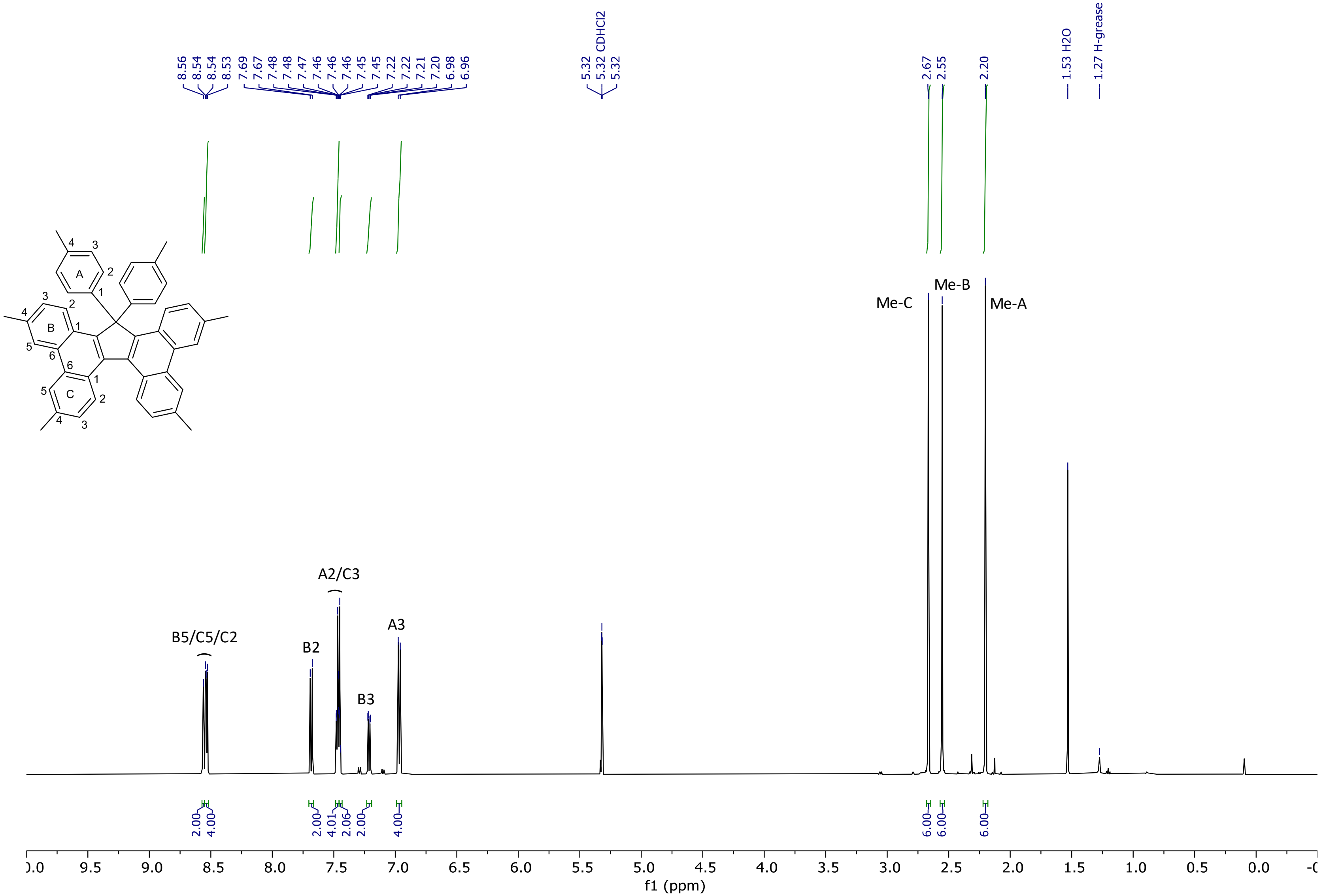

^{1}H-NMR of compound **6c** (500 MHz, CD_2Cl_2, 25 °C).

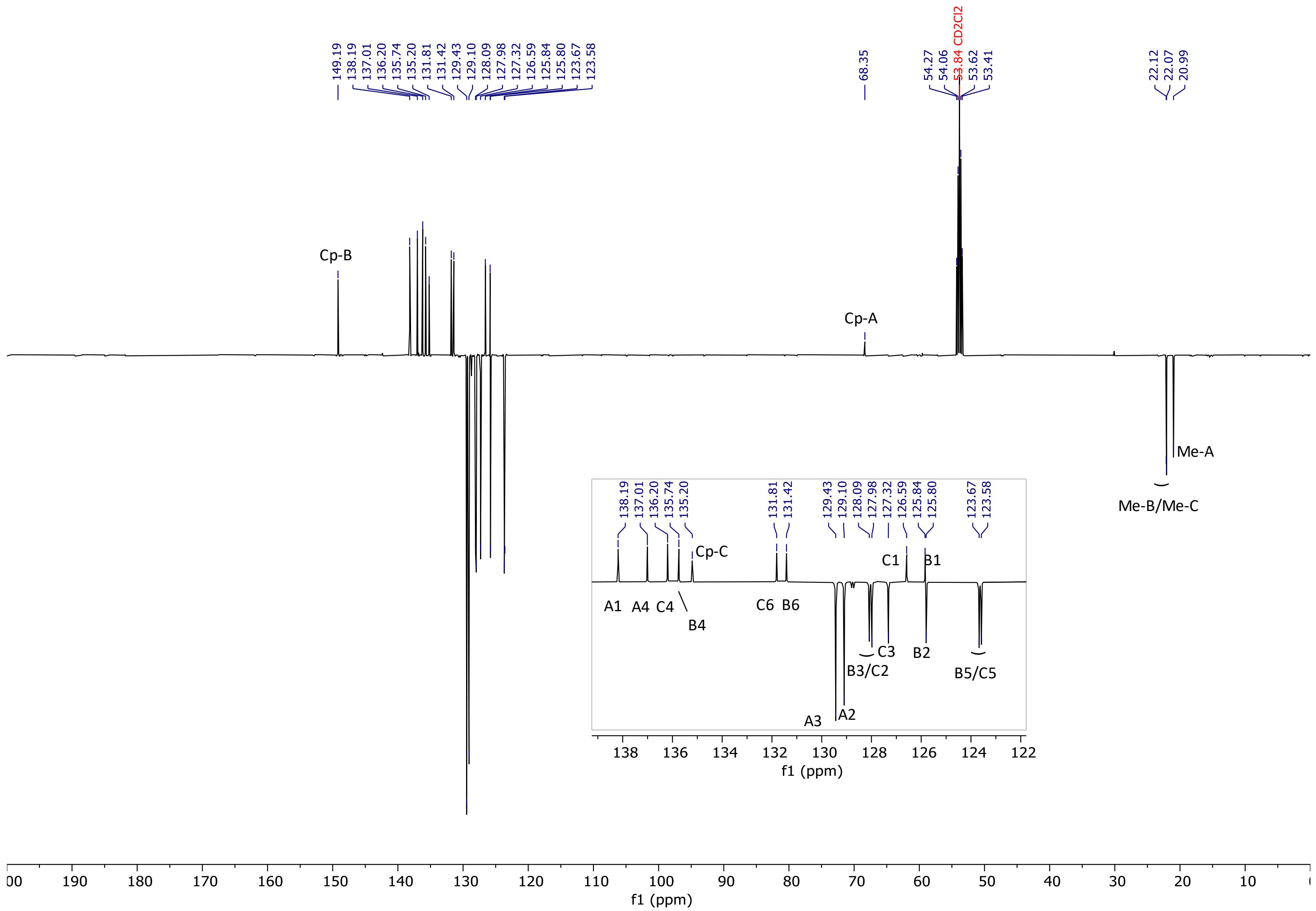

^{13}C-Jmod-NMR of compound **6c** (126 MHz, CD_2Cl_2, 25 °C).

5-(4'-Fluorophenyl)-1,2,3,4,5-pentaphenylcyclopenta-1,3-diene (7):

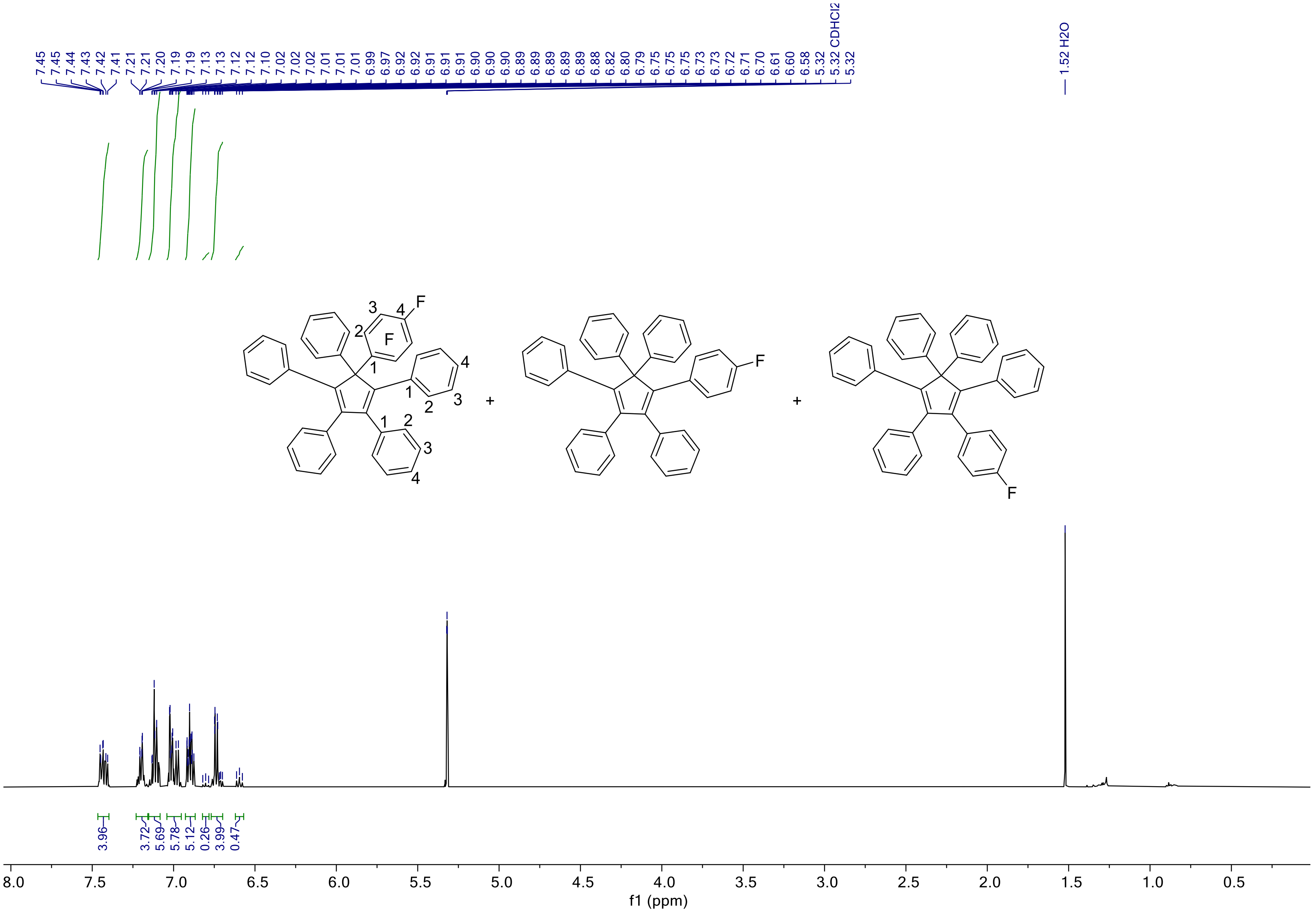

^{1}H-NMR of compound **7** (500 MHz, CD_2Cl_2, 25 °C), obtained as a 73:18:9 mixture of regioisomers.

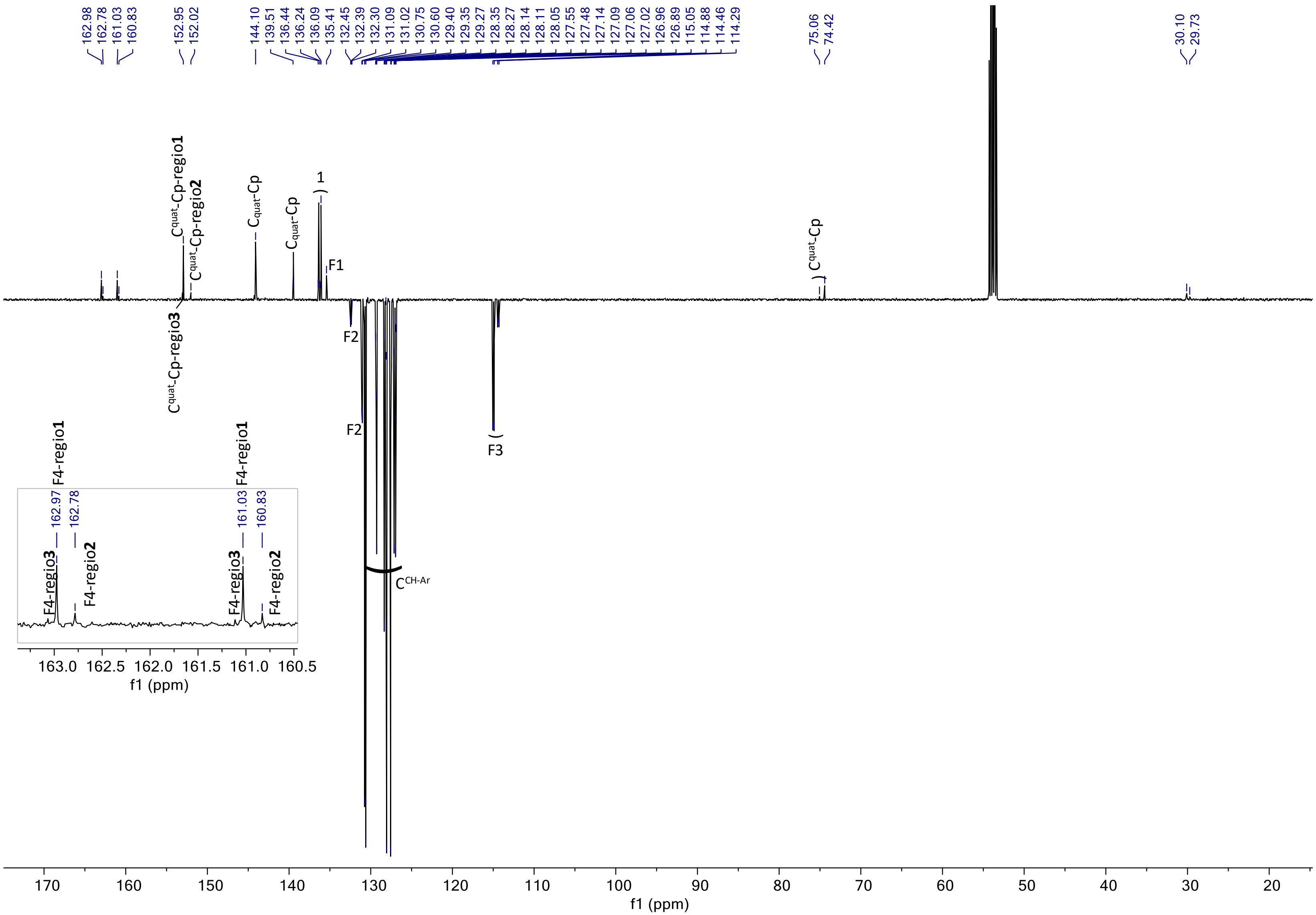

^{13}C-Jmod-NMR of compound **7** (126 MHz, CD_2Cl_2, 25 °C), obtained as a 73:18:9 mixture of regioisomers.

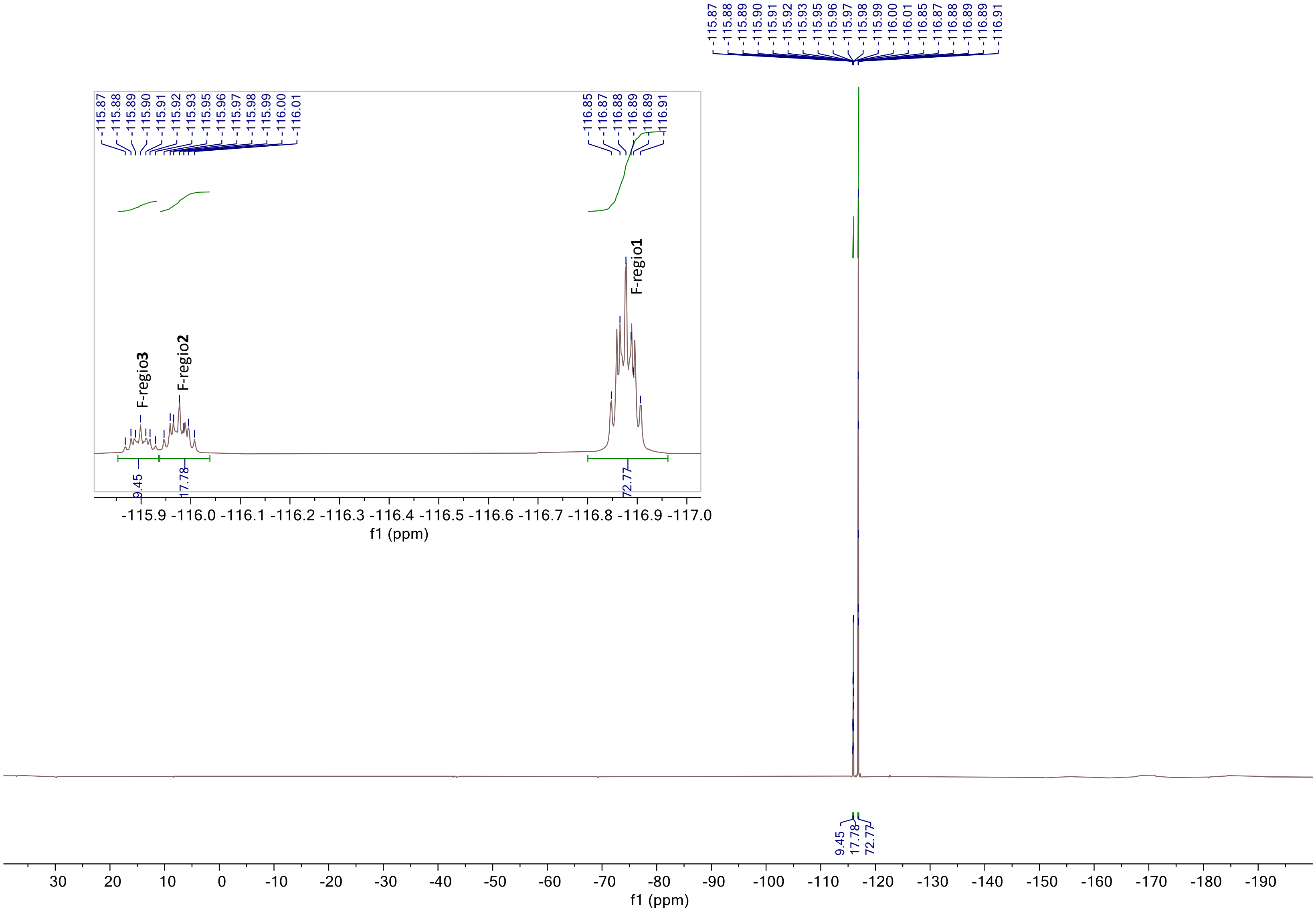

^{19}F NMR of compound **7** (471 MHz, CD_2Cl_2, 25 °C), obtained as a 73:18:9 mixture of regioisomers.

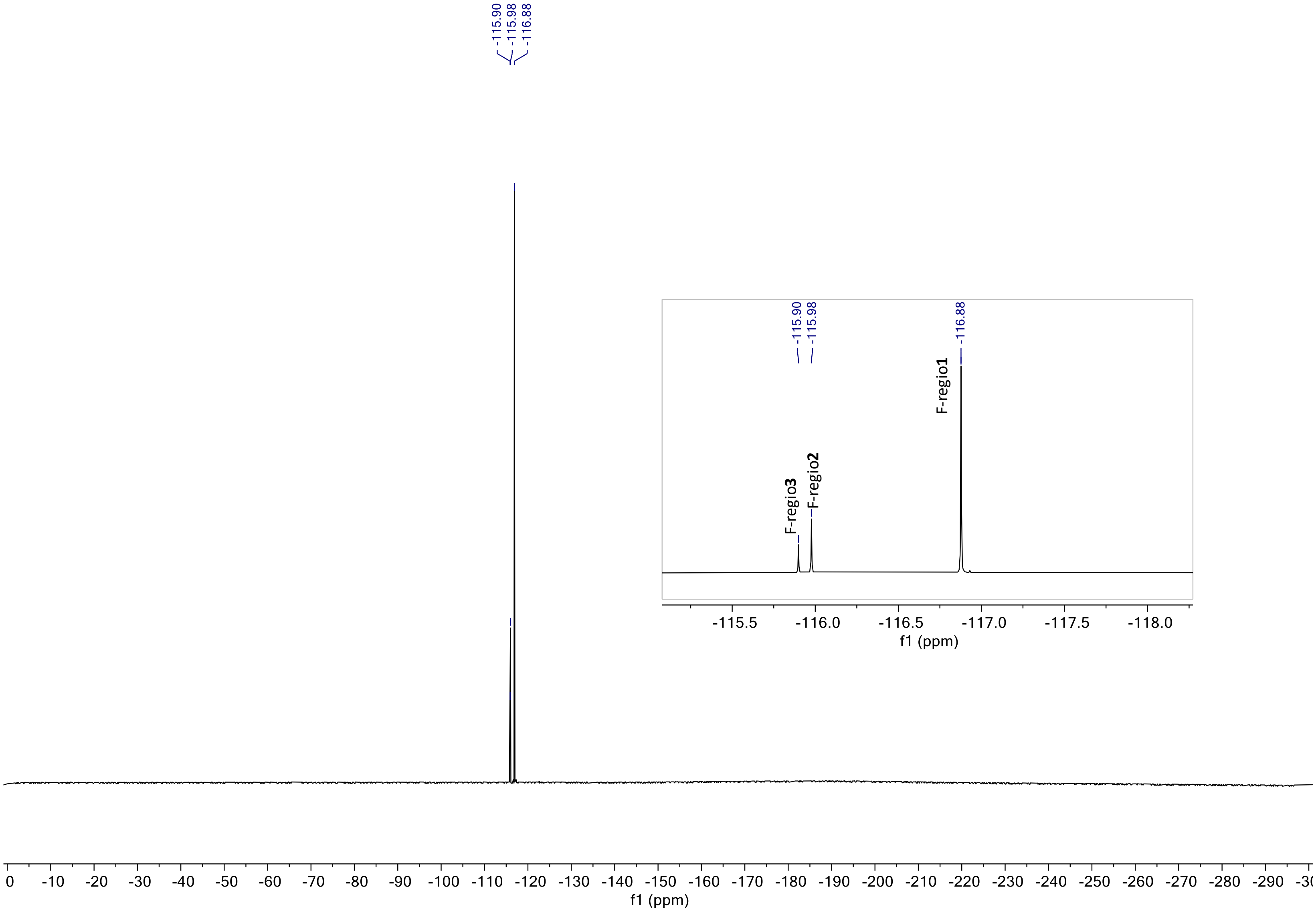

$^{19}F\{^{1}H\}$ NMR of compound **7** (376 MHz, CD_2Cl_2, 25 °C), obtained as a 73:18:9 mixture of regioisomers.

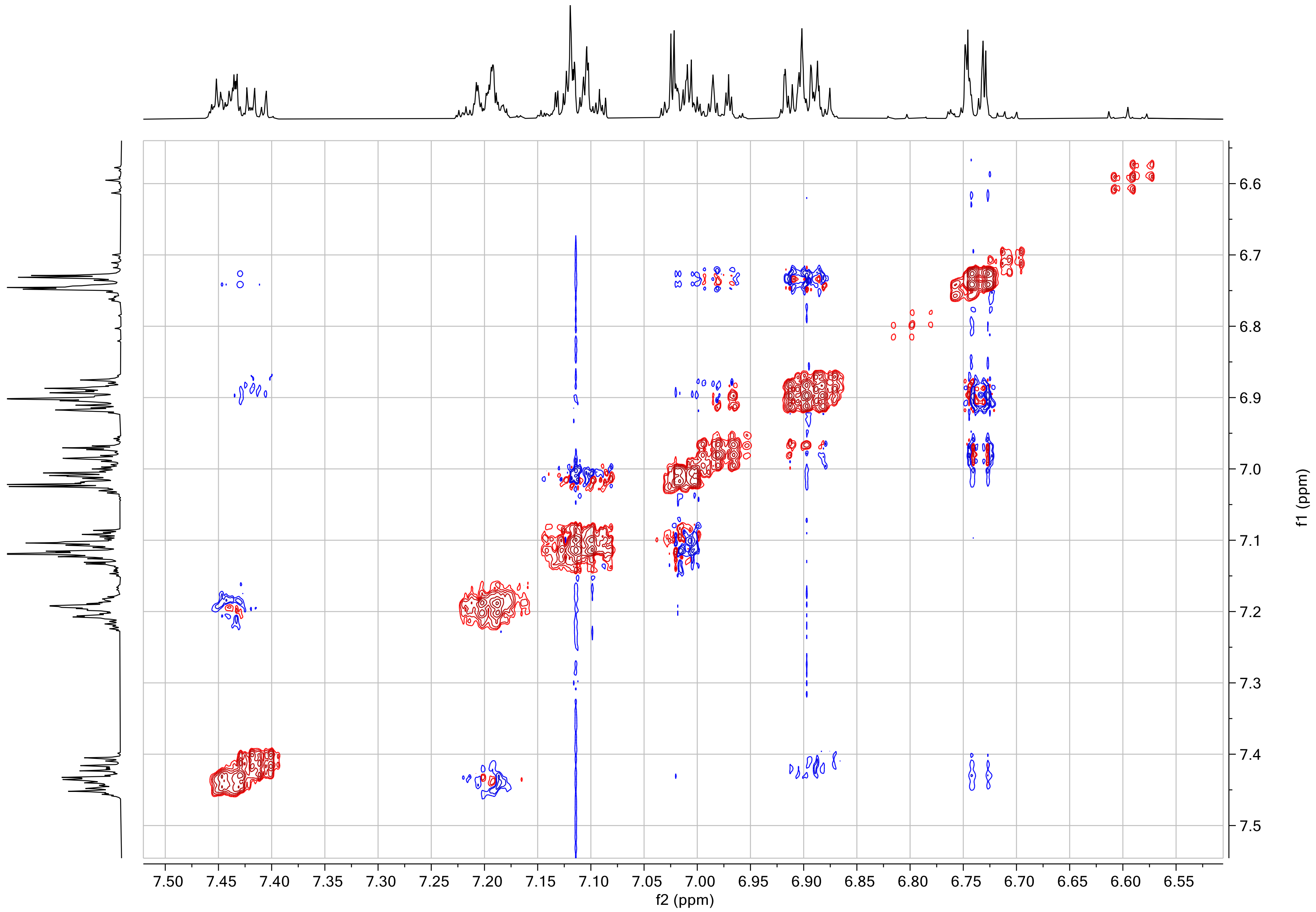

NOESY NMR of compound **7** (500 MHz, CD_2Cl_2, 25 °C), obtained as a 73:18:9 mixture of regioisomers.

V. Absorption and emission properties

V.1. Absorption spectra of compounds 1a and 1h

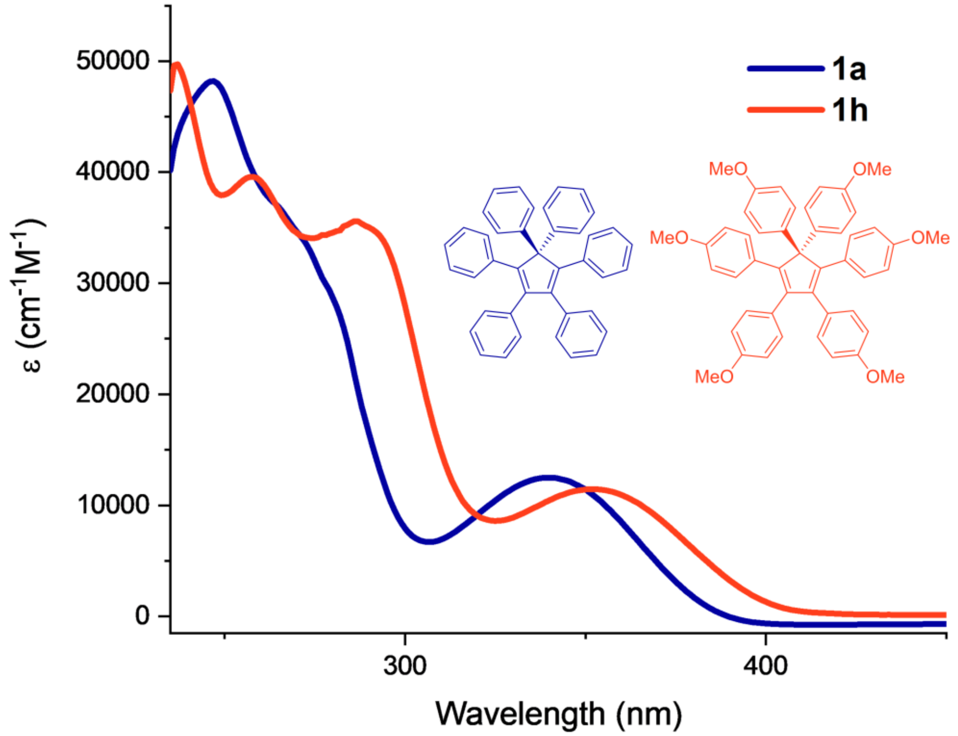

Figure S2. Absorption spectra of **1a** and **1h**, 10^{-5} M in THF at 298 K.

V.2. Aggregation-induced emission of compounds 1a and 1h

a. Photoluminescence in different THF/H_2O ratios

To record the photoluminescence under varying THF/H_2O ratios (0% to 100%), a stock solution of **1a** or **1h** was prepared in THF (10^{-4} M) and 5 µL of this stock solution were diluted in 3 mL of a previously prepared mixture of THF/H_2O and transferred to a quartz cuvette.

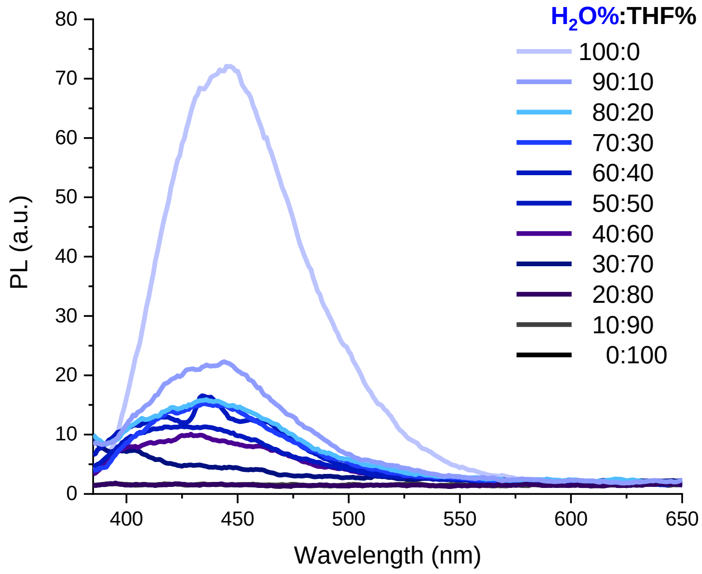

Figure S3. Photoluminescence spectra of **1a**, 10^{-7} M, λ_{Ex} = 350 nm, in different ratios of H_2O and THF. The AIE phenomenon is visible, with the λ_{max} located at 443 nm in pure H_2O.

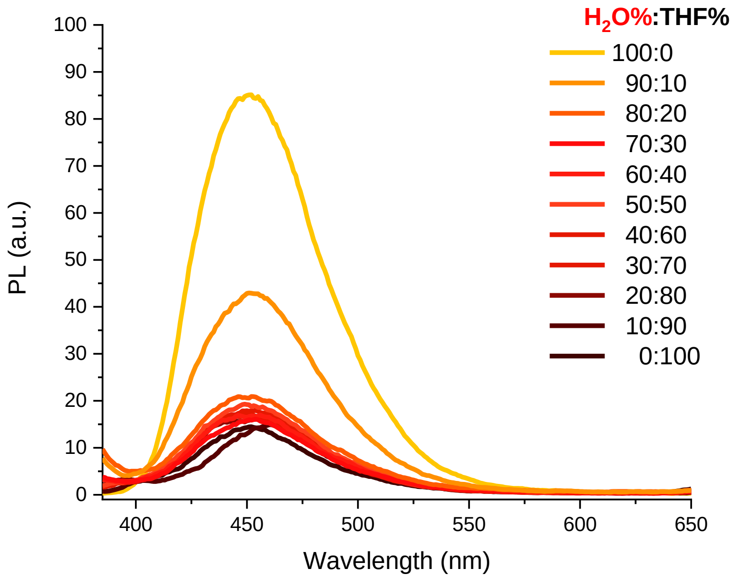

Figure S4. Photoluminescence spectra of **1h**, 10^{-7} M, λ_{Ex} = 350 nm, in different ratios of H_2O and THF. The AIE phenomenon is visible, with the λ_{max} located at 453 nm in pure H_2O.

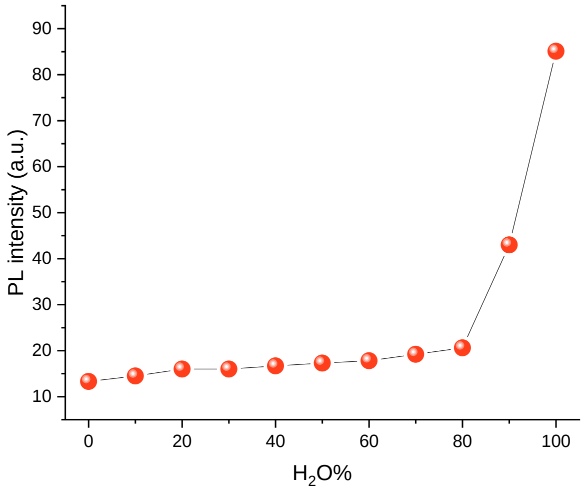

Figure S5. Data plot representation of photoluminescence intensity of **1h** at λ_{max} = 453 nm (λ_{Ex} = 350 nm) vs H_2O fraction in the THF/H_2O mixture.

b. Titration experiments

To carry out the titration experiments, a stock solution of **1a** or **1h** was prepared in THF (10^{-4} M) and 5 µL of the stock solution were diluted in 3 mL of deionised water to form a homogeneous dispersion. The latter was transferred to a quartz cuvette and the emission spectrum was recorded exciting at 350 nm. Thereafter, 5 aliquots of THF (20 µL) were subsequently added to the sample measuring again the emission spectra after each aliquot.

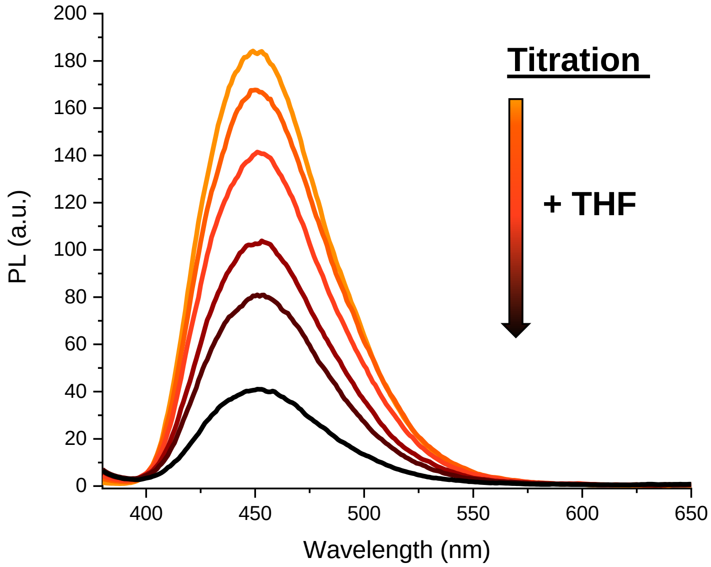

Figure S6. Photoluminescence spectrum of **1h** in pure H_2O (light orange curve), 10^{-7} M, λ_{Ex} = 350 nm, and photoluminescence spectra resulting from the successive additions of small aliquots (20 µL) of THF (darker curves).

V.3. Absorption and emission spectra of compounds 6a-c

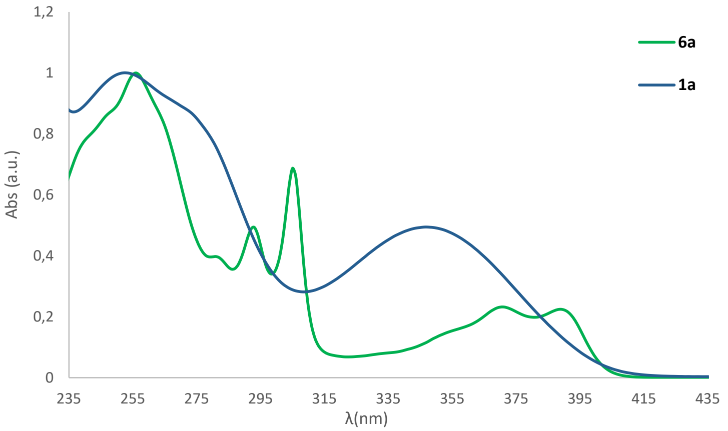

Figure S7. Normalised UV-Vis absorption spectra of compounds **1a** and **6a** in CH_2Cl_2 solutions at 298 K.

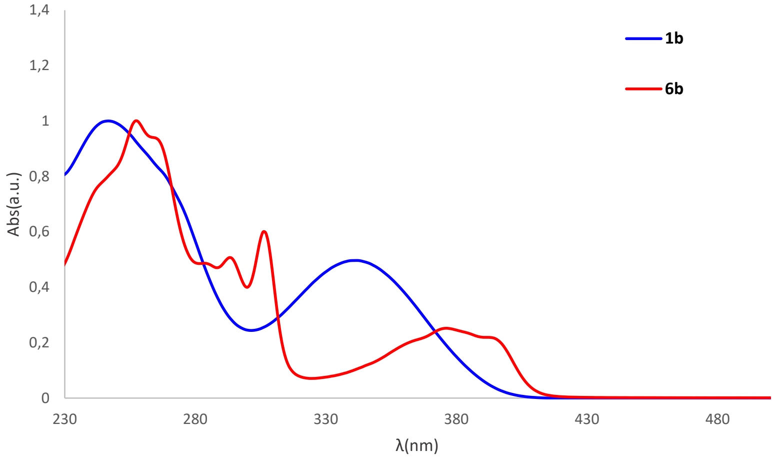

Figure S8. Normalised UV-Vis absorption spectra of compounds **1b** and **6b** in CH_2Cl_2 solutions at 298 K.

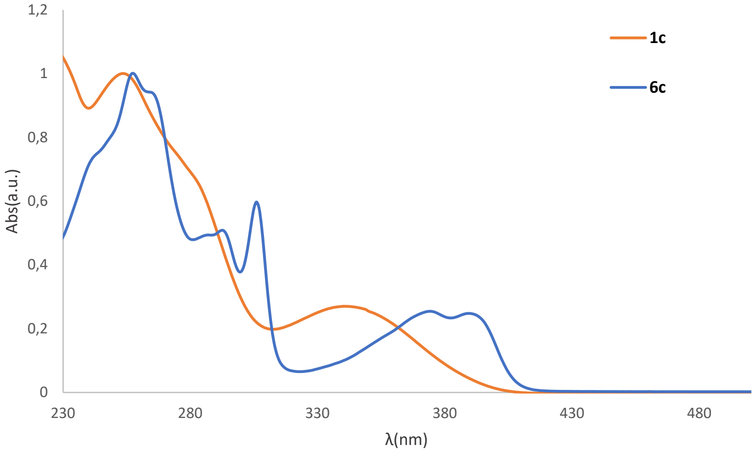

Figure S9. Normalised UV-Vis absorption spectra of compounds **1c** and **6c** in CH_2Cl_2 solutions at 298 K.

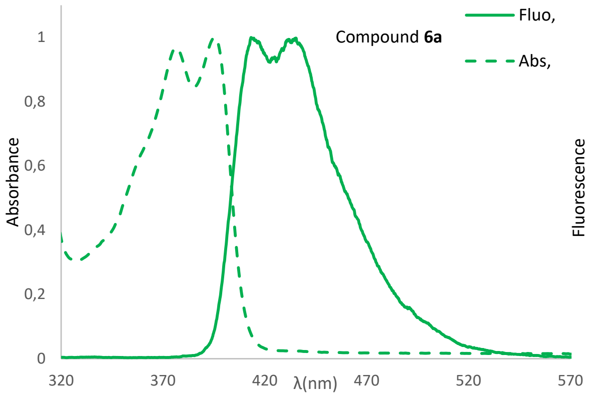

Figure S10. Normalised absorption (dashed line) and emission (solid line) spectra of compounds **6a** in CH_2Cl_2 solution at 298 K. Excitation wavelength is 300 nm.

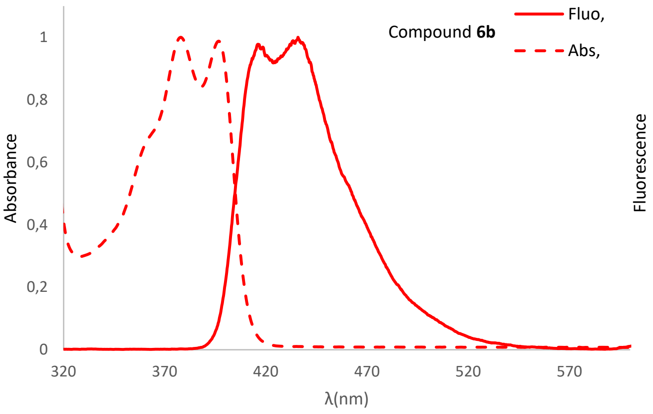

Figure S11. Normalised absorption (dashed line) and emission (solid line) spectra of compound **6b** in CH_2Cl_2 solution at 298 K. Excitation wavelength is 300 nm.

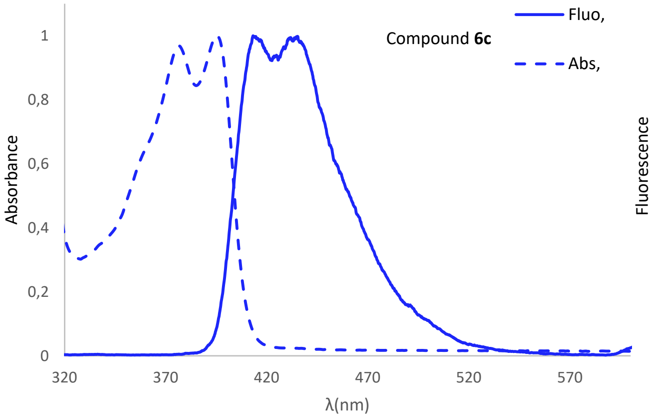

Figure S12. Normalised absorption (dashed line) and emission (solid line) spectra of compound **6c** in CH_2Cl_2 solution at 298 K. Excitation wavelength is 300 nm.

VI. Cyclic voltammetry

The redox properties of pentaphenylcyclopentadiene (**2a**) and hexaphenylcyclopentadiene (**1a**) were examined by means of cyclic voltammetry performed in anhydrous dichloromethane (Figure S13). In the oxidation region of the voltammograms, only one reversible wave is observed for each compound. These waves are attributed to the one-electron oxidation of the polyarylcyclopentadienes scaffolds, with the oxidation of pentaphenylcyclopentadiene **2a** ($E_{1/2}$ = 1.13 V *vs*. SCE) occurring at a lower potential than that of hexaphenylcyclopentadiene **1a** ($E_{1/2}$ = 1.26 V *vs*. SCE).

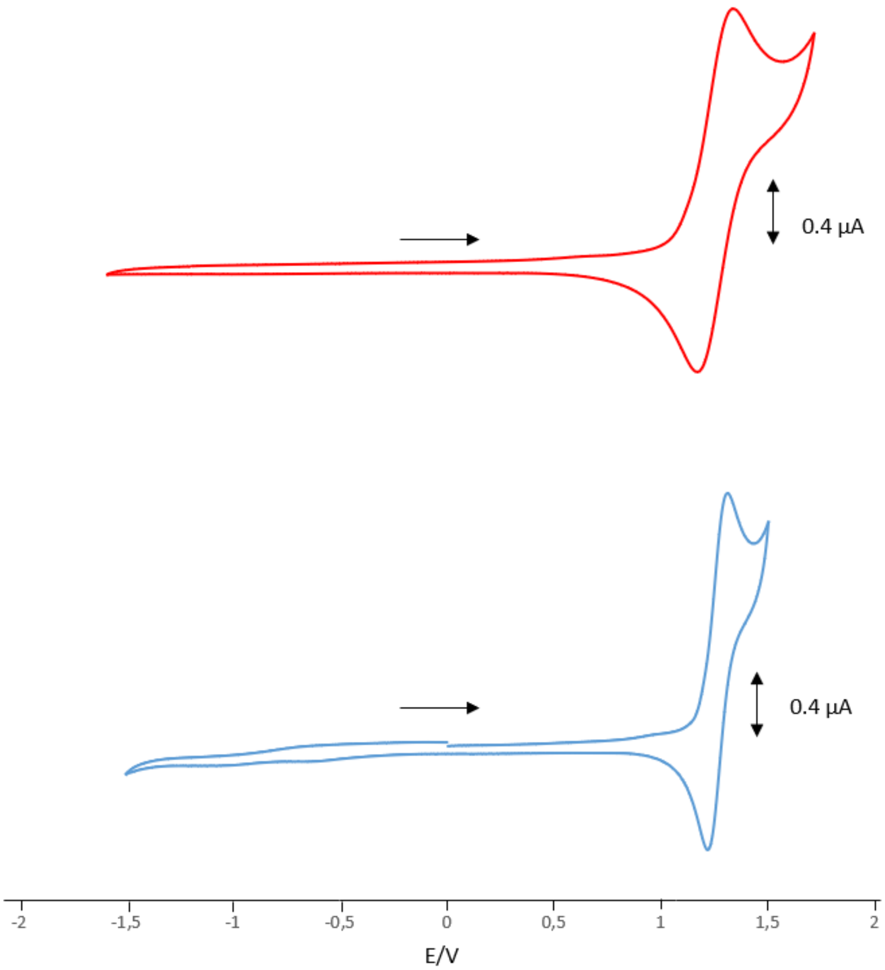

Figure S13. Cyclic voltammograms of hexaphenylcyclopentadiene **1a** (red) and pentaphenylcyclopentadiene **2a** (blue) in CH_2Cl_2 (0.1 M *n*Bu_4NPF_6, 25 °C) *vs*. SCE at a scan rate of 0.1 V s^{-1}. Concentrations: [**1a**] = 1.10^{-4} M, [**2a**]= 2.10^{-4} M.

VII. Crystallographic data

Crystallographic data were collected on a Bruker-AXS Kappa APEX II Quazar diffractometer (**1a** and **7**) equipped with a 30W air-cooled microfocus source using Mo K_{α} radiation (λ=0.71073 Å) or on a Bruker-AXS D8-Venture diffractometer (**1b**, **1c**, **1d**, **1h**, **5**, **6b** and **6c**) equipped with a Mo Kα sealed tube (λ = 0.71073 Å), a multilayer TRIUMPH X-ray mirror, a Photon III-C14 detector. Phi- and omega-scans were used. Space group was determined on the basis of systematic absences and intensity statistics. Semi-empirical absorption correction was employed.[10] These structures were solved using an intrinsic phasing method (SHELXT),[11] and refined using the least-squares method on F^2.[12] All non-H atoms were refined with anisotropic displacement parameters. Hydrogen atoms were refined isotropically at calculated positions using a riding model with their isotropic displacement parameters constrained to be equal to 1.5 times the equivalent isotropic displacement parameters of their pivot atoms for terminal sp^3 carbon and 1.2 times for all other carbon atoms.

1,2,3,4,5,5-Hexaphenylcyclopenta-1,3-diene (1a):

Crystals suitable for X-Ray diffraction analysis were obtained by slow evaporation of a CH_2Cl_2/acetonitrile (3:1) solution of compound **1a**. The title compound appeared to co-crystalize with residual traces of heptane, used as solvent in a previous work-up step.
CCDC-2214598 contains the supplementary crystallographic data for this compound. These data can be obtained free of charge from The Cambridge Crystallographic Data Centre *via* https://www.ccdc.cam.ac.uk/structures.

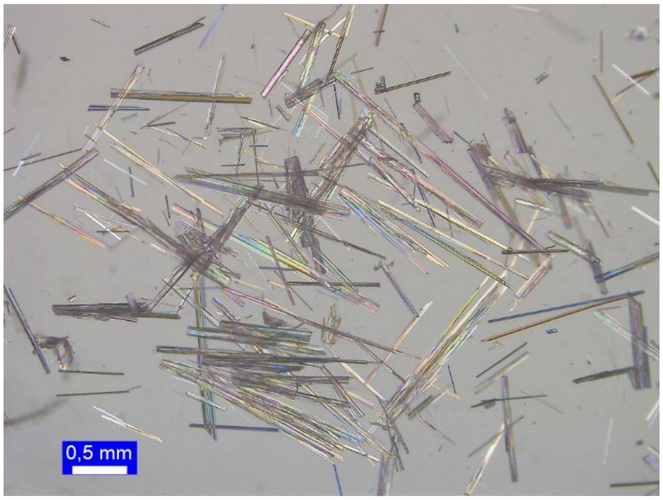

Figure S14. Picture of the crystals.

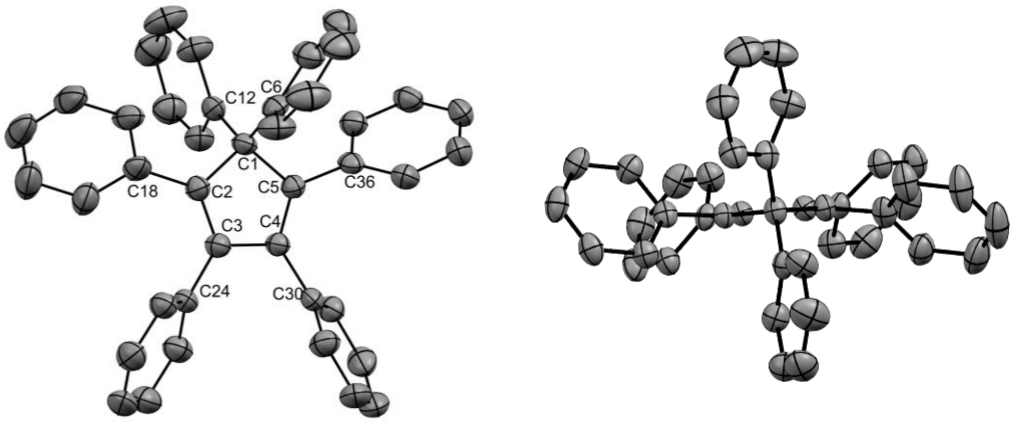

Figure S15. Top view (left) and side view (right) of the molecular structure of compound **1a**. Thermal ellipsoids are drawn at 50% probability level. Hydrogen atoms and solvent molecules (heptane) are omitted for clarity.

Table S9. Crystal data and structure refinement for compound **1a**.

Empirical formula	$C_{41}H_{30}$, ¼ (C_7H_{16})	
Formula weight	547.70	
Temperature	193(2) K	
Wavelength	0.71073 Å	
Crystal system	Monoclinic	
Space group	$P2_1/c$	
Unit cell dimensions	a = 20.709(3) Å	α= 90°.
	b = 14.9107(19) Å	β= 107.434(3)°.
	c = 22.817(3) Å	γ = 90°.
Volume	6722.1(14) Å^3	
Z	8	
Density (calculated)	1.082 Mg/m^3	
Absorption coefficient	0.061 mm^{-1}	
F(000)	2324	
Crystal size	0.200 x 0.120 x 0.040 mm^3	
Theta range for data collection	1.031 to 25.139°.	
Index ranges	-24<=h<=24, -17<=k<=17, -27<=l<=27	
Reflections collected	143928	
Independent reflections	11948 [R(int) = 0.1558]	
Completeness to theta = 25.139°	99.2 %	
Absorption correction	Semi-empirical from equivalents	
Max. and min. transmission	0.7342 and 0.6809	
Refinement method	Full-matrix least-squares on F^2	
Data / restraints / parameters	11948 / 281 / 866	
Goodness-of-fit on F^2	1.045	
Final R indices [I>2sigma(I)]	R1 = 0.0666, wR2 = 0.1665	
R indices (all data)	R1 = 0.1533, wR2 = 0.2174	
Extinction coefficient	n/a	
Largest diff. peak and hole	0.383 and -0.286 e.Å^{-3}	

1,2,3,4,5,5-Hexa(4’-*tert*-butylphenyl)cyclopenta-1,3-diene (1b):

Crystals suitable for X-Ray diffraction analysis were obtained by train sublimation of the pure compound **1b** under vacuum (7x10^{-2} mbar) at 250 °C to 290 °C over 2 h, under a nitrogen flow. After slow cooling of the sublimation tube (over 1h), crystals were recovered and directly analysed without further processing. One *tert*-butyl group was found to be disordered on 2 positions. Several restraints (SAME, SIMU, DELU) were applied to refine some moieties of the molecule and to avoid the collapse of the structure during the least-squares refinement by the large anisotropic displacement parameters.

CCDC-2214599 contains the supplementary crystallographic data for this compound. These data can be obtained free of charge from The Cambridge Crystallographic Data Centre *via* https://www.ccdc.cam.ac.uk/structures.

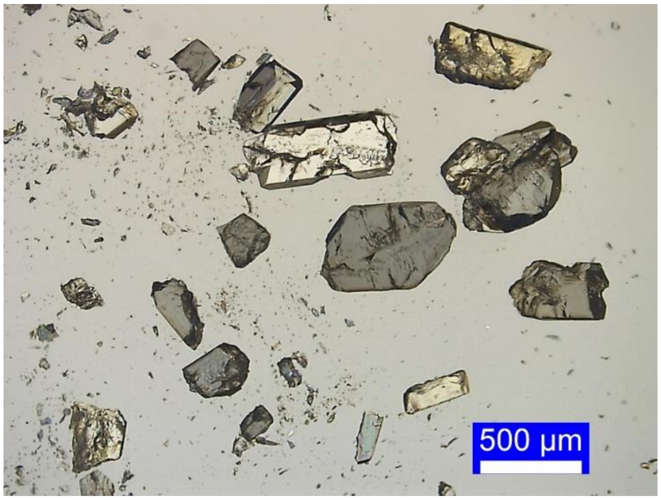

Figure S16. Picture of the crystals.

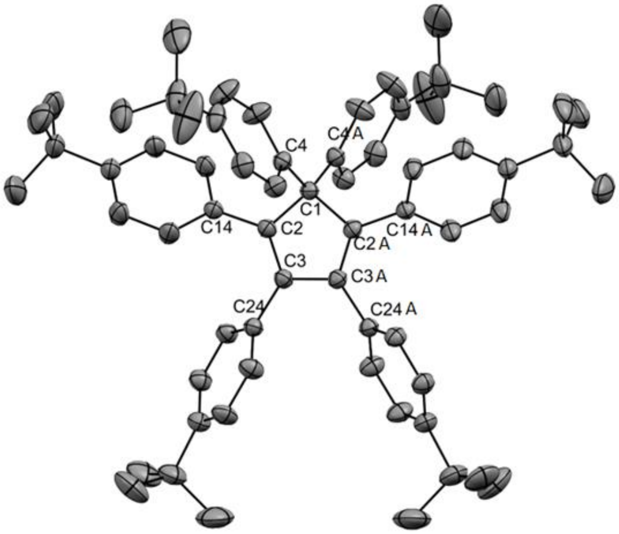

Figure S17. Molecular structure of compound **1b**. Thermal ellipsoids are drawn at 50% probability level. Hydrogen and disordered atoms are omitted for clarity.

Table S10. Crystal data and structure refinement for compound **1b**.

Empirical formula	$C_{65}H_{78}$	
Formula weight	859.27	
Temperature	193(2) K	
Wavelength	0.71073 Å	
Crystal system	Monoclinic	
Space group	C2/c	
Unit cell dimensions	a = 20.6440(13) Å	α= 90°.
	b = 13.3024(8) Å	β= 111.818(2)°.
	c = 21.865(2) Å	γ = 90°.
Volume	5574.3(7) $Å^3$	
Z	4	
Density (calculated)	1.024 Mg/m^3	
Absorption coefficient	0.057 mm^{-1}	
F(000)	1872	
Crystal size	0.260 x 0.250 x 0.220 mm^3	
Theta range for data collection	3.015 to 30.558°.	
Index ranges	-29<=h<=29, -18<=k<=19, -28<=l<=31	
Reflections collected	152698	
Independent reflections	8531 [R(int) = 0.0643]	
Completeness to theta = 25.242°	99.6 %	
Absorption correction	Semi-empirical from equivalents	
Max. and min. transmission	0.7461 and 0.7101	
Refinement method	Full-matrix least-squares on F^2	
Data / restraints / parameters	8531 / 136 / 337	
Goodness-of-fit on F^2	1.019	
Final R indices [I>2sigma(I)]	R1 = 0.0565, wR2 = 0.1462	
R indices (all data)	R1 = 0.0783, wR2 = 0.1645	
Extinction coefficient	n/a	
Largest diff. peak and hole	0.347 and -0.210 e.$Å^{-3}$	

1,2,3,4,5,5-Hexa(4'-tolyl)cyclopenta-1,3-diene (1c):

Crystals suitable for X-Ray diffraction analysis were obtained by train sublimation of the pure compound **1c** under vacuum (5x10^{-2} mbar) at 350 °C for 1h, under a nitrogen flow. After slow cooling of the sublimation tube, crystals were recovered and directly analysed without further processing.

CCDC-2214600 contains the supplementary crystallographic data for this compound. These data can be obtained free of charge from The Cambridge Crystallographic Data Centre *via* https://www.ccdc.cam.ac.uk/structures.

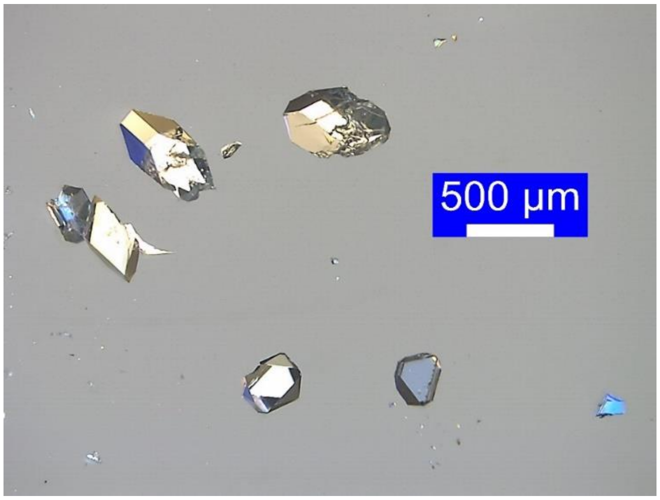

Figure S18. Picture of the crystals.

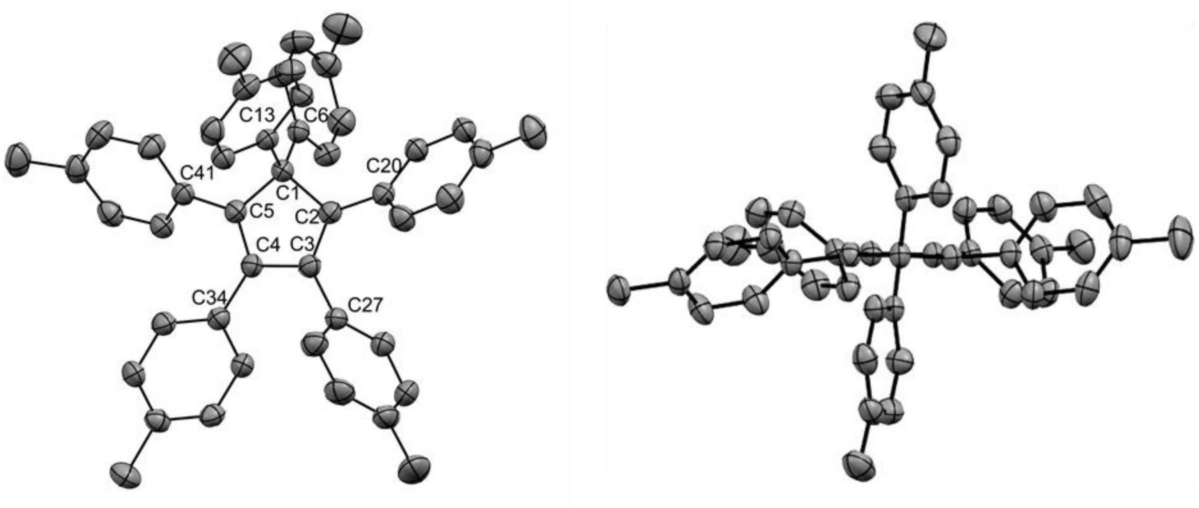

Figure S19. Top view (left) and side view (right) of the molecular structure of compound **1c**. The asymmetric unit contains two independent molecules, only one molecule is represented for the sake of clarity. Thermal ellipsoids are drawn at 50% probability level and hydrogen atoms are omitted for clarity.

Table S11. Crystal data and structure refinement for compound **1c**.

Empirical formula	$C_{47}H_{42}$	
Formula weight	606.80	
Temperature	193(2) K	
Wavelength	1.54178 Å	
Crystal system	Triclinic	
Space group	$P\ \bar{1}$	
Unit cell dimensions	a = 13.3948(6) Å	α= 100.057(2)°.
	b = 14.7194(6) Å	β= 93.842(2)°.
	c = 20.6475(9) Å	γ = 115.215(2)°.
Volume	3580.5(3) Å^3	
Z	4	
Density (calculated)	1.126 Mg/m^3	
Absorption coefficient	0.475 mm^{-1}	
F(000)	1296	
Crystal size	0.240 x 0.240 x 0.200 mm^3	
Theta range for data collection	3.406 to 72.847°.	
Index ranges	-16<=h<=16, -18<=k<=17, -25<=l<=25	
Reflections collected	71428	
Independent reflections	14176 [R(int) = 0.0512]	
Completeness to theta = 67.679°	99.8 %	
Absorption correction	Semi-empirical from equivalents	
Max. and min. transmission	0.7398 and 0.6970	
Refinement method	Full-matrix least-squares on F^2	
Data / restraints / parameters	14176 / 0 / 859	
Goodness-of-fit on F^2	1.026	
Final R indices [I>2sigma(I)]	R1 = 0.0586, wR2 = 0.1470	
R indices (all data)	R1 = 0.0819, wR2 = 0.1636	
Extinction coefficient	n/a	
Largest diff. peak and hole	0.270 and -0.346 e.Å^{-3}	

1,2,3,4,5,5-Hexa(3'-tolyl)cyclopenta-1,3-diene (1d):

Crystals suitable for X-Ray diffraction analysis were obtained by slow evaporation of a CH_2Cl_2/MeOH (3:1) solution of compound **1d**.

The methyl group of the aryl located on C2 is disordered over the 2 *meta*-positions. Several restraints (SIMU, DELU) and equal xyz and U_{ij} constraints (EXYZ and EADP) were applied to refine some moieties of the molecule and to avoid the collapse of the structure during the least-squares refinement by the large anisotropic displacement parameters. Some bond lengths were restrained with DFIX to suitable target values.

CCDC-2214601 contains the supplementary crystallographic data for this compound. These data can be obtained free of charge from The Cambridge Crystallographic Data Centre *via* https://www.ccdc.cam.ac.uk/structures.

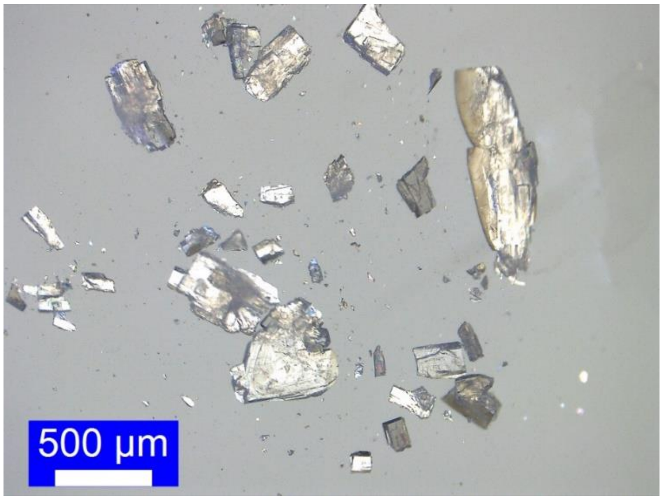

Figure S20. Picture of the crystals.

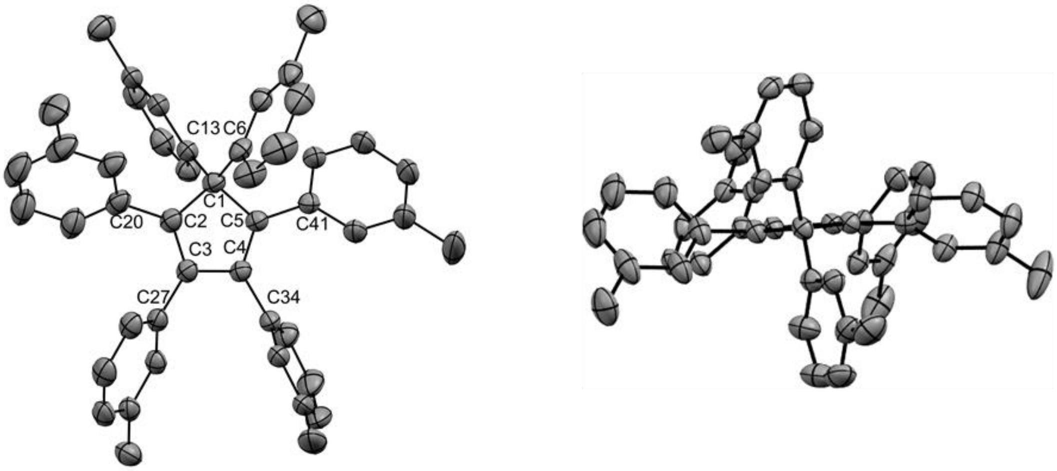

Figure S21. Top view (left) and side view (right) of the molecular structure of compound **1d**. Thermal ellipsoids are drawn at 50% probability level. Hydrogen and disordered atoms are omitted for clarity.

Table S12. Crystal data and structure refinement for compound **1d**.

Empirical formula	$C_{47}H_{42}$	
Formula weight	606.80	
Temperature	193(2) K	
Wavelength	0.71073 Å	
Crystal system	Triclinic	
Space group	$P\bar{1}$	
Unit cell dimensions	a = 11.2444(6) Å	α= 88.4787(18)°.
	b = 11.5920(6) Å	β= 85.8921(18)°.
	c = 15.4587(8) Å	γ = 62.0337(16)°.
Volume	1775.04(16) Å^3	
Z	2	
Density (calculated)	1.135 Mg/m^3	
Absorption coefficient	0.064 mm^{-1}	
F(000)	648	
Crystal size	0.300 x 0.200 x 0.200 mm^3	
Theta range for data collection	3.237 to 28.305°.	
Index ranges	-14<=h<=14, -14<=k<=15, -20<=l<=20	
Reflections collected	58918	
Independent reflections	8807 [R(int) = 0.0479]	
Completeness to theta = 25.242°	99.5 %	
Absorption correction	Semi-empirical from equivalents	
Max. and min. transmission	0.7457 and 0.7179	
Refinement method	Full-matrix least-squares on F^2	
Data / restraints / parameters	8807 / 0 / 439	
Goodness-of-fit on F^2	1.038	
Final R indices [I>2sigma(I)]	R1 = 0.0571, wR2 = 0.1437	
R indices (all data)	R1 = 0.0836, wR2 = 0.1624	
Extinction coefficient	n/a	
Largest diff. peak and hole	0.416 and -0.210 e.Å^{-3}	

1,2,3,4,5,5-Hexa(4'-methoxyphenyl)cyclopenta-1,3-diene (1h):

Crystals suitable for X-Ray diffraction analysis were obtained by slow evaporation of a CH_2Cl_2/MeOH (3:1) solution of compound **1h**.

CCDC-2214602 contains the supplementary crystallographic data for this compound. These data can be obtained free of charge from The Cambridge Crystallographic Data Centre *via* https://www.ccdc.cam.ac.uk/structures.

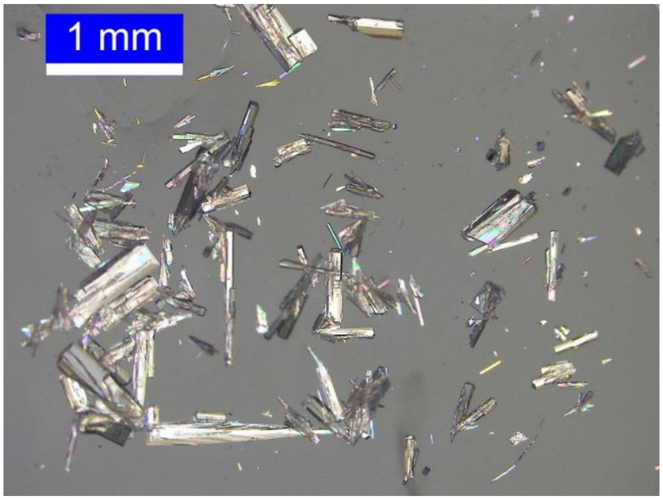

Figure S22. Picture of the crystals.

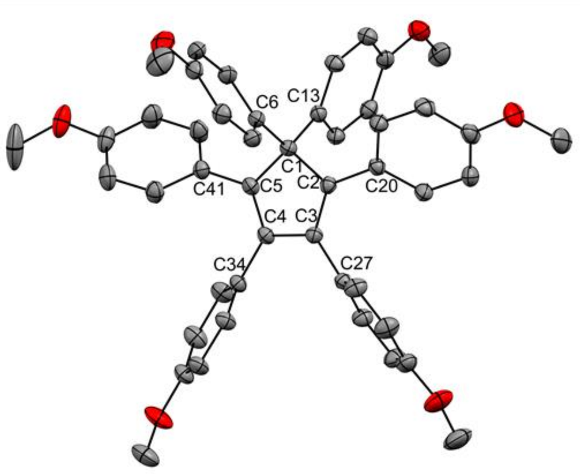

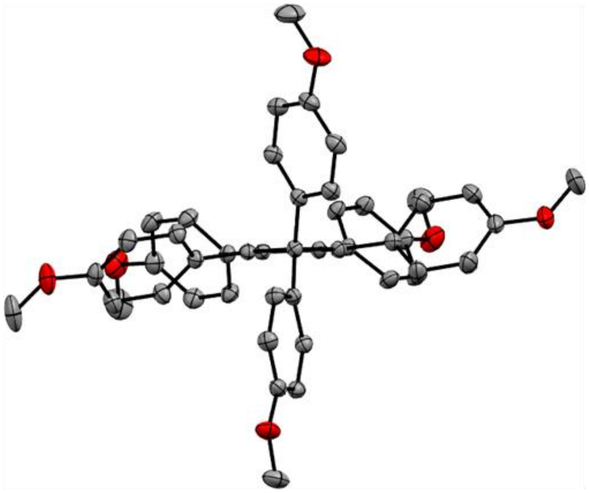

Figure S23. Top view (left) and side view (right) of the molecular structure of compound **1h**. Thermal ellipsoids are drawn at 50% probability level and hydrogen atoms are omitted for clarity.

Table S13. Crystal data and structure refinement for compound **1h**.

Empirical formula	$C_{47}H_{42}O_6$	
Formula weight	702.80	
Temperature	193(2) K	
Wavelength	0.71073 Å	
Crystal system	Monoclinic	
Space group	$P2_1/n$	
Unit cell dimensions	a = 10.9046(6) Å	α= 90°.
	b = 14.0514(6) Å	β= 94.090(2)°.
	c = 24.7528(15) Å	γ = 90°.
Volume	3783.1(3) $Å^3$	
Z	4	
Density (calculated)	1.234 Mg/m^3	
Absorption coefficient	0.081 mm^{-1}	
F(000)	1488	
Crystal size	0.400 x 0.200 x 0.200 mm^3	
Theta range for data collection	2.809 to 30.562°.	
Index ranges	-15<=h<=15, -20<=k<=20, -35<=l<=35	
Reflections collected	152450	
Independent reflections	11584 [R(int) = 0.0556]	
Completeness to theta = 25.242°	99.6 %	
Absorption correction	Semi-empirical from equivalents	
Max. and min. transmission	0.7461 and 0.7036	
Refinement method	Full-matrix least-squares on F^2	
Data / restraints / parameters	11584 / 0 / 484	
Goodness-of-fit on F^2	1.033	
Final R indices [I>2sigma(I)]	R1 = 0.0572, wR2 = 0.1364	
R indices (all data)	R1 = 0.0748, wR2 = 0.1488	
Extinction coefficient	n/a	
Largest diff. peak and hole	0.429 and -0.250 e.$Å^{-3}$	

1,1,2,3-Tetraphenyl-*1H*-cyclopenta[*l*]phenanthrene (5):

Crystals suitable for X-Ray diffraction analysis were obtained by train sublimation of the pure compound **5** under vacuum ($5x10^{-2}$ mbar) at 300 °C for 1h, under a nitrogen flow. After slow cooling (1h) of the sublimation tube, crystals were recovered and directly analysed without further processing.

CCDC-2214603 contains the supplementary crystallographic data for this compound. These data can be obtained free of charge from The Cambridge Crystallographic Data Centre *via* https://www.ccdc.cam.ac.uk/structures.

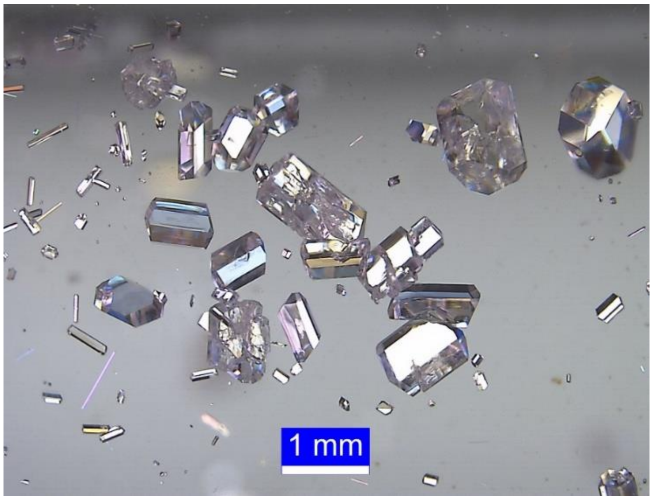

Figure S24. Picture of the crystals.

Table S14. Crystal data and structure refinement for compound **5**.

Empirical formula	$C_{41}H_{28}$	
Formula weight	520.63	
Temperature	193(2) K	
Wavelength	0.71073 Å	
Crystal system	Triclinic	
Space group	$P\bar{1}$	
Unit cell dimensions	a = 9.6243(3) Å	α= 90.8439(16)°.
	b = 11.6276(5) Å	β= 106.9556(14)°.
	c = 13.5153(6) Å	γ = 109.2957(14)°.
Volume	1355.07(9) $Å^3$	
Z	2	
Density (calculated)	1.276 Mg/m^3	
Absorption coefficient	0.072 mm^{-1}	
F(000)	548	
Crystal size	0.400 x 0.300 x 0.050 mm^3	
Theta range for data collection	3.183 to 28.297°.	
Index ranges	-12<=h<=12, -15<=k<=15, -18<=l<=18	
Reflections collected	44286	
Independent reflections	6677 [R(int) = 0.0354]	
Completeness to theta = 25.242°	99.0 %	
Absorption correction	Semi-empirical from equivalents	
Max. and min. transmission	0.7457 and 0.7190	
Refinement method	Full-matrix least-squares on F^2	
Data / restraints / parameters	6677 / 0 / 370	
Goodness-of-fit on F^2	1.053	
Final R indices [I>2sigma(I)]	R1 = 0.0478, wR2 = 0.1234	
R indices (all data)	R1 = 0.0558, wR2 = 0.1300	
Extinction coefficient	n/a	
Largest diff. peak and hole	0.360 and -0.239 e.$Å^{-3}$	

3,6,11,14-Tetra(*tert*-butyl)-17,17-di(4’-*tert*-butylphenyl)cyclopenta[*l*,*l’*]diphenanthrene (6b):

Crystals suitable for X-Ray diffraction analysis were obtained by slow evaporation of a CH_2Cl_2/MeOH solution of compound **6b.** The title compound co-crystalised with dichloromethane.

Most of the *tert*-butyl groups are disordered over two positions. Several restraints (SAME, SIMU, DELU, ISOR) were applied to refine some moieties of the molecule and to avoid the collapse of the structure during the least-squares refinement by the large anisotropic displacement parameters.

CCDC-2214604 contains the supplementary crystallographic data for this compound. These data can be obtained free of charge from The Cambridge Crystallographic Data Centre *via* https://www.ccdc.cam.ac.uk/structures.

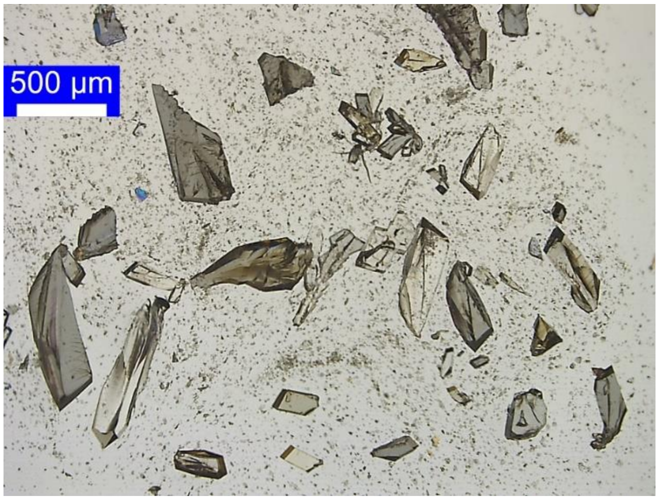

Figure S25. Picture of the crystals.

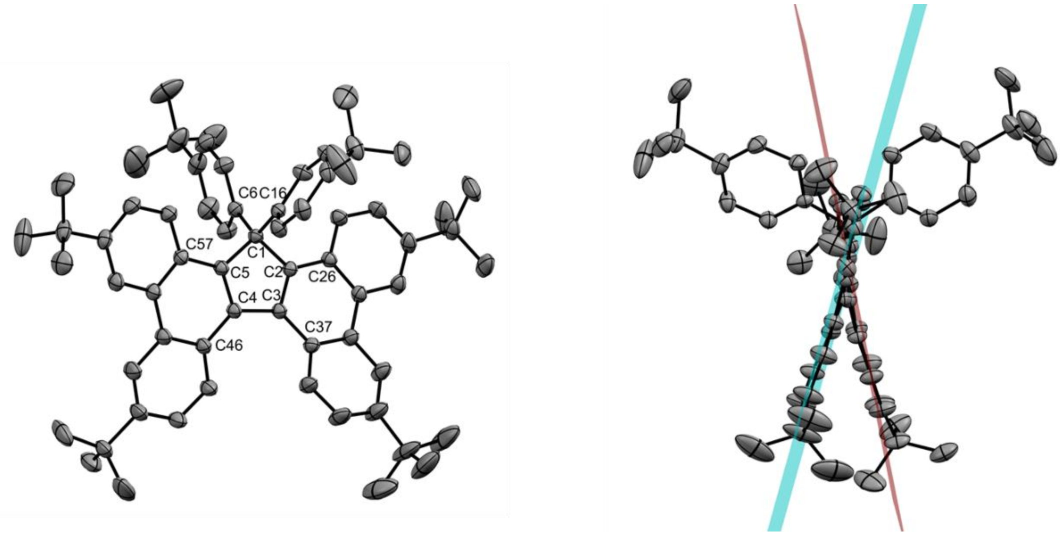

Figure S26. Top view (left) and side view (right) of the molecular structure of compound **6b**. On the side view, the 27° distortion between both phenanthrene units is highlighted thanks to the representation in blue and red, respectively, of both phenanthrene mean planes. Thermal ellipsoids are drawn at 50% probability level. Hydrogen and disordered atoms and solvent molecules are omitted for clarity.

Table S15. Crystal data and structure refinement for compound **6b**.

Empirical formula	$C_{65}H_{74}$, ¼ (CH_2Cl_2)	
Formula weight	876.47	
Temperature	193(2) K	
Wavelength	0.71073 Å	
Crystal system	Triclinic	
Space group	$P\ \bar{1}$	
Unit cell dimensions	a = 12.2669(7) Å	α= 106.574(2)°.
	b = 12.7915(6) Å	β= 99.595(2)°.
	c = 18.0256(9) Å	γ = 90.608(2)°.
Volume	2667.9(2) $Å^3$	
Z	2	
Density (calculated)	1.091 Mg/m^3	
Absorption coefficient	0.085 mm^{-1}	
F(000)	949	
Crystal size	0.500 x 0.350 x 0.200 mm^3	
Theta range for data collection	2.674 to 28.310°.	
Index ranges	-16<=h<=16, -17<=k<=17, -24<=l<=24	
Reflections collected	92592	
Independent reflections	13219 [R(int) = 0.0416]	
Completeness to theta = 25.242°	99.4 %	
Absorption correction	Semi-empirical from equivalents	
Max. and min. transmission	0.7457 and 0.7246	
Refinement method	Full-matrix least-squares on F^2	
Data / restraints / parameters	13219 / 652 / 792	
Goodness-of-fit on F^2	1.043	
Final R indices [I>2sigma(I)]	R1 = 0.0593, wR2 = 0.1594	
R indices (all data)	R1 = 0.0767, wR2 = 0.1743	
Extinction coefficient	n/a	
Largest diff. peak and hole	0.334 and -0.257 e.$Å^{-3}$	

3,6,11,14-Tetramethyl-17,17-di(4'-methylphenyl)cyclopenta[*l*,*l'*]diphenanthrene (6c):

Crystals suitable for X-Ray diffraction analysis were obtained by slow evaporation of a CH_2Cl_2/MeOH solution of compound **6c.**

Some residual electron densities were difficult to modelize and therefore, the SQUEEZE function of PLATON[13] was used to eliminate the contribution of the electron density in the solvent region from the intensity data, and the solvent-free model was employed for the final refinement.

CCDC-2214605 contains the supplementary crystallographic data for this compound. These data can be obtained free of charge from The Cambridge Crystallographic Data Centre *via* https://www.ccdc.cam.ac.uk/structures.

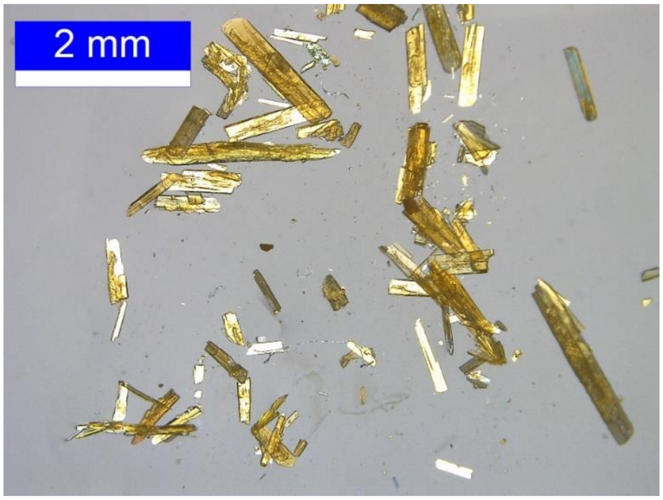

Figure S27. Picture of the crystals.

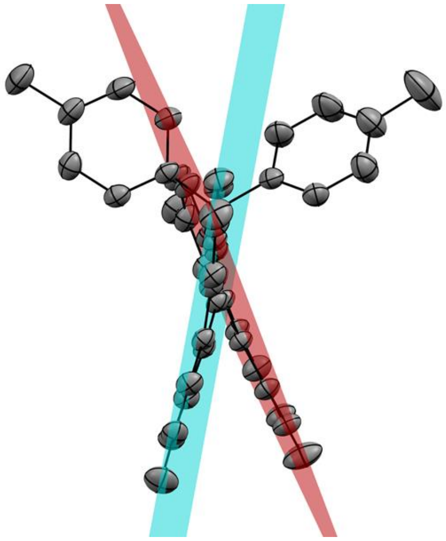

Figure S28. Side view of the molecular structure of compound **6c**: the 34° distortion between both phenanthrene units is highlighted thanks to the representation in blue and red, respectively, of both phenanthrene mean planes. Thermal ellipsoids are drawn at 50% probability level and hydrogen atoms are omitted for clarity.

Table S16. Crystal data and structure refinement for compound **6c**.

Empirical formula	$C_{47}H_{38}$	
Formula weight	602.77	
Temperature	193(2) K	
Wavelength	0.71073 Å	
Crystal system	Triclinic	
Space group	$P\ \bar{1}$	
Unit cell dimensions	a = 10.6181(5) Å	α= 100.650(2)°.
	b = 10.9815(6) Å	β= 106.0590(19)°.
	c = 16.4791(9) Å	γ = 91.5785(19)°.
Volume	1808.41(16) $Å^3$	
Z	2	
Density (calculated)	1.107 Mg/m^3	
Absorption coefficient	0.062 mm^{-1}	
F(000)	640	
Crystal size	0.400 x 0.100 x 0.060 mm^3	
Theta range for data collection	2.511 to 28.323°.	
Index ranges	-14<=h<=13, -14<=k<=14, -21<=l<=21	
Reflections collected	85443	
Independent reflections	8989 [R(int) = 0.0853]	
Completeness to theta = 25.242°	99.9 %	
Absorption correction	Semi-empirical from equivalents	
Max. and min. transmission	0.7374 and 0.6944	
Refinement method	Full-matrix least-squares on F^2	
Data / restraints / parameters	8989 / 0 / 430	
Goodness-of-fit on F^2	1.016	
Final R indices [I>2sigma(I)]	R1 = 0.0575, wR2 = 0.1530	
R indices (all data)	R1 = 0.1002, wR2 = 0.1796	
Extinction coefficient	n/a	
Largest diff. peak and hole	0.278 and -0.196 e.$Å^{-3}$	

5-(4'-Fluorophenyl)-1,2,3,4,5-pentaphenylcyclopenta-1,3-diene (7, as a mixture of regioisomers):

Crystals suitable for X-Ray diffraction analysis were obtained by slow diffusion of methanol in a concentrated CH_2Cl_2 solution of compound **7** (as a 73:18:9 mixture of regioisomers in solution).

The obtained structure is very complicated. The unit cell contains 4 independent molecules in which the F atom occupies the *para* position of several phenyl groups in variable ratios (sum of the ratios is equal to 100%, each molecule contains 1 F atom). Several restraints (SAME, SIMU, DELU, ISOR) and equal xyz and U_{ij} constraints (EXYZ and EADP) were applied to refine some moieties of the molecule and to avoid the collapse of the structure during the least-squares refinement by the large anisotropic displacement parameters.

CCDC-2214606 contains the supplementary crystallographic data for this compound. These data can be obtained free of charge from The Cambridge Crystallographic Data Centre *via* https://www.ccdc.cam.ac.uk/structures.

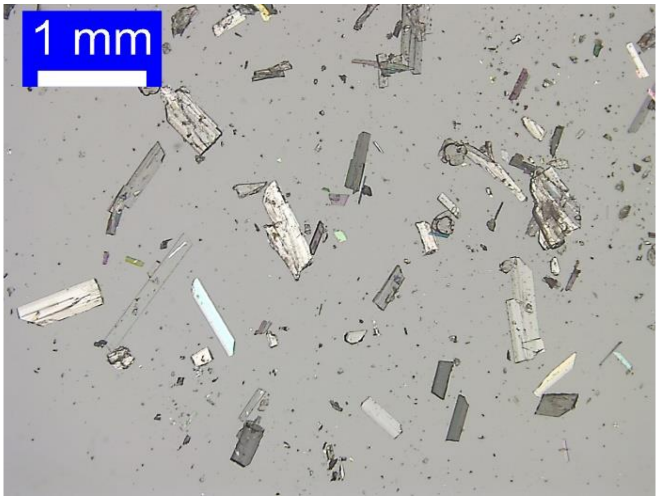

Figure S29. Picture of the crystals.

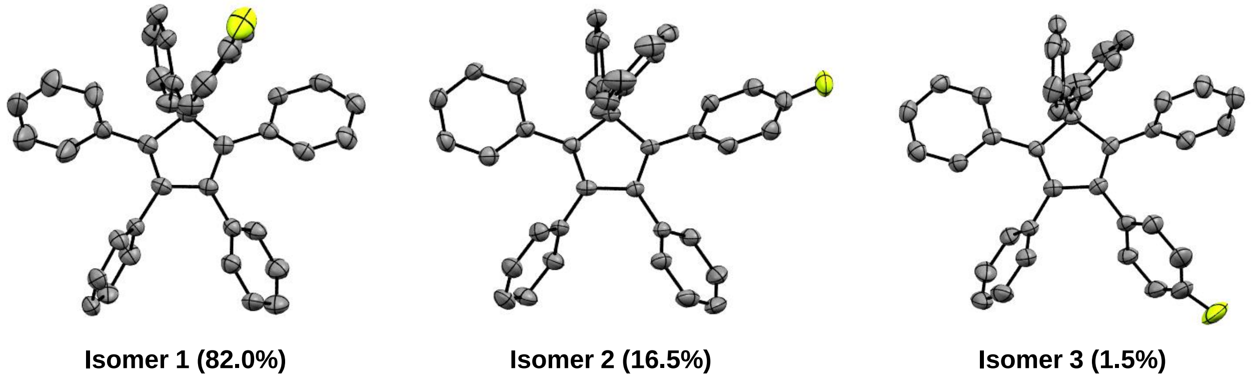

Figure S30. Molecular structure of compound **7**, as a 82.0 : 16.5 : 1.5 mixture of regioisomers in the diffracted crystal. Thermal ellipsoids are drawn at 50% probability level. Hydrogen and disordered atoms are omitted for clarity.

Table S17. Crystal data and structure refinement for compound **7**.

Empirical formula	$C_{41}H_{29}F$	
Formula weight	540.64	
Temperature	193(2) K	
Wavelength	0.71073 Å	
Crystal system	Triclinic	
Space group	$P\ \bar{1}$	
Unit cell dimensions	a = 10.256(2) Å	α= 94.02(3)°.
	b = 20.262(4) Å	β= 93.18(3)°.
	c = 29.739(6) Å	γ = 103.46(3)°.
Volume	5979(2) Å^3	
Z	8	
Density (calculated)	1.201 Mg/m^3	
Absorption coefficient	0.072 mm^{-1}	
F(000)	2272	
Crystal size	0.400 x 0.200 x 0.120 mm^3	
Theta range for data collection	0.688 to 26.407°.	
Index ranges	-12<=h<=12, -25<=k<=25, 0<=l<=37	
Reflections collected	40152	
Independent reflections	24408 [R(int) = 0.0633]	
Completeness to theta = 25.242°	99.4 %	
Absorption correction	Semi-empirical from equivalents	
Max. and min. transmission	0.745372 and 0.536688	
Refinement method	Full-matrix least-squares on F^2	
Data / restraints / parameters	24408 / 854 / 1605	
Goodness-of-fit on F^2	1.060	
Final R indices [I>2sigma(I)]	R1 = 0.0817, wR2 = 0.1658	
R indices (all data)	R1 = 0.1758, wR2 = 0.2102	
Extinction coefficient	n/a	
Largest diff. peak and hole	0.349 and -0.317 e.Å^{-3}	

VIII. References

[1] M. P. Castellani, J. M. Wright, S. J. Geib, A. L. Rheingold and W. C. Trogler, Synthesis, molecular structure, and 2-D NMR analysis of bis(tetraphenylcyclopentadienyl)iron(II), *Organometallics*, 1986, **5**, 1116-1122.

[2] L. Bonnafoux, R. Gramage-Doria, F. Colobert and F. R. Leroux, Catalytic Palladium Phosphination: Modular Synthesis of C_1-Symmetric Biaryl-Based Diphosphines, *Chem. Eur. J.*, 2011, **17**, 11008-11016.

[3] A. Klapars and S. L. Buchwald, Copper-Catalyzed Halogen Exchange in Aryl Halides: An Aromatic Finkelstein Reaction, *J. Am. Chem. Soc.,* 2002, **124**, 14844-14845.

[4] G. Dyker, J. Heiermann, M. Miura, J.-I. Inoh, S. Pivsa-Art, T. Satoh and M. Nomura, Palladium-Catalyzed Arylation of Cyclopentadienes, *Chem. Eur. J.*, 2000, **6**, 3426-3433.

[5] (a) D. S. Surry and S. L. Buchwald, Diamine ligands in copper-catalyzed reactions, *Chem. Sci.,* 2010, **1**, 13-31; (b) I. P. Beletskaya and A. Y. Fedorov, Modern Copper-Catalyzed Hurtley Reaction: Efficient C-Arylation of CH-Acid Derivatives, In *Copper-Mediated Cross-Coupling Reactions*, ed. G. Evano and N. Blanchard, John Wiley & Sons, Hoboken, New Jersey, USA, 2014, Ch. 8, pp 283-311.

[6] (a) C. Sambiagio, S. P. Marsden, A. J. Blacker and P. C. McGowan, Copper catalysed Ullmann type chemistry: from mechanistic aspects to modern development, *Chem. Soc. Rev.,* 2014, **43**, 3525-3550; (b) S. Zhang, D. Zhang and L. S. Liebeskind, Ambient Temperature, Ullmann-like Reductive Coupling of Aryl, Heteroaryl, and Alkenyl Halides, *J. Org. Chem.,* 1997, **62**, 2312-2313.

[7] V. Pace, P. Hoyos, L. Castoldi, P. Domínguez de María and A. R. Alcántara, 2-Methyltetrahydrofuran (2-MeTHF): A Biomass-Derived Solvent with Broad Application in Organic Chemistry, *ChemSusChem,* 2012, **5**, 1369-1379.

[8] (a) D. Stuerga and P. Gaillard, Microwave heating as a new way to induce localized enhancements of reaction rate. Non-isothermal and heterogeneous kinetics, *Tetrahedron,* 1996, **52**, 5505-5510; (b) S. Horikoshi, A. Osawa, M. Abe and N. Serpone, On the Generation of Hot-Spots by Microwave Electric and Magnetic Fields and Their Impact on a Microwave-Assisted Heterogeneous Reaction in the Presence of Metallic Pd Nanoparticles on an Activated Carbon Support, *J. Phys. Chem. C,* 2011, **115**, 23030-23035; (c) W. Wang, B. Wang, J. Sun, Y. Mao, X. Zhao and Z. Song, Numerical simulation of hot-spot effects in microwave heating due to the existence of strong microwave-absorbing media, *RSC Adv.,* 2016, **6**, 52974-52981.

[9] M. A. Herrero, J. M. Kremsner and C. O. Kappe, Nonthermal Microwave Effects Revisited: On the Importance of Internal Temperature Monitoring and Agitation in Microwave Chemistry, *J. Org. Chem.,* 2008, **73**, 36-47.

[10] Bruker, SADABS, Bruker AXS Inc., Madison, Wisconsin, USA, 2008.

[11] ShelXT, G. M. Sheldrick, University of Göttingen, *Acta Crystallogr. Sect. A,* 2015, **71**, 3-8.

[12] ShelXL, G. M. Sheldrick, University of Göttingen, *Acta Crystallogr. Sect. C,* 2015, **71**, 3-8.

[13] A. L. Spek, *Acta Crystallogr. Sect. C,* 2015, **71**, 9-18.
